# Maternal high-fat diet associated with altered gene expression, DNA methylation, and obesity risk in mouse offspring

**DOI:** 10.1371/journal.pone.0192606

**Published:** 2018-02-15

**Authors:** Madeline Rose Keleher, Rabab Zaidi, Shyam Shah, M. Elsa Oakley, Cassondra Pavlatos, Samir El Idrissi, Xiaoyun Xing, Daofeng Li, Ting Wang, James M. Cheverud

**Affiliations:** 1 Department of Evolution, Ecology, and Population Biology, Washington University in St. Louis, St. Louis, Missouri, United States of America; 2 Department of Biology, Loyola University, Chicago, Illinois, United States of America; 3 Department of Genetics, Washington University in St. Louis, St. Louis, Missouri, United States of America; University of Missouri Columbia, UNITED STATES

## Abstract

We investigated maternal obesity in inbred SM/J mice by assigning females to a high-fat diet or a low-fat diet at weaning, mating them to low-fat-fed males, cross-fostering the offspring to low-fat-fed SM/J nurses at birth, and weaning the offspring onto a high-fat or low-fat diet. A maternal high-fat diet exacerbated obesity in the high-fat-fed daughters, causing them to weigh more, have more fat, and have higher serum levels of leptin as adults, accompanied by dozens of gene expression changes and thousands of DNA methylation changes in their livers and hearts. Maternal diet particularly affected genes involved in RNA processing, immune response, and mitochondria. Between one-quarter and one-third of differentially expressed genes contained a differentially methylated region associated with maternal diet. An offspring high-fat diet reduced overall variation in DNA methylation, increased body weight and organ weights, increased long bone lengths and weights, decreased insulin sensitivity, and changed the expression of 3,908 genes in the liver. Although the offspring were more affected by their own diet, their maternal diet had epigenetic effects lasting through adulthood, and in the daughters these effects were accompanied by phenotypic changes relevant to obesity and diabetes.

## Introduction

A mother’s diet—from its fat and protein content to its richness in methyl donors while DNA methylation is being established in the developing fetus—can directly affect her offspring’s epigenome [1–4]. Maternal diet is important to study since half of United States mothers have pre-pregnancy weights classifying them as overweight or obese [5]. Babies of obese women have a higher risk of stillbirth [6, 7], neural-tube defects [8, 9], and are born with more body fat, more leptin in their cord blood, and increased inflammatory cytokine levels [10]. In children, maternal obesity raises the risk of obesity [11–13]; it is associated with higher levels of insulin, cholesterol, and blood pressure [14]; and it increases the risk of neuropsychiatric problems [15] and impaired cognitive and executive function [16]. The effects of maternal obesity continue into adulthood, elevating the risk of cancer, type 2 diabetes, cardiovascular disease, and high blood pressure [17–20].

The effects of maternal obesity and high-fat diet in mice are similar to those in humans. C57BL/6J and C57BL/6NCrl mice exposed to a high-fat diet during gestation have been shown to weigh more and exhibit hyperglycemia, hypertension, fatty livers, and insulin resistance as adults, even when fed a standard diet after birth [21, 22]. Some of the phenotypic changes induced by maternal high-fat diet have been linked to epigenetics and gene expression. For instance, offspring of high-fat-fed C57BL/6J mothers were found to have larger livers exhibiting inflammation and steatosis, accompanied by higher profibrogenic gene expression and 82 differentially methylated regions [23]. Altered gene expression due to maternal obesity has also been found in the hearts of C57BL/6J mice, along with cardiac dysfunction [24, 25].

Previous rodent studies have revealed that offspring sex affects the response to maternal diet, with daughters of high-fat-fed mothers having higher blood pressure [26], higher plasma leptin levels [27], and smaller livers than sons [28], whereas sons have a more pronounced difference in their transcriptomes [29]. It is thus important to take offspring sex into account when investigating the phenotypic and epigenetic effects of maternal high-fat diet.

Maternal obesity impacts offspring disease risk through multiple mechanisms across the spectrum of development, from altering the glucose consumption and size of oocytes to disrupting the circadian rhythms and metabolic genes in offspring [30–32]. On the cellular level, maternal obesity can have lasting effects on offspring by changing the number, structure, and function of mitochondria in oocytes [33–35]. At the level of the organ, obesity affects offspring through placental abnormalities, including heavier placental weight, more inflammatory lesions [36], lower rates of apoptosis, increased transport of glucose and amino acids [37], and increased inflammatory cytokine expression [38].

Although obesity can be passed on to offspring via genetic variants, there is growing evidence that prenatal epigenetic programming plays a substantial role in the transmission of obesity across generations [4]. A classic example of this phenomenon is the *A*^*v*y^ allele at the agouti locus in mice. *A*^*v*y^/a dams give birth to pups with ectopic agouti expression, which causes obesity. However, when a pregnant *A*^*v*y^/a dam is fed a diet rich in methyl-donors, her offspring are born with increased agouti methylation, restored agouti expression, and they do not develop obesity, thus illustrating an epigenetic route for the enduring effects of maternal diet [1, 2]. In humans, newborns with obese parents have altered methylation of several imprinted genes in their cord blood [39], and children born after their mothers underwent gastric bypass surgery have lower Body Mass Indexes than their siblings born before the surgery, along with methylation differences in more than 5,000 genes in their blood [40]. Much remains to be learned about the epigenetic changes induced by maternal obesity. Since the effects of maternal obesity begin before birth, it is crucial to know which specific epigenetic risk factors a baby is likely to be born with in order to make early intervention possible.

It can be difficult to disentangle the effects of prenatal and postnatal maternal obesity in humans, so in this study we used a cross-fostering design in mice where offspring of high-fat-fed and low-fat-fed mothers were all fostered at birth to low-fat-fed nurses. Most maternal obesity research in mice focuses on the C57BL/6 strain, but because epigenetic response can be greatly dependent on genomic background, we used the less-studied SM/J strain, which is highly responsive to dietary fat.

## Results

### Obesity phenotypes

When the dams were mated at 10 weeks of age to produce the F_1_ offspring, the low-fat (LF) diet dams weighed on average 15.3 grams and the high-fat (HF) diet dams weighed 21.7 grams. We consider this 40% increase in weight of the HF dams, over 3 standard deviations higher than the LF dams, to signify obesity. A general linear model showed that the weekly weights, diabetes-related traits, and necropsy traits were all significantly affected by maternal diet (F_36,32_ = 3.0, p = 0.001), offspring diet (F_36,32_ = 28.2, p = 4.17 x 10^−16^), sex (F_36,32_ = 18.4, p = 2.36 x 10^−13^), and an offspring-diet-by-sex interaction (F_36,32_ = 4.4, p = 3.0 x 10^−5^) (Table 1). Due to the strong offspring-diet-by-sex interaction, sons and daughters were analyzed separately.

**Table 1:** The effect of maternal diet and a maternal-diet-by-offspring-diet interaction on daughters. A high-fat maternal diet increased the body weights of high-fat-fed daughters in adulthood, led to more brown fat, a borderline significant increase in reproductive fat pad weight, and increased serum levels of leptin. However, a high-fat maternal diet actually decreased levels of glucose and triglycerides in the daughters. HF = High-fat diet, LF = Low-fat diet, Mat = Maternal, Off = Offspring, N = 10 per group, averages given ± standard error.

Trait	HF-HF ♀	LF-HF ♀	HF-LF♀	LF-LF ♀	Mat Diet p-value	Mat Diet*Off Diet p-value
Week 9 weight (g)	17.44 ± 0.40	15.91 ± 0.55	12.05 ± 0.34	10.88 ± 1.02	**0.041**	0.781
Week 11 weight (g)	21.86±1.09	18.41±0.68	13.01±0.35	12.84±0.52	**0.016**	**0.028**
Week 12 weight (g)	23.69±1.32	19.86±0.91	12.25±0.97	13.19±0.37	0.138	**0.017**
Week 13 weight (g)	25.96±1.45	19.38±1.12	13.66±0.39	13.67±0.47	**0.002**	**0.002**
Week 14 weight (g)	27.11±1.58	22.33±1.09	13.85±0.35	14.02±0.46	**0.028**	**0.019**
Week 15 weight (g)	29.63±1.67	23.32±1.12	14.35±0.46	14.58±0.45	**0.007**	**0.004**
Week 17 weight (g)	30.73 ± 1.64	25.66 ± 1.11	14.53 ± 0.43	15.62 ± 0.37	0.062	**0.005**
Liver weight (g)	1.38 ± 0.22	0.87 ± 0.13	0.40 ± 0.03	0.53 ± 0.02	0.231	**0.034**
Fat pad weight (g)	1.42 ± 0.15	0.91 ± 0.12	0.10 ± 0.02	0.15 ± 0.03	**0.018**	**0.005**
Kidney weight (g)	0.19 ± 0.02	0.18 ± 0.01	0.11 ± 0.01	0.14 ± 0.01	0.606	**0.042**
Brown fat weight (g)	0.66 ± 0.08	0.32 ± 0.04	0.10 ± 0.01	0.11 ± 0.02	**0.001**	**0.001**
Serum leptin (ng/mL)	26.82 ± 5.57	12.94 ± 1.30	0.84 ± 0.18	2.82 ± 0.64	**0.050**	**0.011**
Serum insulin (pg/mL)	2184.30 ± 488.34	1120.67 ± 225.60	177.50 ± 31.77	348.10 ± 55.51	0.111	**0.030**
Serum glucose (mg/dL)	288.44 ± 15.84	361.44 ± 20.41	144.60 ± 18.06	301.77 ± 17.17	**1.99E-07**	**0.024**
Serum free fatty acids (mM)	1.42 ± 0.08	1.47 ± 0.09	2.06 ± 0.25	1.41 ± 0.05	**0.045**	**0.019**
Serum triglycerides (mg/dL)	121.48 ± 7.62	206.02 ± 31.48	97.76 ± 5.23	145.26 ± 12.08	**3.00E-04**	0.268

Unsurprisingly, the offspring’s diet had a major effect on the obesity traits. Compared to LF offspring, HF offspring had heavier body weights within one week of being weaned onto the diet (♀F_17,20_ = 29.3, p = 1.66 x 10^−10^, ♂ F_17,22_ = 40.9, p = 9.10 x 10^−13^), higher values for the diabetes-related traits (♀F_7,30_ = 33.6, p = 1.64 x 10^−12^, ♂ F_7,29_ = 48.4, p = 2.69 x 10^−14^), and heavier organs and higher levels of serum biomarkers (♀F_15,21_ = 12.6, p = 2.79 x 10^−7^, ♂ F_15,17_ = 42.8, p = 1.59 x 10^−10^) (Tables A-C in S1 File). HF offspring also had longer, heavier long bones (Tables D-E in S1 File), consistent with the notion that obesity alters bone metabolism [41].

**Fig 1:**
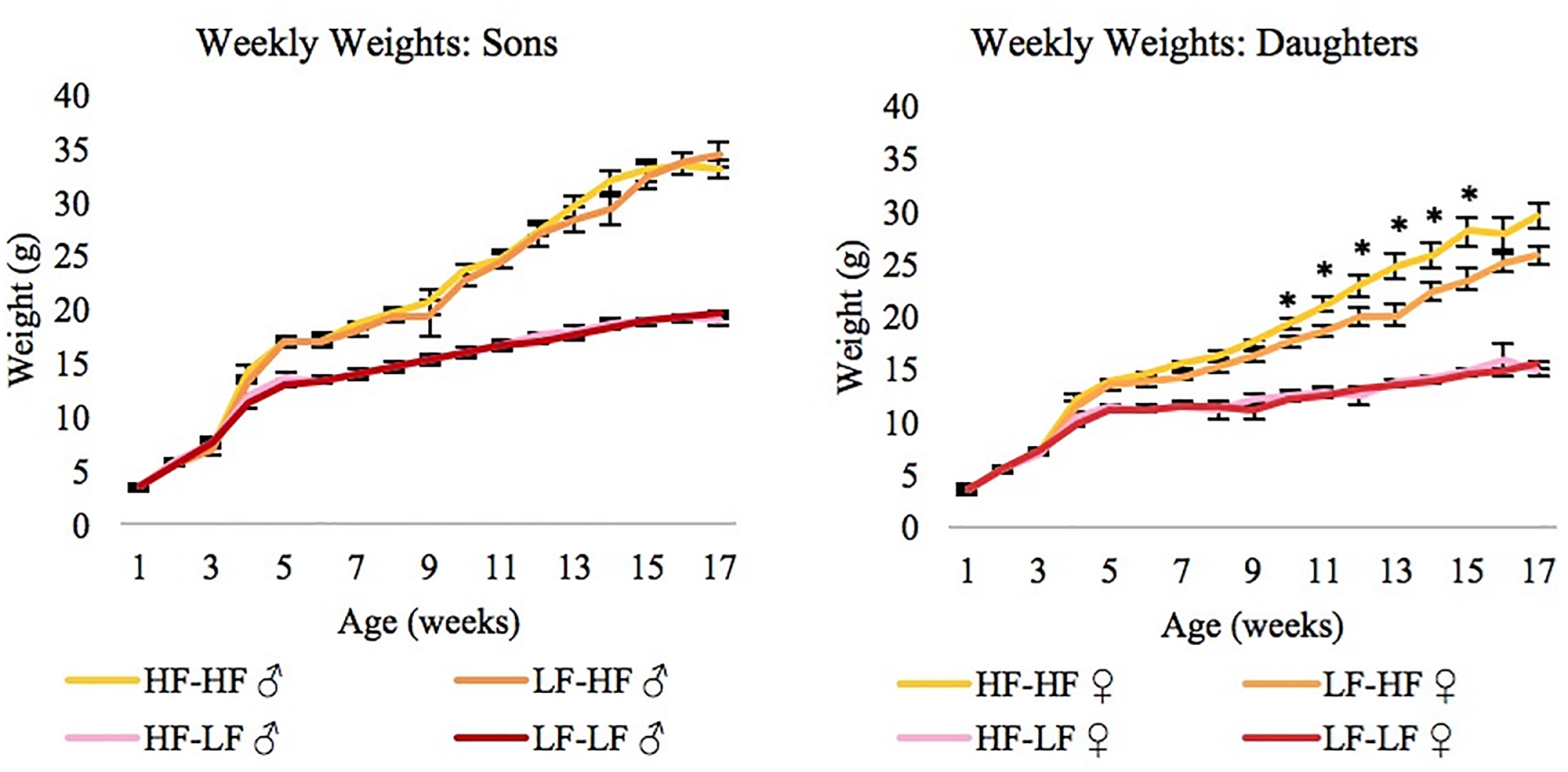
Weekly weights of offspring (± SE). High-fat diet offspring weigh more than low-fat diet offspring by 4 weeks of age (1 week after being weaned onto the diet) (♀ p = 1.66 x 10^−10^, ♂ p = 9.10 x 10^−13^). Maternal diet does not affect body weight in the sons, but it does in the daughters. The ANOVA indicated that maternal diet had a significant effect on weight in daughters starting at week 9 (p = 0.041). Asterisks (*) indicate where pairwise comparisons showed significant p values < 0.05. As adults, high-fat daughters weigh even more if their mothers were also on a high-fat diet. HF = High-fat diet, LF = Low-fat diet, N = 10 per group, the first diet listed (before the hyphen) indicates the maternal diet, and the second diet (after the hyphen) indicates offspring diet.

**Fig 2:**
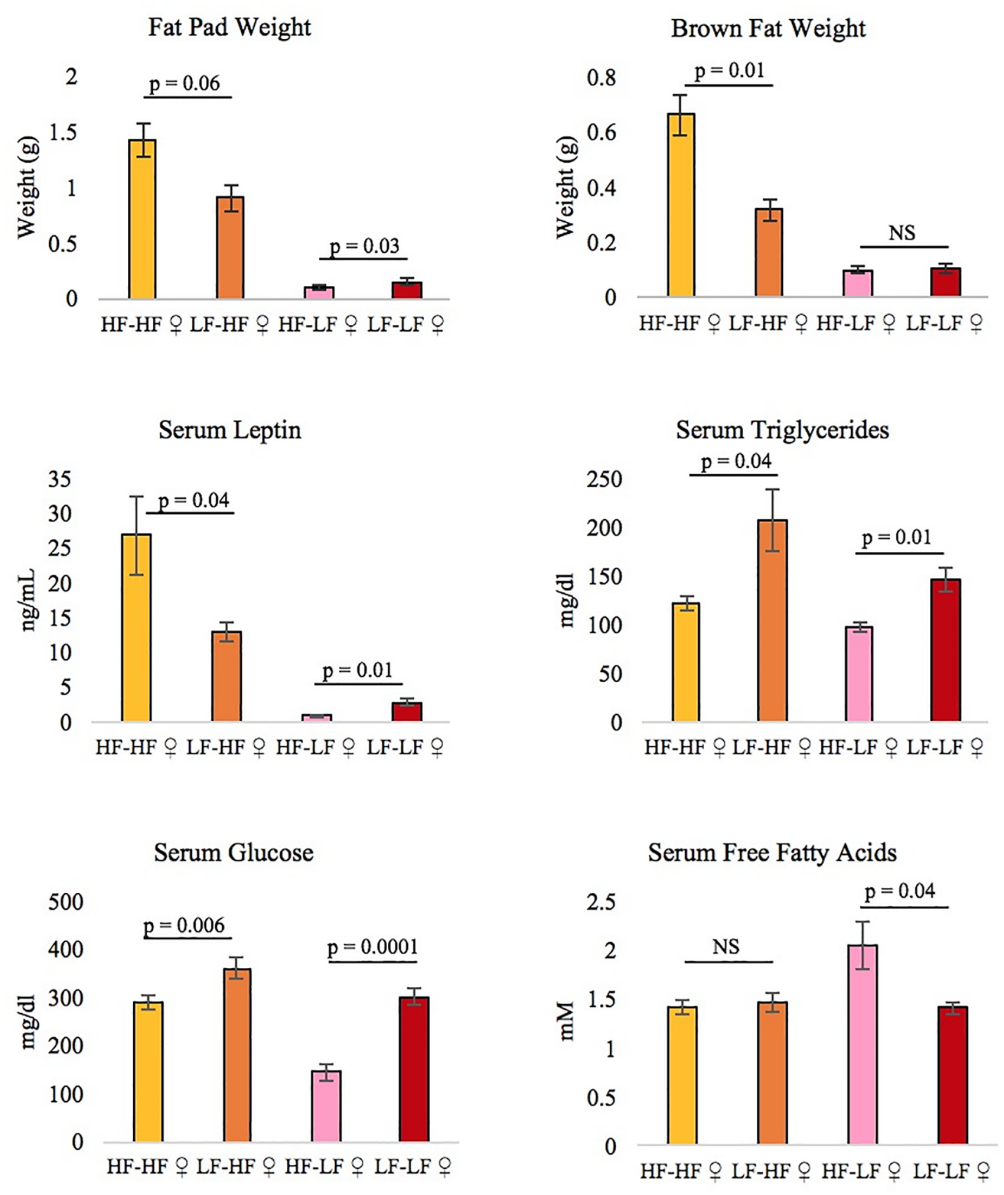
Traits in female offspring affected by maternal diet (± SE). Each bar represents the average of 10 female offspring. ANOVA revealed that maternal diet had a significant effect on brown fat weight (p = 0.001), reproductive fat pad weight (p = 0.018), and serum levels of leptin (p = 0.050), triglycerides (p = 3 x 10^−4^), glucose (p = 2 x 10^−7^), and free fatty acids (p = 0.045). P-values for pairwise comparisons are shown on the graphs. HF = High-fat diet, LF = Low-fat diet, N = 10 per group, the first diet listed (before the hyphen) indicates the maternal diet, and the second diet (after the hyphen) indicates offspring diet.

**Fig 3:**
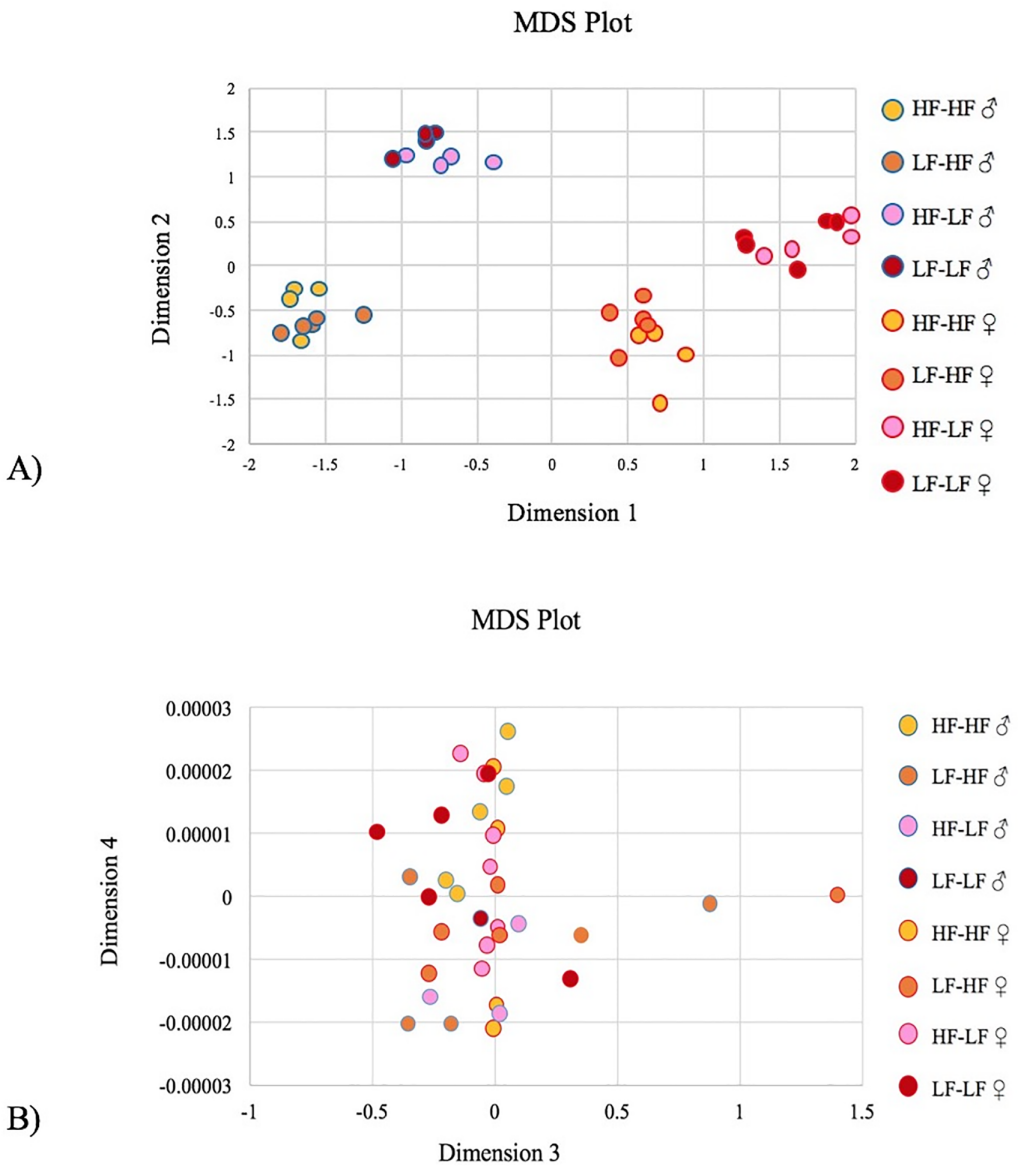
The multidimensional scaling plot. (A) Gene expression libraries clustered by sex (dimension 1) and offspring diet (dimension 2), but not maternal diet. (B) There were no discernable patterns in dimensions 3 or 4. HF = High-fat diet, and LF = Low-fat diet, N = 10 per group.

The direct effect of diet was more extensive than the maternal effect of diet. Maternal diet affected the obesity traits of the daughters, but not the sons. HF-fed daughters weighed more in adulthood (week 9, p = 0.041) (Fig 1), had higher serum levels of leptin (p = 0.05) (Fig 2), consumed more food at 14 weeks of age (p = 0.032) (Fig 3B), and had even more brown fat (p = 0.001) if they had HF mothers rather than LF mothers (Fig 2). LF-fed daughters of HF mothers had the highest levels of free fatty acids in their serum (p = 0.04), and a maternal HF diet actually lowered the triglyceride (p = 3.0 x 10^−4^) and glucose levels (p = 2.0 x 10^−7^) in daughters on either diet (Fig 2). A maternal HF diet significantly affected reproductive fat pad weight in the daughters (p = 0.018), increasing fat pad weight in the HF daughters and having the reverse effect in the LF daughters (Fig 2).

The effect of maternal diet depended on the sex of the offspring. When males and females were analyzed together, there was a significant maternal-diet-by-offspring-diet-interaction for the necropsy traits, but not for the weekly weights or diabetes-related traits. When the sexes were analyzed separately, there was no maternal-diet-by-offspring-diet interaction in the sons, however in the daughters there was a significant interaction effect for the weekly weights and necropsy traits. On a univariate level, the interaction significantly affected: the serum levels of leptin, insulin, free fatty acids, and glucose; weight after 10 weeks of age; and the weights of the liver, reproductive fat pad, kidneys, and brown fat. The genes with differential expression were almost entirely different in the sons and daughters (only 6 genes overlapped). Although only the daughters showed phenotypic differences in the obesity and diabetes traits due to maternal diet, gene expression and methylation were affected in all offspring.

### Gene expression

On the multidimensional scaling (MDS) plot, gene expression libraries clustered by sex (dimension 1, 70% of the variance) and offspring diet (dimension 2, 21% of the variance) (Fig 3A), without any discernable patterns in dimensions 3 or 4 (7% of the variance) (Fig 3B).

**Fig 4:**
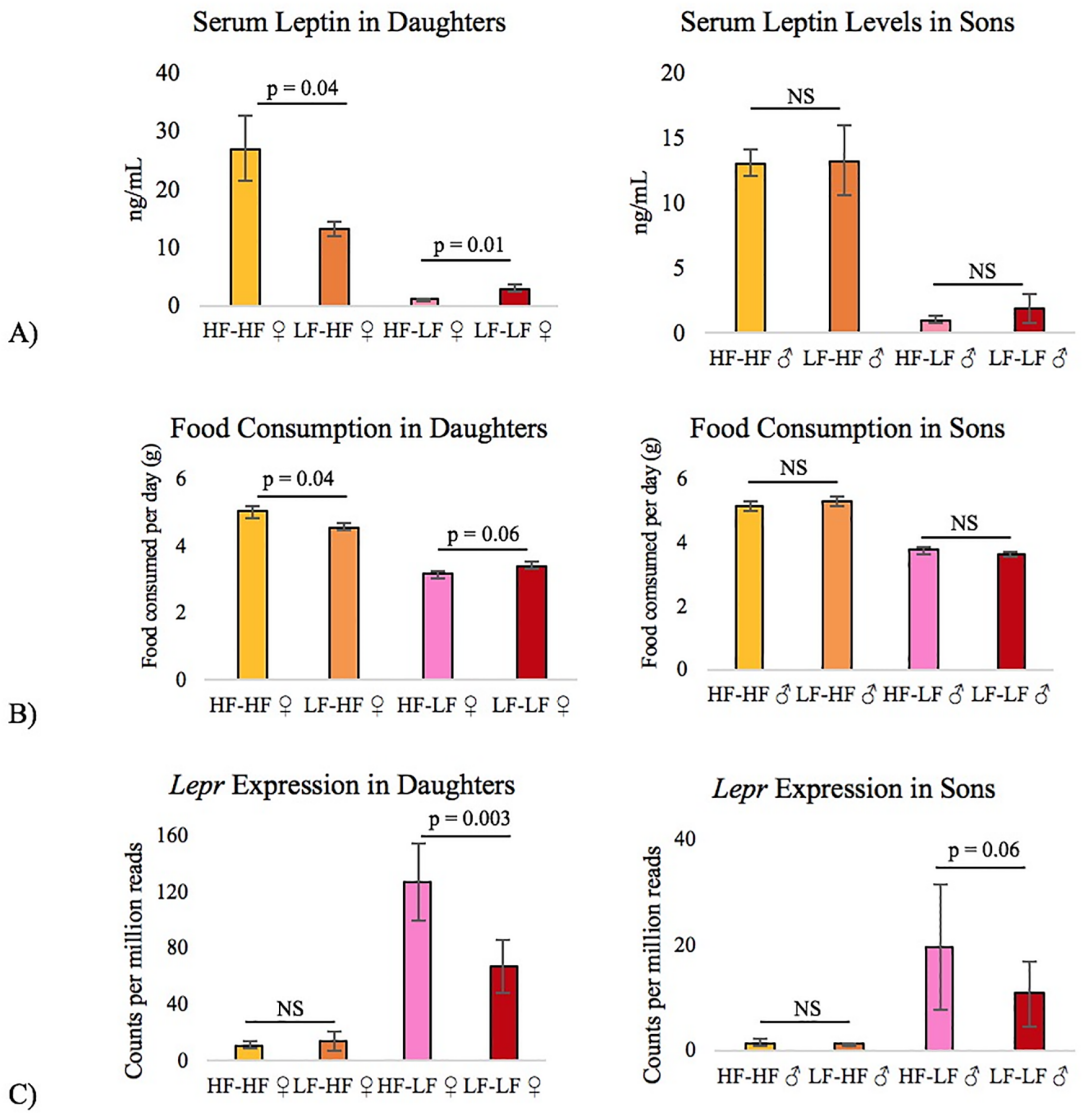
Effect of diet on leptin regulation. (A) High-fat mice had more serum leptin (♀ p = 5.27 x 10^−6^, ♂ p = 2.38 x 10^−8^), however (B) high-fat mice still consumed more food as adults (♀ p = 2.66 x 10^−15^, ♂ p = 1.67 x 10^−15^). (C) They also had reduced expression of the leptin receptor (♀ p = 4.53 x 10^−12^, ♂ p = 8.64 x 10^−8^) in the liver, although it is unknown if there was a similar reduction in the hypothalamus. Maternal high-fat diet further increased the serum leptin levels and food consumption in high-fat-fed daughters, but did not have this effect in sons (see p-values of pairwise comparisons on the graphs). HF = High-fat diet, LF = Low-fat diet, N = 10 per group, the first diet listed (before the hyphen) indicates the maternal diet, and the second diet (after the hyphen) indicates offspring diet.

Offspring diet was associated with expression differences in 3,908 genes in the liver. Of note is the considerable downregulation of the leptin receptor (*Lepr*) gene in HF mice (p = 1.54 x 10^−10^). HF offspring had far more leptin in their serum. HF daughters had over 5 times more than LF daughters (p = 5.27 x 10^−6^), and HF sons had over 3 times more than LF sons (p = 2.38 x 10^−8^) (Fig 4A). Despite having more of this satiety hormone, HF offspring actually ate more than LF offspring at 14 weeks of age (♀ p = 2.66 x 10^−15^, ♂ p = 1.67 x 10^−15^) (Fig 4B). Interestingly, there was a substantial reduction in the liver expression of *Lepr*, which was 8 times lower in the HF daughters than the LF daughters (p = 4.53 x 10^−12^) and 12 times lower in the HF sons than the LF sons (p = 8.64 x 10^−8^) (Fig 4C).

Maternal diet significantly altered the expression of dozens of genes. When comparing offspring of the same sex and diet but with different maternal diets, we found that maternal diet was associated with expression differences in 46 genes in the HF-fed daughters’ livers, 45 genes in the HF-fed daughters’ hearts, 70 genes in the LF-fed daughters’ livers, 22 genes in the HF-fed sons’ livers, and 434 genes in the LF-fed son’s livers (Tables F-J in S1 File). The GAGE pathway analysis revealed many signaling and metabolism pathways that were downregulated in offspring of HF mothers (Table K in S1 File). Maternal diet was associated with the downregulation of more pathways in the livers of the LF-fed offspring (28 downregulated pathways in the LF daughters and 146 in the LF sons) than in the HF-fed offspring (4 in the HF daughters’ livers and 51 in the HF sons’ livers). A maternal HF diet was linked to the downregulation of the ribosome, spliceosome, oxidative phosphorylation, and RNA transport pathways in the livers of all four of the offspring diet-sex group comparisons. The GO Biological Processes associated with maternal diet showed a similar trend of more being dysregulated in the LF-fed offspring, with 284 processes altered in the LF-fed daughters and 2,660 in the LF-fed sons, compared to 31 in the HF daughters and 625 in the HF sons.

The top GO terms associated with maternal diet in the liver involved: RNA splicing and processing, non-coding RNA processing, immune response, and protein catabolic processes (Table 2). In the HF-fed daughters’ heart tissue, maternal HF diet was associated with downregulated biosynthetic and metabolic pathways (Table 2). An offspring HF diet significantly changed the regulation of 28 KEGG disease pathways in the liver, including downregulating the non-alcoholic fatty liver disease (NAFLD) (Fig A in S1 File), Alzheimer’s disease (Fig B in S1 File), Parkinson’s disease, and Huntington’s disease pathways. A maternal HF diet further downregulated these four pathways in each of the offspring diet-sex group comparisons.

**Table 2:** Top 10 significant GO biological processes affected by maternal diet. A negative logFC value indicates that the process was downregulated in mice with high-fat-fed mothers.

Comparison	GO Term	Mean LogFC	FDR
HF-HF ♀ vs. LF-HF ♀	GO:0022613 ribonucleoprotein complex biogenesis	-6.83	7.68E-08
	GO:0016071 mRNA metabolic process	-6.57	8.43E-08
	GO:0008380 RNA splicing	-6.62	8.43E-08
	GO:0034470 ncRNA processing	-6.47	2.19E-07
	GO:0006397 mRNA processing	-6.13	8.27E-07
	GO:0034660 ncRNA metabolic process	-6.11	8.50E-07
	GO:0042254 ribosome biogenesis	-5.58	2.28E-05
	GO:0006412 translation	-5.37	3.55E-05
	GO:0000377 RNA splicing, via transesterification reactions with bulged adenosine as nucleophile	-5.27	6.72E-05
	GO:0000398 mRNA splicing, via spliceosome	-5.27	6.72E-05
HF-HF ♂ vs. LF-HF ♂	GO:0045087 innate immune response	-7.39	9.16E-10
	GO:0050776 regulation of immune response	-6.98	7.56E-09
	GO:0022613 ribonucleoprotein complex biogenesis	-6.88	1.76E-08
	GO:0098542 defense response to other organism	-6.72	1.85E-08
	GO:0043043 peptide biosynthetic process	-6.72	1.85E-08
	GO:0043900 regulation of multi-organism process	-6.67	2.17E-08
	GO:0034660 ncRNA metabolic process	-6.69	2.17E-08
	GO:0006412 translation	-6.57	2.97E-08
	GO:0050778 positive regulation of immune response	-6.57	2.97E-08
	GO:0034470 ncRNA processing	-6.59	4.22E-08
HF-LF ♀ vs. LF-LF ♀	GO:0007067 mitotic nuclear division	-8.65	9.02E-14
	GO:0000280 nuclear division	-7.93	7.91E-12
	GO:0043632 modification-dependent macromolecule catabolic process	-6.90	8.47E-09
	GO:0019941 modification-dependent protein catabolic process	-6.78	1.48E-08
	GO:0006511 ubiquitin-dependent protein catabolic process	-6.72	1.76E-08
	GO:0006281 DNA repair	-6.69	1.90E-08
	GO:0007059 chromosome segregation	-6.73	1.97E-08
	GO:0034660 ncRNA metabolic process	-5.72	5.72E-06
	GO:0010498 proteasomal protein catabolic process	-5.66	5.95E-06
	GO:0034470 ncRNA processing	-5.72	5.95E-06
HF-LF ♂ vs. LF-LF ♂	GO:0006281 DNA repair	-11.77	1.80E-25
	GO:0007067 mitotic nuclear division	-11.62	2.50E-25
	GO:0051345 positive regulation of hydrolase activity	-11.19	7.00E-24
	GO:0000280 nuclear division	-10.62	4.22E-22
	GO:0043632 modification-dependent macromolecule catabolic process	-10.66	6.32E-22
	GO:0019941 modification-dependent protein catabolic process	-10.55	1.46E-21
	GO:0006511 ubiquitin-dependent protein catabolic process	-10.46	3.04E-21
	GO:0050776 regulation of immune response	-10.16	1.64E-20
	GO:0031329 regulation of cellular catabolic process	-10.18	2.17E-20
	GO:0034109 homotypic cell-cell adhesion	-9.95	1.24E-19
HF-HF ♀ vs. LF-HF ♀ heart	GO:0006631 fatty acid metabolic process	-6.18	3.11E-06
	GO:0044283 small molecule biosynthetic process	-5.81	1.18E-05
	GO:0016053 organic acid biosynthetic process	-5.61	2.32E-05
	GO:0046394 carboxylic acid biosynthetic process	-5.61	2.32E-05
	GO:0042738 exogenous drug catabolic process	-6.06	3.97E-05
	GO:0006520 cellular amino acid metabolic process	-5.42	3.97E-05
	GO:0042737 drug catabolic process	-5.85	6.30E-05
	GO:0008202 steroid metabolic process	-5.24	7.91E-05
	GO:1901605 alpha-amino acid metabolic process	-5.24	7.91E-05
	GO:0071466 cellular response to xenobiotic stimulus	-5.40	8.12E-05

The weighted gene co-expression network analysis (WGCNA) provided more insight into the relationship of the gene expression and obesity phenotypes (Table L in S1 File). The phenogram built from clustering the expression libraries had cophenetic correlations all above 0.85, indicating that it closely represented the relationships between the libraries. Setting the minimum module size to 40 genes yielded 29 modules of co-expressed genes, ranging from 34 to 1,785 genes per module. Four of these modules were significantly associated with the diabetes-related traits (week 15 and week 16 weight, glucose tolerance, insulin tolerance, serum glucose and insulin levels, and food consumption) (Fig C in S1 File). The black module was negatively correlated with the diabetes traits and contained 637 genes, which were significantly enriched for immune system function. The yellow module was also negatively correlated with the diabetes traits, and it contained 932 genes that were enriched for terms involving oxidation reduction and arachidonic acid (which is an inflammatory intermediate and a vasodilator). The magenta module was positively correlated with the diabetes traits and contained 312 genes that were enriched for mitochondrial and ribosomal processes, suggesting that the genes in this module were involved in regulating the diet-induced changes in metabolism and gene expression. The turquoise module had 1,785 genes and was positively correlated with the diabetes traits, with enrichment for respiratory chain and mitochondrial processes.

**Fig 5:**
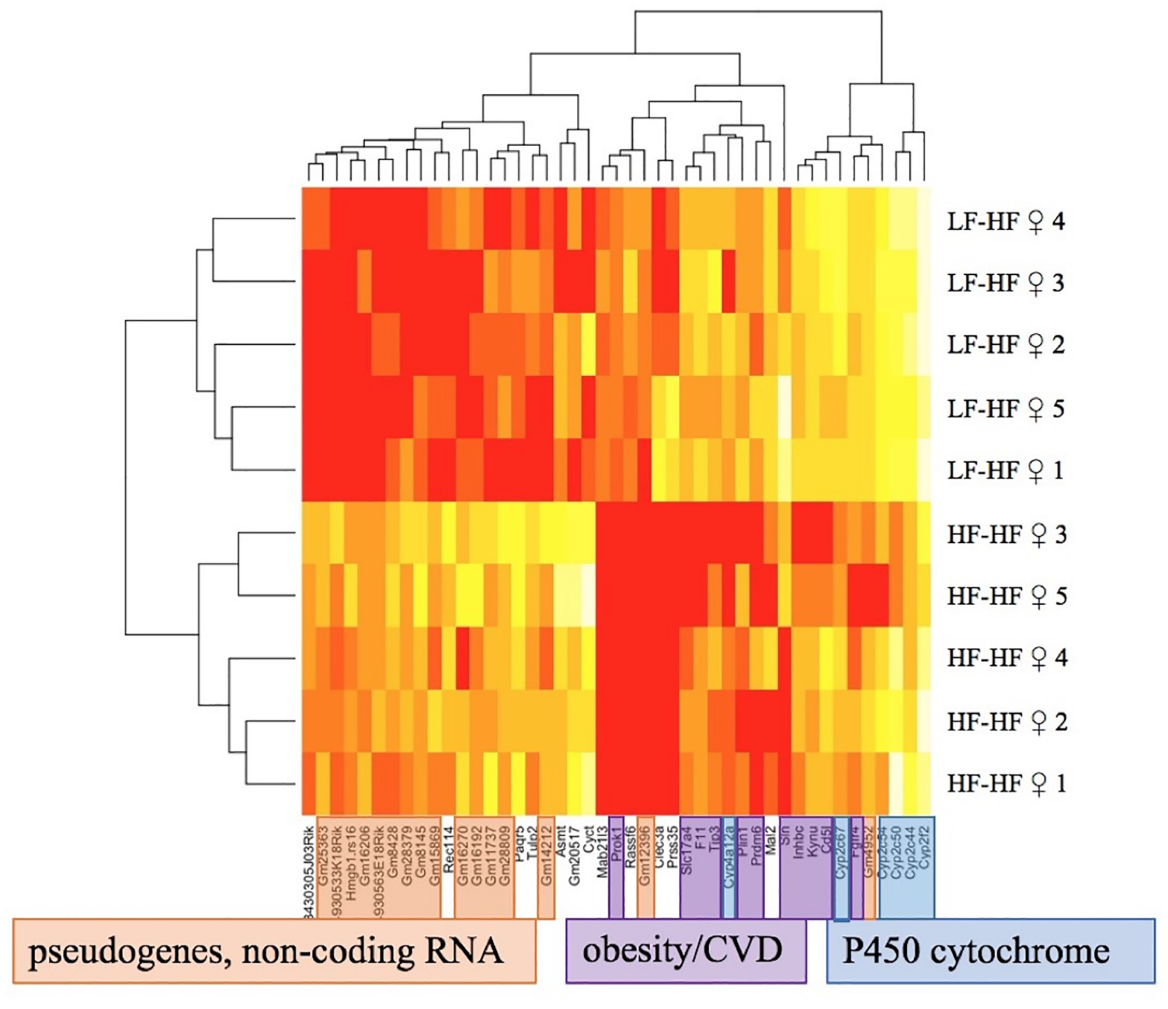
Clustering diagram of the heart gene expression libraries in the high-fat diet daughters. The 21 upregulated genes due to a maternal high-fat diet are mostly pseudogenes and non-coding RNAs. The 24 downregulated genes due to a maternal high-fat diet are primarily p450 cytochromes and genes already known to be involved in obesity and cardiovascular diseases. HF = High-fat diet, LF = Low-fat diet, the first diet listed is maternal diet and the second diet is the offspring diet, N = 10 per group.

A maternal HF diet was linked to disrupted gene expression not only in the offspring’s livers, but in their hearts as well. Comparing the HF-fed daughters of HF mothers with HF-fed daughters of LF mothers revealed 45 differentially expressed heart genes associated with maternal diet (Table G in S1 File). The gene expression libraries clustered by maternal diet, and the cophenetic correlations of the phenogram were all above 0.93 (Fig 5). The 21 upregulated genes due to maternal HF diet were primarily pseudogenes and non-coding RNAs. Most of the 24 downregulated genes were ones previously reported to be involved in obesity and cardiovascular diseases, in addition to 6 cytochrome P450 genes.

### Methylation

There were tens of thousands of differentially methylated regions (DMRs) associated with maternal diet that fell below a q-value cutoff of 0.05 (0.7–1.1% of the 5.3 million 500-bp windows in the genome) (Table 3). A q-value cutoff of 0.01 encompassed less than 0.2% of the windows, and highlighted several thousand regions associated with maternal diet (Tables A-E in S2 File). Less than 300 windows fell below the q-value cutoff of 0.001 in the HF-fed offspring, whereas 10 times as many windows did in the LF-fed offspring. A higher percentage of DMRs were found on the X-chromosome for between-sex comparisons (1.9–3.0%) than within-sex comparisons (0.1–0.5%). The daughters had a greater proportion of DMRs on the X-chromosome (0.2% and 0.5%) associated with maternal diet than the sons (0.1%).

**Table 3:** Distribution of differentially methylated regions (DMRs). Thousands of DMRs in the liver were associated with maternal diet. Low-fat offspring had more DMRs than high-fat offspring. There were more than twice as many DMRs on the X-chromosome when comparing between sexes than within sexes. HF = High-fat diet, LF = Low-fat diet, the first diet listed is maternal diet and the second diet is the offspring diet, N = 2 libraries of 5 mice each (10 mice total) per maternal-diet-offspring-diet-sex group.

Comparison	Group	p<0.05	p<0.01	p<0.001	DMRs in X (%)
**Different diets**	HF-HF ♀ vs. LF-HF ♀	34,844	1,701	232	79 (0.2%)
	HF-LF ♀ vs. LF-LF ♀	55,014	9,550	2,566	284 (0.5%)
	HF-HF ♂ vs. LF-HF ♂	40,437	2,262	258	28 (0.1%)
	HF-LF ♂ vs. LF-LF ♂	57,374	8,737	1,505	75 (0.1%)
**Different sexes**	HF-HF ♀ vs. HF-HF ♂	41,340	5,031	1,679	1,219 (3.0%)
	HF-LF ♀ vs. HF-LF ♂	66,447	14,571	4,533	1,278 (1.9%)
	LF-HF ♀ vs. LF-HF ♂	40,766	5,610	1,728	1,074 (2.6%)
	LF-LF ♀ vs. LF-LF ♂	54,304	10,201	3,048	1,185 (2.2%)

An offspring HF diet was associated with an overall reduction in variation in methylation. When comparing offspring that had the same diet but different maternal diets, the LF-fed offspring had more maternal diet DMRs than the HF-fed offspring. For instance, when HF-fed daughters of HF mothers were compared to HF-fed daughters of LF mothers, they had 1,701 DMRs (q < 0.01) due to maternal diet. However, when LF-fed daughters with different maternal diets were compared, they had 9,550 DMRs—over five times more than the HF-fed daughters had. The same trend was seen in the sons, where the HF-fed sons had 2,262 DMRs due to maternal diet and the LF-fed sons had 8,737 DMRs. The pattern continued when comparing mice of different sexes who had the same maternal diet and offspring diet. HF-fed sons and daughters of HF mothers had 5,031 DMRs, and similarly HF-fed sons and daughters of LF mothers had 5,610 DMRs. This was 2–3 times fewer than the LF-fed offspring; when LF-fed sons and daughters of HF mothers were compared they had 14,571 DMRs, and LF-fed sons and daughters of LF mothers had 10,201 DMRs.

In the HF offspring, over 7,300 genes (36% of genes) in the liver contained at least one DMR associated with maternal diet (q < 0.05) between its outermost transcription start and stop sites, whereas LF offspring had 9,300 genes (46% of genes) with a DMR (Table 4).

**Table 4:** Distribution of DMRs in genes. The number of genes in the mouse liver with at least one differentially methylated region (DMR) associated with maternal diet within the gene body, more than one DMR in the gene body, and at least one DMR in the promoter region (within 2 kb upstream of the transcription start site (p < 0.05). HF = High-fat diet, LF = Low-fat diet, first diet listed is maternal diet and second diet (after the hyphen) is the offspring diet.

	Genes with ≥1 DMR in gene body	Genes with >1 DMR in gene body	Genes with ≥1 DMR in promoter
**HF-HF ♀ vs. LF-HF ♀**	7,367 (36.1%)	3,554 (17.4%)	1,878 (9.2%)
**HF-LF ♀ vs. LF-LF ♀**	9,358 (45.8%)	5,347 (26.2%)	2,724 (13.4%)
**HF-HF ♂ vs. LF-HF ♂**	7,980 (39.1%)	4,031 (19.8%)	2,213 (10.8%)
**HF-LF ♂ vs. LF-LF ♂**	9,369 (45.9%)	5,260 (25.8%)	3,254 (16.0%)

About 14% of the DMRs associated with maternal diet fell within promoters, 23% within exons, 35% in intergenic regions, and the rest in introns (Table 5). When Ensembl regulatory elements were included in the classification scheme, it became clear that the DMRs were disproportionately found in regulatory regions. Between 10 and 20% of DMRs overlapped enhancers (whereas only 3.5% of the 5.3 million windows genome-wide overlapped enhancers). Similarly, 5–10% of DMRs overlapped CTCF Binding sites (compared to 1.7% of windows genome-wide), 0.6–1.3% overlapped other transcription factor binding sites (compared to 0.3% of windows genome-wide), and 31–50% overlapped promoter flanking regions (compared to 8.1% of windows genome-wide).

**Table 5:** Distribution of significant differentially methylated regions (DMRs) (p < 0.01) across the genome associated with maternal diet. Values indicate the number of 500 base-pair windows overlapping each genomic region, with the percent of the total significant DMRs overlapping these regions in parentheses. As a comparison, the percentage of windows across the whole genome that overlap these genomic regions is listed, demonstrating how overrepresented the DMRs are in regulatory regions.

Region	High Fat Daughters	High Fat Sons	Low Fat Daughters	Low Fat Sons	Whole Genome
Enhancer	180 (10.6%)	259 (20.3%)	1,038 (10.9%)	1,024 (11.7%)	3.5%
CTCF Binding Site	102 (6.0%)	129 (10.1%)	450 (4.7%)	614 (7.0%)	1.7%
TF binding site	16 (0.9%)	16 (1.3%)	62 (0.6%)	93 (1.1%)	0.3%
Promoter Flanking Region	549 (32.3%)	637 (49.9%)	3,625 (38.0%)	2,729 (31.2%)	8.1%
Promoter	251 (14.7%)	311 (13.8%)	1,366 (14.3%)	1,393 (15.9%)	4.5%
Exon	395 (23.2%)	528 (23.4%)	2,651 (27.8%)	1,958 (22.4%)	7.5%
Intergenic	616 (36.2%)	807 (35.7%)	2,870 (30.0%)	3,037 (34.8%)	58.6%

In HF-fed daughters, 23 (0.067%) of the maternal diet DMRs (q < 0.05) were located in differentially expressed genes, although 45 DMRs (0.13%) would have been expected to fall in differentially expressed genes due to chance. In the LF-fed daughters, 80 (0.15% of) DMRs fell within differentially expressed genes, whereas 97 (0.18%) would have been expected due to chance. Thus, in the daughters, DMRs associated with maternal diet fell within differentially expressed genes less often than expected due to chance (χ^2^, p = 0.0002). The opposite was true in the sons. The HF-fed sons had 22 (0.05%) of their DMRs in differentially expressed genes, while 17 (0.04%) were expected to be there by chance. The LF-fed sons had 297 (0.52%) of their DMRs in differentially expressed genes, while 208 (0.36%) would have been expected to be there by chance. In the sons, DMRs fell within differentially expressed genes more often than expected (χ^2^, p = 3.2 x 10^−10^). One-quarter to one-third of differentially expressed genes contained a DMR associated with maternal diet (23.9% of differentially expressed genes in the HF-fed daughters, 37% in the LF-fed daughters, 36.4% in the HF-fed sons, and 23.2% in the LF-fed sons).

## Discussion

Independent of maternal diet, an offspring high-fat (HF) diet induced a vast array of changes in the SM/J mice: it increased body and organ weights; reduced sensitivity to insulin; increased the serum levels of leptin, insulin, triglycerides, glucose, and free fatty acids; increased the lengths and weights of the long bones; and changed the expression of 3,908 genes in the liver. HF-fed mice had 8 times lower expression of the leptin receptor (*Lepr*) gene than LF-fed mice. Leptin signaling in the liver is important for lipid metabolism and is impaired in obesity [42, 43], and SNPs in *Lepr* in humans are associated with NAFLD [44]. It would be interesting in the future to determine if there is a similar reduction of *Lepr* in the hypothalamus, as such a phenomenon could explain why the HF-fed mice consumed more food despite having 3–5 times more leptin in their serum.

An HF diet had a larger effect on the males than the females in terms of inducing more gene expression differences (1,662 differentially expressed genes in males and 1,224 in females), causing an impaired response to intraperitoneal glucose and insulin tolerance testing, and even further increasing the weights of the liver and kidneys. Meanwhile, an HF diet had a larger effect on the reproductive fat pad weight in the females. There were 1,062 genes differentially expressed in the males that were not differentially expressed in the females, and 602 genes differentially expressed in the females but not the males (637 genes were differentially expressed in both sexes). These sex differences underscore the importance of including both males and females in diet studies.

The weighted gene co-expression network analysis (WGCNA) revealed several modules of highly co-expressed genes that were directly linked to the diabetes-related traits in the offspring. The two modules that were negatively associated with the diabetes-related traits contained genes that were significantly enriched for immune system function, oxidation reduction, and arachidonic acid metabolism. The two modules that were positively correlated with the diabetes traits were enriched for mitochondrial, respiratory, and ribosomal processes. Overall, the modules tended to involve the immune system and mitochondria, indicating that the disruption of these processes was linked to the development of the diabetes traits we measured.

A maternal HF diet was associated with expression differences in dozens of genes in the offspring’s livers, methylation differences in thousands of genes (36–46% of genes in the liver had at least one differentially methylated region), and in the daughters it affected the adult body weights, organ weights, and serum biomarkers. Compared to HF-fed daughters of LF mothers, HF-fed daughters of HF mothers weighed more, had more brown fat, and had higher levels of leptin in their serum in adulthood. LF-fed daughters were also affected by maternal diet. Compared to LF-fed daughters of LF mothers, LF-fed daughters of HF mothers had significantly higher levels of free fatty acids in their serum and lower reproductive fat pad weights. Interestingly, a maternal HF diet lowered the triglyceride and glucose levels in the daughters on either diet. This is similar to the finding of Ashino *et al*. [45] that adult male Swiss mice had higher levels of triglycerides in the liver but lower levels in the serum due to maternal HF diet, possibly due to inadequate export of triglycerides from the liver.

Both an offspring HF diet and a maternal HF diet downregulated the non-alcoholic fatty liver disease (NAFLD) pathway. The parts of the pathway that were especially downregulated in offspring of HF mothers were the mitochondrial respiratory chain complexes. Reduced activity of the respiratory chain complexes are one of the mitochondrial abnormalities associated with NAFLD, in addition to impaired mitochondrial β-oxidation, increased mitochondrial size, and decreased mitochondrial number [46, 47]. These results support findings in other mouse studies that maternal obesity can program NALFD in offspring. For instance, C57BL/6J mice fed an obesogenic diet had more severe liver injury if their mothers had also been on obesogenic diets, and this appeared to be mediated by immune dysfunction [48] and disrupted circadian rhythms [49]. This was accompanied by differential expression and promoter hypermethylation of the biological clock genes *Bmal-1* and *Per2* in the liver [49]. Our LF-fed sons had a maternal diet DMR in an intron of the *Per2* gene, but the expression was not altered.

It has been demonstrated that NAFLD is associated with Alzheimer’s disease in mice [50], and thus it is not surprising that the Alzheimer’s disease pathway was also downregulated by both an offspring and a maternal HF diet in this study. The parts of the pathway particularly downregulated in offspring of HF mothers were the mitochondrial respiratory chain complexes and the SERCA Ca(2+)-ATPase intracellular pumps. Disrupted SERCA activity and calcium homeostasis can lead to Alzheimer’s disease [51]. There was also a modest change in the amyloid precursor protein (*App*) gene. APP is cleaved to produce amyloid beta (Aβ) peptides, which can form plaques in the brain in Alzheimer’s disease. Aβ is not only produced in the brain, but also in the liver, and it can be transported into the brain by low-density lipoprotein receptors [52, 53]. It has been shown that treating mice with a drug that cannot cross the blood-brain barrier lowered Aβ in both the blood and the brain [52], indicating that Alzheimer’s disease may start with a peripheral excess of Aβ that then enters the brain. In our study, daughters exposed to a maternal HF diet had a slight overexpression of *App* (1.21 times higher in LF-fed daughters, p = 0.015, and 1.15 times higher in HF-fed daughters, p = 0.062). It would be interesting in the future to determine if this increase in *App* associated with a maternal high-fat diet raises levels of Aβ in the blood, predisposing the offspring to developing amyloid plaques.

Reduced levels of the important antioxidant glutathione have been implicated as a cause for the oxidative stress in Alzheimer’s disease [54], and it has been suggested that therapeutically increasing glutathione levels could treat the disease [55]. A maternal HF diet significantly downregulated glutathione metabolism in our mice, which may have been an early indicator of a reduced ability to respond to oxidative stress and could have predisposed the offspring to neurological impairment—further research is needed to investigate this possibility. It is known that obesity raises the risk of Alzheimer’s disease [56, 57], but the results in our study suggest that a maternal HF diet may also elevate that risk.

In all offspring, a maternal HF diet downregulated the ribosome, spliceosome, oxidative phosphorylation, and RNA transport pathways, indicating that maternal diet has an extensive effect on the offspring transcriptome. Other studies have found that a high maternal BMI downregulates genes involved in mitochondrial and lipid metabolism in the cord blood of infants [58], maternal obesity in sheep downregulates AMPK signaling pathways in offspring skeletal muscle [59], and maternal obesity downregulates mitochondrial pathways in the skeletal muscle of male rat offspring, including the oxidative-phosphorylation and electron transport pathways [60]. In our LF-fed offspring, a maternal HF diet also downregulated mitochondrial pathways such as oxidative-phosphorylation, in addition to several key metabolic pathways.

In addition to its effects in the liver, a maternal HF diet had an even larger effect on the methylation and gene expression in the hearts of HF-fed daughters. The set of genes differentially expressed in the heart and the liver did not overlap at all, underscoring the importance of investigating multiple tissues to understand the full scope of the effects of a maternal high-fat diet. There were 4,103 differentially methylated regions in the heart and 45 differentially expressed genes associated with maternal diet in the daughters, including 6 cytochrome P450 genes. Cytochrome P450 genes are important for homeostasis, and encode enzymes involved in metabolizing endogenous compounds such as fatty acids, steroids, and drugs. A strong link between cytochrome P450 enzymes and heart failure has been reported [61]. Many cytochrome P450 enzymes have been found in the heart, with altered levels during cardiac hypertrophy and heart failure. None of the P450 genes identified in the present study are on Phenopedia’s list of genes associated with cardiovascular diseases, but one (*Cyp2c44*) was identified as protective against pulmonary hypertension in female mice [62]. Due to the role that cytochrome P450 genes play in homeostasis and drug metabolism, the genes we identified (*Cyp4a12a*, *Cyp2c67*, *Cyp2c54*, *Cyp2c50*, *Cyp2c44*, and *Cyp2f2*) should be further investigated in the context of maternal obesity and response to pharmaceutical treatments for metabolic syndrome and heart disease.

More DMRs were found on the X-chromosome in between-sex comparisons than within-sex comparisons, which was to be expected because sex has a substantial effect on X-chromosome methylation [63, 64]. More DMRs were located on the X-chromosome in the daughters than in the sons, which may be a result of X-chromosome inactivation, as this is known to be maintained by DNA methylation [65, 66]. The LF-fed offspring had more disrupted genes and pathways associated with maternal diet than HF-fed offspring. Likewise, the LF-fed offspring had tens of thousands more methylation differences associated with maternal diet than HF-fed offspring, including on the X-chromosome. These trends indicate that the direct effect of an offspring HF diet may be so strong that it dampens the effect that a maternal HF diet has on gene expression and methylation.

In the sons, maternal diet DMRs were found in differentially expressed genes more often than expected by chance, but in the daughters the trend was reversed. These results indicate that—while DNA methylation is linked to differential expression in some genes—overall, DNA methylation within genes is likely not the primary driver of differential expression in response to a maternal HF diet. Instead, the DMRs located outside of the gene bodies are likely to play a larger role. The maternal diet DMRs fell within regulatory regions far more often than expected by chance, with 10–20% overlapping enhancers, 5–10% overlapping CTCF Binding sites, and 31–50% overlapping promoter flanking regions.

Many of the genes that were differentially expressed did not have DMRs (63–77%), and many of the DMRs were located within genes that were not differentially expressed (90%). This is in line with current understanding that most DMRs are not associated with changes in expression [67, 68]. DNA methylation works in concert with histone modifications and other epigenetic factors to control gene expression, and thus is only one piece of the regulatory puzzle. Nevertheless, many genes important to obesity and diabetes have been discovered to exhibit differences in DNA methylation associated with differences in expression [69, 70]. It is critical to identify all such genes in order to understand the relevant methylation changes induced by maternal diet, opening the door to targeted therapeutic treatments in the future.

**Fig 6:**
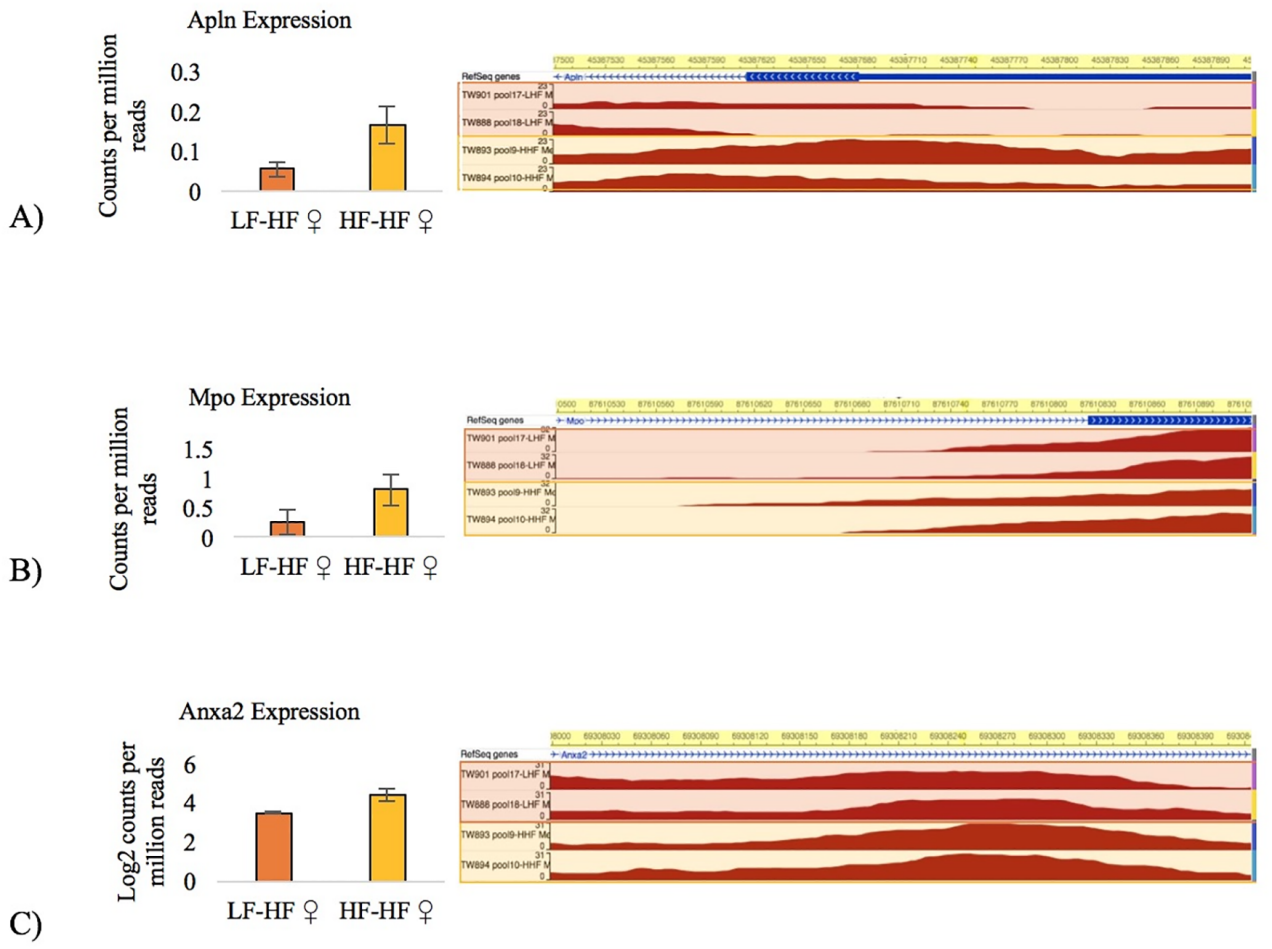
Examples of differentially expressed genes that have differentially methylated regions within them associated with maternal diet. The bar graphs depict gene expression and the WashU Epigenome Browser plots depict the level of methylation as determined by MeDIP-seq. (A) The HF-HF daughters had higher expression of *Apln* (p = 0.02) and higher promoter methylation (q = 0.0060). The first LF-HF/HF-HF library comparison had a MeDIP ratio of 17/48 RPKM and an MRE ratio of 236/232 RPKM; the second LF-HF/HF-HF library comparison had a MeDIP ratio of 19/34 RPKM and an MRE ratio of 245/248 RPKM. (B) The HF-HF daughters had higher expression of *Mpo* (p = 4.6 x 10^−9^) and lower methylation in its eighth exon (q = 0.02). The first LF-HF/HF-HF library comparison had a MeDIP ratio of 43/38 RPKM and an MRE ratio of 30/59 RPKM; the second LF-HF/HF-HF library comparison had a MeDIP ratio of 46/40 RPKM and an MRE ratio of 32/57 RPKM. (C) The HF-HF daughters had higher expression of *Anxa2* (p = 1.94x10^-5^) and higher methylation in its first intron (q = 0.025). The first LF-HF/HF-HF library comparison had a MeDIP ratio of 43/46 RPKM and an MRE ratio of 2/13 RPKM; the second LF-HF/HF-HF library comparison had a MeDIP ratio of 40/46 RPKM and an MRE ratio of 2/10 RPKM. HF = High-fat diet, LF = Low-fat diet, error bars represent ± the standard error, N = 10 per group, the first diet listed is the maternal diet and the second diet listed is the offspring diet.

A handful of genes in this study exhibited both differential methylation and differential expression that are relevant to the obesity traits measured in this study. For instance, compared to HF-fed daughters of LF mothers, HF-fed daughters of HF mothers had increased methylation (q = 0.006) in the promoter of the apelin (*Apln*) gene in the liver, which corresponded to higher expression of the gene (p = 0.02) (Fig 6A). This is a case where increased promoter methylation does not correspond with decreased expression. *Apln* activates signaling pathways involved in angiogenesis, insulin, and cardiovascular function [71]. Altered expression levels of *Apln* in the placenta have been linked to preeclampsia [72, 73], and SNPs in *Apln* are associated with Body Mass Index in Chinese women [74] as well as obesity and insulin resistance in Egyptian women [75]. Although *Apln* expression in the liver is understudied, the *Apln* gene and protein have been found to be over-expressed in human cirrhotic liver tissue—and the expression increased with the progression of the cirrhosis [76]. Thus, the overexpression of *Apln* in HF-fed daughters of HF dams may indicate that liver damage is worsened by a maternal HF diet.

Another gene that had both differential methylation and expression associated with maternal diet in the HF daughters was myeloperoxidase (*Mpo*), a hemoprotein released by white blood cells during inflammation. Its products create oxidative stress, and *Mpo* knockout mice are protected from HF diet-induced weight gain and insulin resistance [77]. It has been suggested that *Mpo* links inflammation, oxidative stress, and cardiovascular disease [78]. *Mpo* activity is higher in the livers of obese patients suffering from nonalcoholic steatohepatitis [79]. In the present study, there was also a direct effect of diet on *Mpo* expression, with HF-fed offspring having higher *Mpo* expression in their livers than LF-fed offspring (p = 4.6 x 10^−9^) (Fig D in S1 File). In the daughters, this was exacerbated by a maternal HF diet. Compared to HF-fed daughters of LF mothers, HF-fed daughters of HF mothers had lower methylation in the eighth exon of the *Mpo* gene (q = 0.02) (Fig 6B). The high *Mpo* expression in the HF-fed offspring increased even further with a maternal HF diet in females, suggesting that they were under more severe oxidative stress.

In addition to increasing oxidative stress, obesity raises the levels of inflammatory cytokines [80, 81]. It is thought that the exposure to inflammatory cytokines in the womb can predispose offspring to metabolic disease [82, 83, 84]. Elevated cytokines are known to activate the signal transducer and activator of transcription 1 (*Stat1*) gene [85]. There was a direct effect of offspring diet on *Stat1* in the present study, with the HF-fed mice having higher *Stat1* expression than the LF-fed mice (1.22 x 10^−11^) (Fig D in S1 File). This is important because higher levels of *Stat1* are associated with liver injury and inflammation [85]. Maternal diet also affected *Stat1* in HF-fed sons, but in a protective way by decreasing *Stat1* expression. Compared to HF-fed sons of LF mothers, HF-fed sons of HF mothers had decreased methylation at a DMR less than 2 kb from the *Stat1* transcription start site, in an Ensembl-defined promoter flanking region (Fig D in S1 File). A maternal HF diet somewhat offset the increase in *Stat1* expression caused by an HF diet in the sons, which illustrates that the gene expression changes caused by maternal obesity are not always the same as those caused by an individual’s obesity.

One advantage to taking a whole-genome approach versus a candidate-gene approach in this study is that it enabled us to identify genes that are not well known to be involved in maternal obesity. For instance, annexin A2 (*Anxa2*) is a phospholipid-binding protein that is over-expressed in some tumors and in the blood of people with osteoporosis [86]. *Anxa2* is also involved in cholesterol uptake in the intestine. Although it is unclear what this gene does in the liver, its expression is known to be higher in the livers of diabetic sand rats [87] and the livers of HF-fed C57BL/6J mice [88]. In the present study, a maternal HF diet was associated with over-expression of *Anxa2* in the HF-fed daughters (p = 1.94x10^-5^), as well as hypermethylation of a DMR in its first intron (q = 0.025) (Fig 6C). Further research is required to understand the implications of *Anxa2* overexpression in response to a maternal HF diet.

In this study, we identified dozens of genes that were differentially expressed due to maternal diet, along with thousands of differentially methylated regions. Many of the differentially expressed genes have been found by previous studies, while others are novel in their involvement in obesity. Limitations of this study include studying only one strain of mouse and investigating only one type of epigenetic marker. In the future, it will be important to incorporate other epigenetic factors such as histone modification into the analysis in order to gain a fuller understanding of the epigenetic changes induced by maternal obesity.

## Methods

### Experimental design

**Fig 7:**
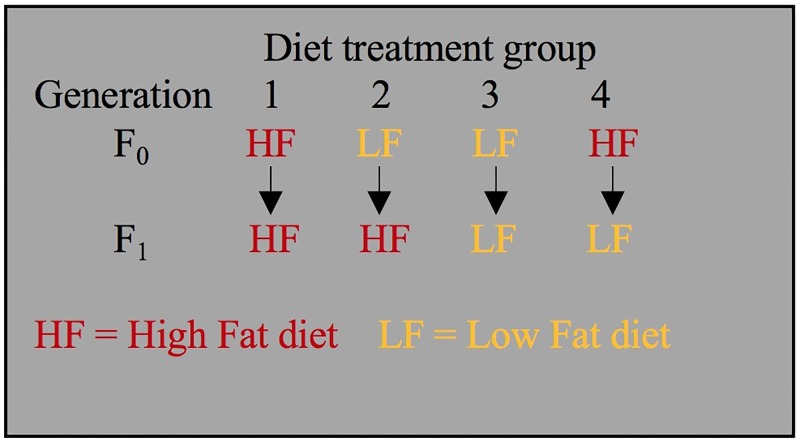
Experimental design.

We investigated the effect that a high-fat (HF) diet had on mice compared to a low-fat (LF) diet, predicting that a maternal HF diet would exacerbate obesity in the offspring. We studied four groups of offspring: HF-fed offspring of HF mothers, HF-fed offspring of LF mothers, LF-fed offspring of HF mothers, and LF-fed offspring of LF mothers (Fig 7). We collected weight and physiological data on the offspring, measured their gene expression using RNA-seq, and measured their DNA methylation using MeDIP-seq and MRE-seq.

### Animal rearing

This research was approved by Loyola University’s Institutional Animal Care and Use Committee protocol (Project #1188). The animals used in this experiment were derived from inbred SM/J mice obtained from The Jackson Laboratory (Bar Harbor, Maine). This strain originated from a selective breeding experiment for small size at 60 days of age [89], and has an extreme obesogenic response to dietary fat [90–92]. The parental generation was born at Loyola University Chicago and was weaned onto a high-fat (HF) diet or a low-fat (LF) diet at 3 weeks of age. The diets were matched to be nearly the same in terms of nutrients and calories, with the primary difference being that the HF diet has three times as much fat. In the LF diet, fat accounted for 15% of the calories (Research Diets D12284), whereas 42% of the calories came from fat in the HF diet (Harlan Teklad diet TD.88137) (Table 6).

**Table 6:** Diet content.

Component	High-fat diet	Low-fat diet
Energy from fat, %	42	15
Casein, g/kg	195	197
Sugars, g/kg	341	307
Corn starch, g/kg	150	313
Cellulose, g/kg	50	30
Corn oil, g/kg	0	58
Hydrogenated coconut oil, g/kg	0	7
Anhydrous milk fat, g/kg	210	0
Cholesterol, g/kg	1.5	0
Kilojoules per gram	18.95	16.99

At 10 weeks of age, 12 HF diet females and 14 LF diet females were mated with LF diet males to create an F_1_ generation. Males were removed from the cage when abdominal palpation revealed the female to be pregnant. To control for the postnatal effect of maternal diet, we cross-fostered all offspring to an LF-fed SM/J nurse within 24 hours of birth. At three weeks of age, half of the offspring of each litter and sex were weaned onto an LF diet and the rest were weaned onto an HF diet to produce 4 diet treatment groups: HF-HF, LF-HF, HF-LF, and LF-LF (where the first diet listed is the maternal diet and the second is the offspring diet). There were 10 male and 10 female offspring in each of the diet treatment groups, and they were housed in same-sex pairs of mice on the same diet. Each cage had a privacy hut (Alt Design), a cotton nestlet (Ancare), a wooden gnawing block (Bio Serve), and food and water provided *ad libitum* in a 12-h light, 12-h dark cycle. Procedures were performed under an approved Institutional Animal Care and Use Committee protocol (Project #1188).

### Obesity phenotypes

The mice were weighed weekly for 17 weeks. They were housed in pairs from weaning at 3 weeks of age until 13 weeks of age, after which they were housed individually. When the mice were 14 weeks old, 20 pellets of food were weighed and placed into their food rack. Food consumption was measured by weighing the food remaining in the cage 24, 48, 96, and 168 hours later. Previous research shows that SM/J mice initially lose weight after being moved to single housing [91], so we measured food consumption after the mice had one week to adjust to being housed singly.

When the mice were 15 weeks old, they underwent an intraperitoneal glucose tolerance test (IPGTT). All IPGTTs were performed at 10:00 AM, after the mice had been fasted for 4 hours. We measured their baseline glucose levels with a glucometer (Ascensia Bayer Breeze 2) using blood from a tail snip, then intraperitoneally injected a 10% glucose solution (0.01 mL/g body weight). We measured the glucose levels again at 30, 60, and 120 minutes after the injection. When the mice were 16 weeks old, they underwent intraperitoneal insulin tolerance testing (IPITT). The IPITT protocol is similar to the IPGTT protocol, except rather than receiving a glucose injection, the mice were injected with a 0.1% insulin solution (0.75 mU/g body weight). If a mouse’s blood glucose levels dropped under 25 mg/dL, it was injected with a 10% glucose solution and was not included in the IPITT results. After the IPGTT and IPITT, the area under the curve (AUC) was calculated using the blood glucose levels at the 4 different time points via the trapezoidal summation method for each mouse.

**Fig 8:**
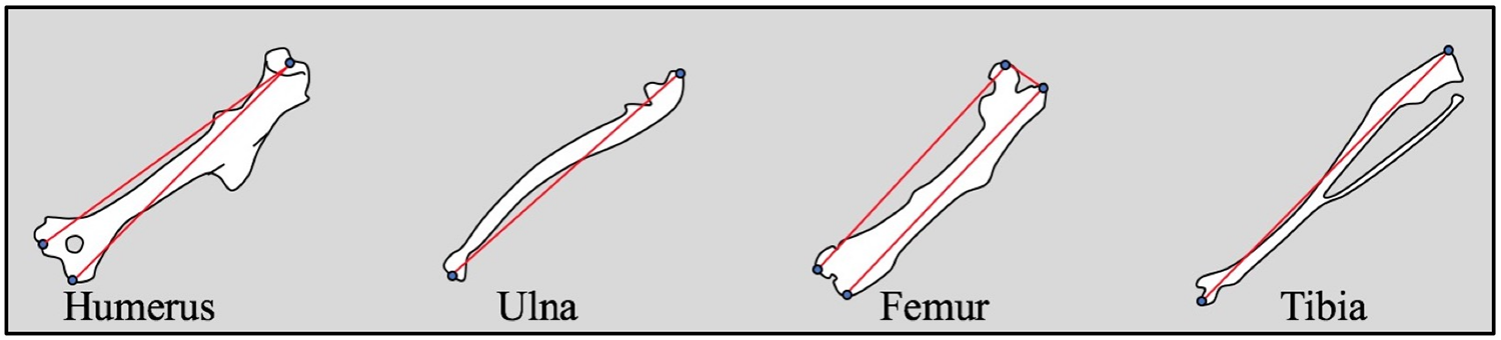
Diagram of the long bone lengths that were measured with calipers. A description of the measurements can be found in Table D in the S1 File.

When the mice were 17 weeks old, they were fasted for four hours and then sacrificed by carbon dioxide asphyxiation between 10:00 am and 2:00 pm. A cardiac puncture was immediately performed to draw blood, and the serum was submitted to Washington University in St. Louis’s Core Laboratory for Clinical Studies to measure leptin and insulin, and to the Diabetes Models Phenotyping Core to measure triglycerides, cholesterol, free fatty acids, and glucose. After the blood draw, we necropsied the mice on ice and recorded the weights of the liver, heart, reproductive fat pad, kidneys, spleen, brown fat, and skeletal muscle (gastrocnemius). We chose to weigh only the reproductive fat pad instead of all of the fat pads, since it is the largest and is strongly phenotypically (r = 0.67–0.82) and genetically correlated (h^2^ = 0.7–0.9) with the other fat pads [93, 94]. We submerged tissue from the liver and heart in RNAlater and then stored the samples in a -80C freezer. The mouse bones were cleaned with dermestid beetles, and then the long bones (radius, ulna, femur, and tibia) were weighed and their lengths were double-measured with calipers (Table D in S1 File and Fig 8), based on landmarks used by Kenney-Hunt *et al*. [95]. These skeletal dimensions provide an alternate measure of body size in addition to body weight, which is strongly influenced by obesity. The repeatabilities of the osteological measurements were all above 0.92 (Table D in S1 File).

### Gene expression

We used the Qiagen RNeasy Plus Mini kit to extract RNA from the liver tissue of the 80 F_1_ mice, and submitted it for RNA-seq with poly-A selection at the GTAC facility at Washington University in St. Louis. Quality control indicated that 74 of the 80 samples were of high enough quality for sequencing. A total of 37 libraries were sequenced, each with two mice of the same sex, maternal diet, and offspring diet pooled together. One exception was a HF-HF daughter library, which had 3 mice pooled together due to insufficient quantities of RNA. A 1x50 single read sequencing run was done on an Illumina HiSeq machine.

After the sequencing run, the FastQ files were aligned to the Ensembl release 76 assembly using STAR version 2.0.4b [96]. The transcript and gene counts were imported to the R package edgeR [97], a TMM normalization was performed to account for differences in library size, and genes with counts of zero were filtered out. The voom function in the R package Limma was then used to calculate the weighted likelihoods based on the mean-variance relationship of each gene. Differential expression was then tested with generalized linear models. Genes with an unadjusted p-value of less than 0.05 and a log fold change (logFC) with an absolute value greater than 2 were considered differentially expressed.

Two of the male libraries were determined to be outliers based on inspection of the MDS plots, and they were not included in the rest of the analysis. The remaining 35 libraries were analyzed: 4 libraries of HF-HF daughters, 5 LF-HF daughters, 5 HF-LF daughters, 4 LF-LF daughters, 5 HF-HF sons, 4 LF-HF sons, 4 HF-LF sons, and 4 libraries of LF-LF sons. The R package WGCNA [98] was used to build a tree from the libraries and then identify modules of genes with highly correlated expression levels. For each module that was significantly associated with at least one diabetes trait, we summarized the module based on its top ten significant Gene Ontology (GO) terms. We also used the R package GAGE [99] to identify pathways that were perturbed in a single direction or generally dysregulated due to maternal diet, and visualized those pathways using the R package Pathview [100].

We validated the differential expression of three genes with RT-qPCR in the HF daughters (*Mpo*, *Anxa2*, and *Chrna4*), using *Gapdh* as a normalizer. We extracted total RNA from the liver tissue of 3 HF-HF and 3 LF-HF daughters with Tri-Reagent (MRC), following the manufacturer’s instructions. The concentration and quality of the RNA from each sample was assessed twice with a NanoDrop Spectrophotometer, and we found that all samples had a 260/280 ratio between 1.7–2.1 and a 260/230 ratio between 2.0–2.4. We used the High-Capacity cDNA Reverse Transcription Kit (Applied Biosystems) to reverse transcribe the RNA to cDNA. We chose primers for qPCR from the literature and PrimerBank (https://pga.mgh.harvard.edu/primerbank/), and those primers were then synthesized by Thermo Fisher Scientific (Table M in S1 File). We used a reaction volume of 20 μL for the RT-qPCR, with 10 μL of PowerUp^™^ SYBR^®^ Green Master Mix (Thermo Fisher), 1 μL of the forward primer, 1 μL of the reverse primer, 4 μL of 20-fold diluted cDNA, and 4 μL of water. For each of the 3 biological replicates, we used 3 technical replicates, a no-template control, and a no-reverse-transcriptase control. A StepOnePlus Real-Time PCR System (Applied Biosystems) was used to perform the RT-qPCR under these conditions: 20 seconds at 95°C and then 40 cycles of 3 seconds at 95°C and 30 seconds at 60°C. We then used the comparative ΔΔCt method to perform a relative quantification of each of the three genes compared to *Gapdh* (Table N in S1 File).

### DNA methylation

We extracted DNA from the liver and heart tissue using a phenol-chloroform extraction. DNA methylation across the genome was then measured with Methylated DNA Immunoprecipitation Sequencing (MeDIP-seq) and Methylation-sensitive Restriction Enzyme Sequencing (MRE-seq) as previously described in detail by Li *et al*. [101]. MeDIP-seq reveals methylated sites, whereas MRE-seq reveals unmethylated sites; when used together they provide a genome-wide methylation map at single CpG resolution. This technique has high concordance with whole genome bisulfite sequencing at a fraction of the cost [102]. For the liver tissue, 4 mice of the same sex and from the same maternal diet and offspring diet treatment group were pooled per library, for 2 biological replicates per group and a total of 16 liver libraries. For the heart tissue, only the HF-HF and LF-HF daughters were analyzed, for a total of 4 heart libraries.

We used the R package MethlyMnM [103] to combine the MRE-seq data with the MeDIP-seq data and locate differentially methylated regions. MethlyMnM works by calculating the proportion of methylated CpGs in each window, and then determining the probability that the methylation level is statistically different between the two diet treatment groups. This is accomplished by performing a hypothesis test for each window based on the novel M&M test statistic. For this analysis, we removed blacklist sites and set the window size to 500 base pairs, which split the genome into 5,283,825 windows. Since there were two biological replicates per group and M&M can only do pairwise comparisons, we used Fisher’s combined probability test to compare offspring on the same diet who had mothers on different diets [104]. For example, the p-value from the M&M test comparing the HF-HF-daughters-1 vs. LF-HF-daughters-1 libraries was combined with the p-value from the M&M test comparing the HF-HF-daughters-2 vs. LF-HF-daughters-2 libraries.

$$X_{2k}^{2}\sim -2\overset{k}{\underset{i=1}{\sum }}\text{ln}\left(p_{i}\right)$$ (1)

*P*_*i*_ is the p-value from the M&M test, and since we combined p-values from *k* = 2 tests, *X* has a chi-squared distribution with 4 degrees of freedom. We then corrected for multiple comparisons using the Benjamini-Hochberg false discovery rate (FDR), and the resulting corrected combined q-values were used to determine which windows were differentially methylated. We determined how many windows had a q-value below the following significance thresholds: 0.05, 0.01, and 0.001.

We compared the replicates before combining the p-values with Fisher’s combined probability test. There were 35–39 significant DMRs when comparing the two HF female libraries (q < 0.05) and 7–2,816 significant DMRs when comparing the LF female libraries, whereas each comparison of libraries of females with different maternal diets had between 12 and 56 DMRs. The substantial methylation difference between two of the LF female libraries followed the trend we identified of LF mice having more methylation variation than HF mice. There were 4–223 significant DMRs when comparing the two HF male libraries (q<0.05) and 25–800 significant DMRs when comparing the LF male libraries, whereas each comparison of libraries of males with different maternal diets had between 6 and 983 DMRs. Few DMRs were found across multiple comparisons, whether between or within replicates. This highlights how variable methylation is, even in mice that are genetically identical and fed the same diet in the same facility. To ascertain which of these differences are meaningful, it is important to study multiple biological replicates, like we did here.

### Statistical analysis

For each obesity trait, differences between the diet treatments were analyzed by running a general linear model in SYSTAT (Version 12, Systat Software, San Jose, CA). A full model was run to test for the effect of sex, maternal diet, offspring diet, nurse ID, and the two-, three-, and four-way interactions of those variables. A reduced model tested only the effects of the maternal diet, offspring diet, sex, offspring-diet-by-sex, and offspring-diet-by-maternal diet terms. Multivariate tests were performed on three groups of traits: weekly weights, diabetes-related traits (week 15 and 16 weight, baseline glucose during IPGTT, IPGTT AUC, baseline glucose during IPITT, IPITT AUC, and food consumption), and necropsy traits (week 17 weight, serum biomarkers, and organ weights), in addition to all of the associated univariate tests. Differences were interpreted as significant for p-values less than 0.05.

For the gene expression analysis, any gene with an unadjusted p-value of less than 0.05 and a log fold change (logFC) with an absolute value greater than 2 was considered differentially expressed. For the methylation analysis, we combined the MRE-seq and MeDIP-seq data using the R package methylMnM [103] to find differentially methylated regions (DMRs) due to maternal diet. Since setting a significance threshold is somewhat arbitrary, we determined how many windows had a q-value below 0.05, 0.01, and 0.001. For each DMR, we identified the gene it was closest to, any genes or Ensembl regulatory elements it fell within (mouse genome assembly GRCm38.p5), if it fell within a promoter, and if the gene closest to it was previously known to be involved in diabetes mellitus, obesity, or cardiovascular diseases according to Phenopedia’s continuously updated list of genetic association studies (retrieved May 7, 2017) (Tables A-E in S2 File). We classified each DMR as being located in an intergenic region, intron, exon, or promoter by using the list of introns, exons, and genes in the NCBI37/mm9 assembly of the UCSC Genome Browser. If a DMR overlapped an exon as well as an intron, it was classified as falling within an exon. A DMR was labeled as being in a promoter if it was between 2,000 base pairs upstream of a transcription start site and 600 base pairs downstream of one. To establish if the DMRs were significantly associated with gene expression, we randomized the location of the DMRs across the genome and determined how many fell within differentially expressed genes by chance. To account for the observed underrepresentation of DMRs in intergenic regions, the percent of DMRs that were randomized into intergenic regions equaled the percent that actually exist in intergenic regions.

## Supporting information

S1 File Tables and Figures. Table A. P-values for effect of offspring diet. Table B. Averages of weekly weights, glucose and insulin tolerance values, and organ weights in sons. Table C. Averages of weekly weights, glucose and insulin tolerance values, and organ weights in daughters. Table D. Description of bone length measurements, repeatabilities, and p-values. Table E. Bone length averages. Table F. Differentially expressed genes due to maternal diet for high-fat-fed daughters (liver). Table G. Differentially expressed genes due to maternal diet for high-fat-fed daughters (heart). Table H. Differentially expressed genes due to maternal diet for low-fat-fed daughters. Table I. Differentially expressed genes due to maternal diet for high-fat-fed sons. Table J. Differentially expressed genes due to maternal diet for low-fat-fed sons. Table K. Significantly downregulated signaling and metabolism pathways due to maternal diet. Fig A. Non-alcoholic fatty liver disease (NAFLD) pathway diagrams. Fig B. Alzheimer’s disease pathway diagrams. Table L. Modules from WGCNA. Fig C. Plot from WGCNA analysis relating modules to the diabetes-related traits. Fig D. The expression levels of the *Mpo* and *Stat1* genes. Table M. Primers used for RT-qPCR. Table N. RT-qPCR validation results.
(DOCX)

S2 File Differentially methylated regions—Tables. Table A. HF-HFvLF-HF Female Liver DMRs. Table B. HF-HFvLF-HF Female Heart DMRs. Table C. HF-LFvLF-LF Female DMRs. Table D. HF-HF v LF-HF Male DMRs. Table E. HF-LF v LF-LF Male DMRs.
(XLSX)

## References

[1] Wolff GL, Kodell RL, Moore SR, Cooney CA. Maternal epigenetics and methyl supplements affect agouti gene expression in Avy/a mice. FASEB J. 1998;12: 949–957. 9707167

[2] Waterland RA, Jirtle RL. Transposable elements: targets for early nutritional effects on epigenetic gene regulation. Mol Cell Biol. 2003;23: 5293–5300. doi: 10.1128/MCB.23.15.5293-5300.2003 12861015 10.1128/MCB.23.15.5293-5300.2003 PMC165709

[3] Dolinoy DC, Weidman JR, Waterland RA, Jirtle RL. Maternal genistein alters coat color and protects Avy mouse offspring from obesity by modifying the fetal epigenome. Environmental Health Perspectives. 2006;114: 567–572. doi: 10.1289/ehp.8700 16581547 10.1289/ehp.8700 PMC1440782

[4] Oestreich AK, Moley KH. Developmental and transmittable origins of obesity-associated health disorders. Trends Genet. 2017;33: 399–407. doi: 10.1016/j.tig.2017.03.008 28438343 10.1016/j.tig.2017.03.008 PMC5875684

[5] Branum AM, Kirmeyer SE, Gregory EC. Prepregnancy body mass index by maternal characteristics and state: data from the birth certificate, 2014. Natl. Vital Stat. Rep. 2016;65: 1–11. 27508894

[6] Yao R, Ananth CV, Park BY, Pereira L, Plante LA. Perinatal Research Consortium, Obesity and the risk of stillbirth: a population-based cohort study. Am J Obstet Gynecol. 2014;210: 457.e1–457.e9. 24674712 10.1016/j.ajog.2014.01.044

[7] Chu SY, Kim SY, Lau J, Schmid CH, Dietz PM, Callaghan WM, et al Maternal obesity and risk of stillbirth: a metaanalysis. Am. J. Obstet. Gynecol. 2007;197: 223–228. doi: 10.1016/j.ajog.2007.03.027 17826400 10.1016/j.ajog.2007.03.027

[8] Poston L, Harthoorn LF, van der Beek EM. Obesity in pregnancy: implications for the mother and lifelong health of the child: a consensus statement. Pediatric Research. 2011;69: 175–180. doi: 10.1203/PDR.0b013e3182055ede 21076366 10.1203/PDR.0b013e3182055ede

[9] Rasmussen SA, Chu SY, Kim SY, Schmid CH, Lau J. Maternal obesity and risk of neural tube defects: a metaanalysis. Am J Obstet Gynecol. 2008;198: 611–619. doi: 10.1016/j.ajog.2008.04.021 18538144 10.1016/j.ajog.2008.04.021

[10] Catalano PM, Presley L, Minium J, Hauguel-de Mouzon S. Fetuses of obese mothers develop insulin resistance in utero. Diabetes Care. 2009;32: 1076–1080. doi: 10.2337/dc08-2077 19460915 10.2337/dc08-2077 PMC2681036

[11] Whitaker RC. Predicting preschooler obesity at birth: the role of maternal obesity in early pregnancy. Pediatrics. 2004;114: e29–e36. 15231970 10.1542/peds.114.1.e29

[12] Hillier TA, Pedula KL, Schmidt MM, Mullen JA, Charles M-A, Pettitt DJ. Childhood obesity and metabolic imprinting: the ongoing effects of maternal hyperglycemia. Diabetes Care. 2007;30: 2287–2292. doi: 10.2337/dc06-2361 17519427 10.2337/dc06-2361

[13] Yu Z, Han S, Zhu J, Sun X, Ji C, Guo X. Pre-pregnancy body mass index in relation to infant birth weight and offspring overweight/obesity: a systematic review and meta-analysis. PLoS One. 2013;8: e61627 doi: 10.1371/journal.pone.0061627 23613888 10.1371/journal.pone.0061627 PMC3628788

[14] Gaillard R, Steegers EA, Duijts L, Felix JF, Hofman A, Franco OH, et al Childhood cardiometabolic outcomes of maternal obesity during pregnancy: the Generation R Study. Hypertension. 2014;63: 683–691. doi: 10.1161/HYPERTENSIONAHA.113.02671 24379180 10.1161/HYPERTENSIONAHA.113.02671

[15] Mina TH, Lahti M, Drake AJ, Räikkönen K, Minnis H, Denison FC, et al Prenatal exposure to very severe maternal obesity is associated with adverse neuropsychiatric outcomes in children. Psychol Med. 2017;47: 353–362. doi: 10.1017/S0033291716002452 27776561 10.1017/S0033291716002452

[16] Pugh SJ, Richardson GA, Hutcheon JA, Himes KP, Brooks MM, Day NL, et al Maternal obesity and excessive gestational weight gain are associated with components of child cognition. J Nutr. 2015;145: 2562–2569. doi: 10.3945/jn.115.215525 26423736 10.3945/jn.115.215525 PMC4620725

[17] Hochner H, Friedlander Y, Calderon-Margalit R, Meiner V, Sagy Y, Avgil-Tsadok M, et al Associations of maternal prepregnancy body mass index and gestational weight gain with adult offspring cardiometabolic risk factors: the Jerusalem Perinatal Family Follow-up Study. Circulation. 2012;125: 1381–1389. doi: 10.1161/CIRCULATIONAHA.111.070060 22344037 10.1161/CIRCULATIONAHA.111.070060 PMC3332052

[18] Eriksson JG, Sandboge S, Salonen MK, Kajantie E, Osmond C. Long-term consequences of maternal overweight in pregnancy on offspring later health: findings from the Helsinki Birth Cohort Study. Ann. Med. 2014;46: 434–438. doi: 10.3109/07853890.2014.919728 24910160 10.3109/07853890.2014.919728

[19] Stirrat LI, Reynolds RM. Effects of maternal obesity on early and long-term outcomes for offspring. Research and Reports in Neonatology. 2014;4: 43–53.

[20] Gaillard R. Maternal obesity during pregnancy and cardiovascular development and disease in the offspring. Eur J Epidemiol. 2015;30: 1141–1152. doi: 10.1007/s10654-015-0085-7 26377700 10.1007/s10654-015-0085-7 PMC4684830

[21] Liang C, Oest ME, Prater MR. Intrauterine exposure to high saturated fat diet elevates risk of adult-onset chronic diseases in C57BL/6 mice. Birth Defects Res B Dev Reprod Toxicol. 2009;86: 377–384. doi: 10.1002/bdrb.20206 19750488 10.1002/bdrb.20206

[22] Elahi MM, Cagampang FR, Mukhtara D, Anthony FW, Ohri SK, Hanson MA. Long-term maternal high-fat feeding from weaning through pregnancy and lactation predisposes offspring to hypertension, raised plasma lipids and fatty liver in mice. British Journal of Nutrition. 2009;102: 514–519. doi: 10.1017/S000711450820749X 19203419 10.1017/S000711450820749X

[23] Wankhade UD, Zhong Y, Kang P, Alfaro M, Chintapalli SV, Thakali KM, et al Enhanced offspring predisposition to steatohepatitis with maternal high-fat diet is associated with epigenetic and microbiome alterations. PLoS One. 2017;12: e0175675 doi: 10.1371/journal.pone.0175675 28414763 10.1371/journal.pone.0175675 PMC5393586

[24] Fernandez-Twinn DS, Blackmore HL, Siggens L, Giussani DA, Cross CM, Foo R, Ozanne SE. The programming of cardiac hypertrophy in the offspring by maternal obesity is associated with hyperinsulinemia, AKT, ERK, and mTOR activation. Endocrinology. 2012;153: 5961–5971. doi: 10.1210/en.2012-1508 23070543 10.1210/en.2012-1508 PMC3568261

[25] Blackmore HL, Niu Y, Fernandez-Twinn DS, Tarry-Adkins JL, Giussani DA, Ozanne SE. Maternal diet-induced obesity programs cardiovascular dysfunction in adult male mouse offspring independent of current body weight. Endocrinology. 2014;155: 3970–3980. doi: 10.1210/en.2014-1383 25051449 10.1210/en.2014-1383 PMC4255219

[26] Khan I, Dekou V, Hanson M, Poston L, Taylor P. Predictive adaptive responses to maternal high-fat diet prevent endothelial dysfunction but not hypertension in adult rat offspring. Circulation. 2004;110: 1097–1102. doi: 10.1161/01.CIR.0000139843.05436.A0 15326063 10.1161/01.CIR.0000139843.05436.A0

[27] Bellisario V, Berry A, Capoccia S, Raggi C, Panetta P, Branchi I, et al Gender-dependent resiliency to stressful and metabolic challenges following prenatal exposure to high-fat diet in the p66Shc−/− mouse. Front. Behav. Neurosci. 2014;8: 285 doi: 10.3389/fnbeh.2014.00285 25202246 10.3389/fnbeh.2014.00285 PMC4141279

[28] Miller C, Krishna S, Zhoumeng L, Della-Fera MA, Harn D, de la Serre C, et al Early sex differences in hepatic metabolic signaling in offspring of obese female mice. FASEB. 2014;28: Supplement 1033.11.

[29] Mischke M, Pruis MGM, Boekschoten MV, Groen AK, Fitri AR, van de Heijning BJM, et al Maternal western-style high fat diet induces sex-specific physiological and molecular changes in two-week-old mouse offspring. PLoS ONE. 2013;8: e78623 doi: 10.1371/journal.pone.0078623 24223833 10.1371/journal.pone.0078623 PMC3818485

[30] Jungheim ES, Schoeller EL, Marquard KL, Louden ED, Schaffer JE, Moley KH. Diet-induced obesity model: abnormal oocytes and persistent growth abnormalities in the offspring. Endocrinology. 2010;151: 4039–4046. doi: 10.1210/en.2010-0098 20573727 10.1210/en.2010-0098 PMC2940512

[31] Leary C, Leese HJ, Sturmey RG. Human embryos from overweight and obese women display phenotypic and metabolic abnormalities. Hum. Reprod. 2015;30: 122–132. doi: 10.1093/humrep/deu276 25391239 10.1093/humrep/deu276

[32] Wang D, Chen S, Liu M, Liu C. Maternal obesity disrupts circadian rhythms of clock and metabolic genes in the offspring heart and liver. Chronobiol Int. 2015;32: 615–626. doi: 10.3109/07420528.2015.1025958 25928088 10.3109/07420528.2015.1025958

[33] Igosheva N, Abramov AY, Poston L, Eckert JJ, Fleming TP, Duchen MR, et al Maternal diet-induced obesity alters mitochondrial activity and redox status in mouse oocytes and zygotes. PLoS One. 2010;5: e10074 doi: 10.1371/journal.pone.0010074 20404917 10.1371/journal.pone.0010074 PMC2852405

[34] Luzzo KM, Wang Q, Purcell SH, Chi M, Jimenez PT, Grindler N, et al High fat diet induced developmental defects in the mouse: oocyte meiotic aneuploidy and fetal growth retardation/brain defects. PLoS One. 2012;7: e49217 doi: 10.1371/journal.pone.0049217 23152876 10.1371/journal.pone.0049217 PMC3495769

[35] Grindler NM, Moley KH. Maternal obesity, infertility and mitochondrial dysfunction: potential mechanisms emerging from mouse model systems. Mol Hum Reprod. 2013;19: 486–494. doi: 10.1093/molehr/gat026 23612738 10.1093/molehr/gat026 PMC3712655

[36] Bar J, Schreiber L, Saruhanov E, Ben-Haroush A, Golan A, Kovo M. Placental histopathological findings in obese and nonobese women with complicated and uncomplicated pregnancies. Arch. Gynecol. Obstet. 2012;286: 1343–1347. doi: 10.1007/s00404-012-2450-z 22797660 10.1007/s00404-012-2450-z

[37] Sferruzzi-Perri AN, Vaughan OR, Haro M, Cooper WG, Musial B, Charalambous M, et al An obesogenic diet during mouse pregnancy modifies maternal nutrient partitioning and the fetal growth trajectory. FASEB J. 2013;27: 3928–3937. doi: 10.1096/fj.13-234823 23825226 10.1096/fj.13-234823

[38] Roberts KA, Riley SC, Reynolds RM, Barr S, Evans M, Statham A, et al Placental structure and inflammation in pregnancies associated with obesity. Placenta. 2011;32: 247–254. doi: 10.1016/j.placenta.2010.12.023 21232790 10.1016/j.placenta.2010.12.023

[39] Soubry A, Murphy SK, Wang F, Huang Z, Vidal AC, Fuemmeler BF, et al Newborns of obese parents have altered DNA methylation patterns at imprinted genes. International Journal of Obesity. 2015;39: 650–657. doi: 10.1038/ijo.2013.193 24158121 10.1038/ijo.2013.193 PMC4048324

[40] Guénard F, Deshaies Y, Cianflone K, Kral JG, Marceau P, Vohl M-C. Differential methylation in glucoregulatory genes of offspring born before vs. after maternal gastrointestinal bypass surgery. PNAS. 2013;110: 11439–11444. doi: 10.1073/pnas.1216959110 23716672 10.1073/pnas.1216959110 PMC3710842

[41] Cao JJ. Effects of obesity on bone metabolism. J. Orthop. Surg. Res. 2011;6: 30 doi: 10.1186/1749-799X-6-30 21676245 10.1186/1749-799X-6-30 PMC3141563

[42] Brabant G, Müller G, Horn R, Anderwald C, Roden M, Nave H. Hepatic leptin signaling in obesity. FASEB J. 2005; 19:1048–1050. doi: 10.1096/fj.04-2846fje 15788447 10.1096/fj.04-2846fje

[43] Huynh FK, Neumann UH, Wang Y, Rodrigues B, Kieffer TJ, Covey SD. A role for hepatic leptin signaling in lipid metabolism via altered very low density lipoprotein composition and liver lipase activity in mice. Hepatology. 2013;57: 543–554. doi: 10.1002/hep.26043 22941940 10.1002/hep.26043

[44] Zain SM, Mohamed Z, Mahadeva S, Cheah PL, Rampal S, Chin KF, et al Impact of leptin receptor gene variants on risk of non-alcoholic fatty liver disease and its interaction with adiponutrin gene. J Gastroenterol Hepatol. 2013;28: 873–879. doi: 10.1111/jgh.12104 23278404 10.1111/jgh.12104

[45] Ashino NG, Saito KN, Souza FD, Nakutz FS, Roman EA, Velloso LA, et al Maternal high-fat feeding through pregnancy and lactation predisposes mouse offspring to molecular insulin resistance and fatty liver. J. Nutr. Biochem. 2012;23: 341–348. doi: 10.1016/j.jnutbio.2010.12.011 21543214 10.1016/j.jnutbio.2010.12.011

[46] Pessayre D, Fromenty B. NASH: a mitochondrial disease. J Hepatol. 2005;42: 928–940. doi: 10.1016/j.jhep.2005.03.004 15885365 10.1016/j.jhep.2005.03.004

[47] Wei Y, Rector RS, Thyfault JP, Ibdah JA. Nonalcoholic fatty liver disease and mitochondrial dysfunction. World J Gastroenterol. 2008;14: 193–199. doi: 10.3748/wjg.14.193 18186554 10.3748/wjg.14.193 PMC2675113

[48] Mouralidarane A, Soeda J, Visconti-Pugmire C, Samuelsson AM, Pombo J, Maragkoudaki X, et al Maternal obesity programs offspring nonalcoholic fatty liver disease by innate immune dysfunction in mice. Hepatology. 2013;58: 128–138. doi: 10.1002/hep.26248 23315950 10.1002/hep.26248

[49] Mouralidarane A, Soeda J, Sugden D, Bocianowska A, Carter R, Ray S, et al Maternal obesity programs offspring non-alcoholic fatty liver disease through disruption of 24-h rhythms in mice. Int J Obes (Lond). 2015;39: 1339–1348. 25971926 10.1038/ijo.2015.85

[50] Kim D-G, Krenz A, Toussaint LE, Maurer KJ, Robinson S-A, Yan A, et al Non-alcoholic fatty liver disease induces signs of Alzheimer’s disease (AD) in wild-type mice and accelerates pathological signs of AD in an AD model. J Neuroinflammation. 2016;13: 1 doi: 10.1186/s12974-015-0467-5 26728181 10.1186/s12974-015-0467-5 PMC4700622

[51] Satoh K, Matsu-Ura T, Enomoto M, Nakamura H, Michikawa T, Mikoshiba K. Highly cooperative dependence of sarco/endoplasmic reticulum calcium ATPase SERCA2a pump activity on cytosolic calcium in living cells. J Biol Chem. 2011;286: 20591–20599. doi: 10.1074/jbc.M110.204685 21515674 10.1074/jbc.M110.204685 PMC3121519

[52] Sutcliffe JG, Hedlund PB, Thomas EA, Bloom FE, Hilbush BS. Peripheral reduction of β-amyloid is sufficient to reduce brain β-amyloid: implications for Alzheimer's disease. J Neurosci Res. 2011;89: 808–814. doi: 10.1002/jnr.22603 21374699 10.1002/jnr.22603

[53] Wildsmith KR, Holley M, Savage JC, Skerrett R, Landreth GE. Evidence for impaired amyloid β clearance in Alzheimer's disease. Alzheimers Res Ther. 2013;5: 33 doi: 10.1186/alzrt187 23849219 10.1186/alzrt187 PMC3978761

[54] Saharan S, Mandal PK. The emerging role of glutathione in Alzheimer's disease. J Alzheimers Dis. 2014;40: 519–529. doi: 10.3233/JAD-132483 24496077 10.3233/JAD-132483

[55] Pocernich CB, Butterfield DA. Elevation of glutathione as a therapeutic strategy in Alzheimer disease. Biochim Biophys Acta. 2012;1822: 625–630. doi: 10.1016/j.bbadis.2011.10.003 22015471 10.1016/j.bbadis.2011.10.003 PMC3277671

[56] Kivipelto M, Ngandu T, Fratiglioni L, Viitanen M, Kåreholt I, Winblad B, et al Obesity and vascular risk factors at midlife and the risk of dementia and Alzheimer disease. Arch Neurol. 2005;62: 1556–1560. doi: 10.1001/archneur.62.10.1556 16216938 10.1001/archneur.62.10.1556

[57] Pugazhenthi S, Qin L, Reddy PH. Common neurodegenerative pathways in obesity, diabetes, and Alzheimer's disease. Biochim Biophys Acta. 2017;1863: 1037–1045. doi: 10.1016/j.bbadis.2016.04.017 27156888 10.1016/j.bbadis.2016.04.017 PMC5344771

[58] Costa SM, Isganaitis E, Matthews TJ, Hughes K, Daher G, Dreyfuss JM, et al Maternal obesity programs mitochondrial and lipid metabolism gene expression in infant umbilical vein endothelial cells. Int. J. Obes. 2016;40: 1627–1634. 10.1038/ijo.2016.142 PMC5101152 27531045

[59] Zhu MJ, Han B, Tong J, Ma C, Kimzey JM, Underwood KR, et al AMP-activated protein kinase signalling pathways are down regulated and skeletal muscle development impaired in fetuses of obese, over-nourished sheep. J Physiol. 2016;586: 2651–2664. 10.1113/jphysiol.2007.149633 PMC2464338 18372306

[60] Latouche C, Heywood SE, Henry SL, Ziemann M, Lazarus R, El-Osta A, et al Maternal overnutrition programs changes in the expression of skeletal muscle genes that are associated with insulin resistance and defects of oxidative phosphorylation in adult male rat offspring. J Nutr. 2014;144: 237–244. doi: 10.3945/jn.113.186775 24381224 10.3945/jn.113.186775

[61] Zordoky BN, El-Kadi AO. Modulation of cardiac and hepatic cytochrome P450 enzymes during heart failure. Curr Drug Metab. 2008;9: 122–128. 18288954 10.2174/138920008783571792

[62] Joshi SR, Lakhkar A, Dhagia V, Zias AL, Soldatos V, Oshima K, et al Cyp2c44 gene disruption exacerbated pulmonary hypertension and heart failure in female but not male mice Pulm Circ. 2016;6: 360–368. doi: 10.1086/688060 27683613 10.1086/688060 PMC5019089

[63] El-Maarri O, Becker T, Junen J, Manzoor SS, Diaz-Lacava A, Schwaab R, et al Gender specific differences in levels of DNA methylation at selected loci from human total blood: a tendency toward higher methylation levels in males. Hum. Genet. 2007;122: 505–514. doi: 10.1007/s00439-007-0430-3 17851693 10.1007/s00439-007-0430-3

[64] Numata S, Ye T, Hyde TM, Guitart-Navarro X, Tao R, Wininger M, et al DNA methylation signatures in development and aging of the human prefrontal cortex. Am J Hum Genet. 2012;90: 260–272. doi: 10.1016/j.ajhg.2011.12.020 22305529 10.1016/j.ajhg.2011.12.020 PMC3276664

[65] Goto T, Monk M. Regulation of X-chromosome inactivation in development in mice and humans. Microbiol Mol Biol Rev. 1998;62: 362–378. 9618446 10.1128/mmbr.62.2.362-378.1998 PMC98919

[66] Sharp AJ, Stathaki E, Migliavacca E, Brahmachary M, Montgomery SB, Dupre Y, et al DNA methylation profiles of human active and inactive X chromosomes. Genome Res. 2011;21: 1592–1600. doi: 10.1101/gr.112680.110 21862626 10.1101/gr.112680.110 PMC3202277

[67] Muka T, Nano J, Voortman T, Braun KVE, Ligthart S, Stranges S, et al The role of global and regional DNA methylation and histone modifications in glycemic traits and type 2 diabetes: A systematic review. Nutr Metab Cardiovasc Dis. 2016;26: 553–566. doi: 10.1016/j.numecd.2016.04.002 27146363 10.1016/j.numecd.2016.04.002

[68] Rönn T, Volkov P, Davegårdh C, Dayeh T, Hall E, Olsson AH, et al A six months exercise intervention influences the genome-wide DNA methylation pattern in human adipose tissue. PLoS Genet. 2013;9: e1003572 doi: 10.1371/journal.pgen.1003572 23825961 10.1371/journal.pgen.1003572 PMC3694844

[69] Nilsson E, Jansson PA, Perfilyev A, Volkov P, Pedersen M, Svensson MK, et al Altered DNA methylation and differential expression of genes influencing metabolism and inflammation in adipose tissue from subjects with type 2 diabetes. Diabetes. 2014;63: 2962–2976. doi: 10.2337/db13-1459 24812430 10.2337/db13-1459

[70] Yang BT, Dayeh TA, Kirkpatrick CL, Taneera J, Kumar R, Groop L, et al Insulin promoter DNA methylation correlates negatively with insulin gene expression and positively with HbA1c levels in human pancreatic islets. Diabetologia. 2011;54: 360–367. doi: 10.1007/s00125-010-1967-6 21104225 10.1007/s00125-010-1967-6 PMC3017313

[71] Castan-Laurell I, Dray C, Attané C, Duparc T, Knauf C, Valet P. Apelin, diabetes, and obesity. Endocrine. 2011;40: 1–9. doi: 10.1007/s12020-011-9507-9 21725702 10.1007/s12020-011-9507-9

[72] Inuzuka H, Nishizawa H, Inagaki A, Suzuki M, Ota S, Miyamura H, et al Decreased expression of apelin in placentas from severe pre-eclampsia patients. Hypertens Pregnancy. 2013;32: 410–421. doi: 10.3109/10641955.2013.813535 23844873 10.3109/10641955.2013.813535

[73] Liang M, Niu J, Zhang L, Deng H, Ma J, Zhou W, et al Gene expression profiling reveals different molecular patterns in G-protein coupled receptor signaling pathways between early- and late-onset preeclampsia. Placenta. 2016;40: 52–59. doi: 10.1016/j.placenta.2016.02.015 27016783 10.1016/j.placenta.2016.02.015

[74] Liao YC, Chou WW, Li YN, Chuang SC, Lin WY, Lakkakula BV, et al Apelin gene polymorphism influences apelin expression and obesity phenotypes in Chinese women. Am J Clin Nutr. 2011;94: 921–928. doi: 10.3945/ajcn.110.008813 21775567 10.3945/ajcn.110.008813

[75] Aboouf MA, Hamdy NM, Amin AI, El-Mesallamy HO. Genotype screening of APLN rs3115757 variant in Egyptian women population reveals an association with obesity and insulin resistance. Diabetes Res. Clin. Pract. 2015;109: 40–47. doi: 10.1016/j.diabres.2015.05.016 26025696 10.1016/j.diabres.2015.05.016

[76] Yokomori H, Oda M, Yoshimura K, Hibi T. Enhanced expressions of apelin on proliferative hepatic arterial capillaries in human cirrhotic liver. Hepatol Res. 2012;42: 508–514. doi: 10.1111/j.1872-034X.2011.00945.x 22502744 10.1111/j.1872-034X.2011.00945.x

[77] Wang Q, Xie Z, Zhang W, Zhou J, Wu Y, Zhang M, et al Myeloperoxidase deletion prevents high-fat diet-induced obesity and insulin resistance. Diabetes. 2014;63: 4172–4185. doi: 10.2337/db14-0026 25024373 10.2337/db14-0026 PMC4238009

[78] Stenvinkel P, Rodríguez-Ayala E, Massy ZA, Qureshi AR, Barany P, Fellström B, et al Statin treatment and diabetes affect myeloperoxidase activity in maintenance hemodialysis patients. Clin J Am Soc Nephrol. 2006;1: 281–287. doi: 10.2215/CJN.01281005 17699218 10.2215/CJN.01281005

[79] Rensen SS, Slaats Y, Nijhuis J, Jans A, Bieghs V, Driessen A, et al Increased hepatic myeloperoxidase activity in obese subjects with nonalcoholic steatohepatitis. Am J Pathol. 2009;175: 1473–1482. doi: 10.2353/ajpath.2009.080999 19729473 10.2353/ajpath.2009.080999 PMC2751544

[80] Keaney JF Jr, Larson MG, Vasan RS, Wilson PW, Lipinska I, Corey D, et al; Framingham Study. Obesity and systemic oxidative stress: clinical correlates of oxidative stress in the Framingham Study. Arterioscler Thromb Vasc Biol. 2003;23: 434–439. doi: 10.1161/01.ATV.0000058402.34138.11 12615693 10.1161/01.ATV.0000058402.34138.11

[81] Fernández-Sánchez A, Madrigal-Santillán E, Bautista M, Esquivel-Soto J, Morales-González Á, Esquivel-Chirino C, et al Inflammation, oxidative stress, and obesity. Int J Mol Sci. 2011;12: 3117–3132. doi: 10.3390/ijms12053117 21686173 10.3390/ijms12053117 PMC3116179

[82] Hauguel-de Mouzon S, Guerre-Millo M. The placenta cytokine network and inflammatory signals. Placenta. 2006;27: 794–798. doi: 10.1016/j.placenta.2005.08.009 16242770 10.1016/j.placenta.2005.08.009

[83] Madan JC, Davis JM, Craig WY, Collins M, Allan W, Quinn R, et al Maternal obesity and markers of inflammation in pregnancy. Cytokine. 2009;47: 61–66. doi: 10.1016/j.cyto.2009.05.004 19505831 10.1016/j.cyto.2009.05.004

[84] Bilbo SD, Tsang V. Enduring consequences of maternal obesity for brain inflammation and behavior of offspring. FASEB J. 2014;24: 2104–2115. 10.1096/fj.09-144014 20124437

[85] Gao B. Cytokines, STATs and liver disease. Cell Mol Immunol. 2005;2: 92–100. 16191414

[86] Deng F-Y, Lei S-F, Zhang Y, Zhang Y-L, Zheng Y-P, Zhang L-S, et al Peripheral blood monocyte-expressed ANXA2 gene is involved in pathogenesis of osteoporosis in humans. Molecular & Cellular Proteomics. 2011;10: M111.011700. 10.1074/mcp.M111.011700 PMC3226411 21817168

[87] Levy E, Lalonde G, Delvin E, Elchebly M, Précourt LP, Seidah NG, et al Intestinal and hepatic cholesterol carriers in diabetic Psammomys obesus. Endocrinology. 2010;151: 958–970. doi: 10.1210/en.2009-0866 20130116 10.1210/en.2009-0866

[88] Do GM, Oh HY, Kwon EY, Cho YY, Shin SK, Park HJ, et al Long-term adaptation of global transcription and metabolism in the liver of high-fat diet-fed C57BL/6J mice. Mol. Nutr. Food Res. 2011;55: S173–S185. doi: 10.1002/mnfr.201100064 21618427 10.1002/mnfr.201100064

[89] MacArthur J. Genetics of body size and related characters. I. Selection of small and large races of the laboratory mouse. Amer Natur. 1944;78: 142–157.

[90] Cheverud JM, Pletscher LS, Vaughn TT, Marshall BM. Differential response to dietary fat in large (LG/J) and small (SM/J) inbred mouse strains. Physiol. Genomics. 1999;15: 33–39. 10.1152/physiolgenomics.1999.1.1.33 11015559

[91] Ehrich TH, Kenney JP, Vaughn TT, Pletscher LS, Cheverud JM. Diet, obesity, and hyperglycemia in LG/J and SM/J mice. Obesity Research. 2003;11: 1400–1410. doi: 10.1038/oby.2003.189 14627762 10.1038/oby.2003.189

[92] Partridge C, Fawcett GL, Wang B, Semenkovich CF, Cheverud JM. The effect of dietary fat intake on hepatic gene expression in LG/J and SM/J mice. BMC Genomics. 2014;15: 99 doi: 10.1186/1471-2164-15-99 24499025 10.1186/1471-2164-15-99 PMC4028868

[93] Cheverud JM, Ehrich TH, Kenney JP, Pletscher LS, Semenkovich CF. Genetic evidence for discordance between obesity- and diabetes-related traits in the LGXSM recombinant inbred mouse strains. Diabetes. 2004;53, 2700–2708. 15448104 10.2337/diabetes.53.10.2700

[94] Cheverud JM, Lawson HA, Fawcett GL, Wang B, Pletscher LS, Fox AR, et al Diet-dependent genetic and genomic imprinting effects on obesity in mice. Obesity. 2011;19: 160–170. doi: 10.1038/oby.2010.141 20539295 10.1038/oby.2010.141 PMC3677968

[95] Kenney-Hunt JP, Wang B, Norgard EA, Fawcett G, Falk D, Pletscher LS, et al Pleiotropic patterns of quantitative trait loci for 70 murine skeletal traits. Genetics. 2008;178: 2275–2288. doi: 10.1534/genetics.107.084434 18430949 10.1534/genetics.107.084434 PMC2323815

[96] Dobin A, Davis CA, Schlesinger F, Drenkow J, Zaleski C, Jha S, et al STAR: ultrafast universal RNA-seq aligner. Bioinformatics. 2013;29: 15–21. doi: 10.1093/bioinformatics/bts635 23104886 10.1093/bioinformatics/bts635 PMC3530905

[97] Robinson MD, McCarthy DJ, Smyth GK. edgeR: a Bioconductor package for differential expression analysis of digital gene expression data. Bioinformatics. 2010;26: 139–140. doi: 10.1093/bioinformatics/btp616 19910308 10.1093/bioinformatics/btp616 PMC2796818

[98] Langfelder P, Horvath S. WGCNA: an R package for weighted correlation network analysis. BMC Bioinformatics. 2008;9: 559 doi: 10.1186/1471-2105-9-559 19114008 10.1186/1471-2105-9-559 PMC2631488

[99] Luo W, Friedman M, Shedden K, Hankenson K, Woolf P. GAGE: generally applicable gene set enrichment for pathway analysis. BMC Bioinformatics. 2009;10: 161 doi: 10.1186/1471-2105-10-161 19473525 10.1186/1471-2105-10-161 PMC2696452

[100] Luo W, Brouwer C. Pathview: an R/Bioconductor package for pathway-based data integration and visualization. Bioinformatics. 2013;29: 1830–1831. doi: 10.1093/bioinformatics/btt285 23740750 10.1093/bioinformatics/btt285 PMC3702256

[101] Li D, Zhang B, Xing X, Wang T. Combining MeDIP-seq and MRE-seq to investigate genome-wide CpG methylation. Methods. 2015;72: 29–40. doi: 10.1016/j.ymeth.2014.10.032 25448294 10.1016/j.ymeth.2014.10.032 PMC4300244

[102] Stevens MJ, Cheng JB, Li D, Xie M, Hong C, Maire CL, et al Estimating absolute methylation levels at single CpG resolution from methylation enrichment and restriction enzyme sequencing methods. Genome Res. 2013;23: 1541–1553. doi: 10.1101/gr.152231.112 23804401 10.1101/gr.152231.112 PMC3759729

[103] Zhang B, Zhou Y, Lin N, Lowdon RF, Hong C, Nagarajan RP, et al Functional DNA methylation differences between tissues, cell types, and across individuals discovered using the M&M algorithm. Genome Res. 2013;23: 1522–1540. doi: 10.1101/gr.156539.113 23804400 10.1101/gr.156539.113 PMC3759728

[104] Fisher RA, Statistical Methods for Research Workers. 12^th^ ed Oliver & Boyd, Edinburgh, 356 pp (1954).

