# Supplementary material for: Maternal high-fat diet associated with altered gene expression, DNA methylation, and obesity risk in mouse offspring
Source: PLoS One. 2018 Feb 15;13(2):e0192606. doi: 10.1371/journal.pone.0192606 (PMC5813940; doi:10.1371/journal.pone.0192606)
Supplement: S1 File — Table A. P-values for effect of offspring diet. Table B. Averages of weekly weights, glucose and insulin tolerance values, and organ weights in sons. Table C. Averages of weekly weights, glucose and insulin tolerance values, and organ weights in daughters. Table D. Description of bone length measurements, repeatabilities, and p-values. Table E. Bone length averages. Table F. Differentially expressed genes due to maternal diet for high-fat-fed daughters (liver). Table G. Differentially expressed genes due to maternal diet for high-fat-fed daughters (heart). Table H. Differentially expressed genes due to maternal diet for low-fat-fed daughters. Table I. Differentially expressed genes due to maternal diet for high-fat-fed sons. Table J. Differentially expressed genes due to maternal diet for low-fat-fed sons. Table K. Significantly downregulated signaling and metabolism pathways due to maternal diet. Fig A. Non-alcoholic fatty liver disease (NAFLD) pathway diagrams. Fig B. Alzheimer’s disease pathway diagrams. Table L. Modules from WGCNA. Fig C. Plot from WGCNA analysis relating modules to the diabetes-related traits. Fig D. The expression levels of the Mpo and Stat1 genes. Table M. Primers used for RT-qPCR. Table N. RT-qPCR validation results. (DOCX) [file pone.0192606.s001.docx]

**S1 File: Tables and Figures**

**Table A. P-values for offspring diet.** Offspring diet significantly affected nearly all of the traits measured, N = 40 males and 40 females.

| Trait | Females p-value | Males p-value |
| --- | --- | --- |
| WK1 weight | 0.981 | 0.731 |
| WK2 weight | 0.722 | 0.989 |
| WK3 weight | 0.996 | 0.346 |
| WK4 weight | **4.00E-05** | **2.46E-06** |
| WK5 weight | **4.61E-09** | **2.22E-11** |
| WK6 weight | **9.45E-08** | **8.96E-10** |
| WK7 weight | **6.09E-09** | **2.02E-13** |
| WK8 weight | **5.35E-07** | **5.00E-15** |
| WK9 weight | **1.02E-09** | **4.70E-04** |
| WK10 weight | **1.01E-13** | **2.05E-18** |
| WK11 weight | **4.85E-12** | **4.94E-16** |
| WK12 weight | **2.47E-11** | **5.24E-18** |
| WK13 weight | **3.87E-11** | **1.47E-16** |
| WK14 weight | **8.98E-13** | **1.26E-14** |
| WK15 weight | **1.83E-13** | **5.10E-21** |
| WK16 weight | **2.83E-08** | **2.27E-21** |
| WK17 weight | **1.94E-14** | **7.60E-21** |
| Food consumption | **2.66E-15** | **1.67E-15** |
| Baseline glucose in IPGTT | **6.89E-08** | **4.95E-09** |
| IPGTT AUC | **1.59E-11** | **2.41E-12** |
| Baseline glucose in IPITT | **8.81E-07** | **1.48E-07** |
| IPITT AUC | **1.40E-09** | **2.13E-12** |
| Liver weight | **3.93E-06** | **1.38E-12** |
| Fat pad weight | **1.73E-12** | **5.32E-12** |
| Heart weight | **1.00E-05** | **1.00E-05** |
| Left kidney weight | **2.63E-06** | **1.00E-05** |
| Right kidney weight | **2.55E-06** | **2.84E-06** |
| Spleen weight | **2.86E-09** | **2.61E-08** |
| Brown fat weight | **2.73E-10** | 0.459 |
| Skeletal muscle weight | **2.70E-04** | 0.346 |
| Serum leptin | **5.27E-07** | **2.38E-08** |
| Serum insulin | **1.00E-05** | **5.00E-05** |
| Serum triglycerides | **0.015** | **1.70E-04** |
| Serum cholesterol | 0.097 | 0.591 |
| Serum glucose | **1.91E-06** | **6.41E-03** |
| Serum free fatty acids | 0.054 | 0.641 |

**Table B. Averages of weekly weights, glucose and insulin tolerance values, and organ weights in sons.** HF = High-fat diet, LF = Low-fat diet, maternal diet listed before hyphen, offspring diet listed after hyphen.

| **Trait** | **HF-HF ♂** | **LF-HF ♂** | **HF-LF ♂** | **LF-LF ♂** |
| --- | --- | --- | --- | --- |
| **Wk 1 weight (g)** | 3.65 ± 0.22 | 3.61 ± 0.12 | 3.53 ± 0.21 | 3.58 ± 0.18 |
| **Wk 2 weight (g)** | 5.98 ± 0.30 | 5.75 ± 0.22 | 5.99 ± 0.29 | 5.82 ± 0.25 |
| **Wk 3 weight (g)** | 7.41 ± 0.35 | 7.14 ± 0.48 | 7.81 ± 0.29 | 7.55 ± 0.24 |
| **Wk 4 weight (g)** | 14.25 ± 0.60 | 13.54 ± 0.48 | 11.98 ± 0.31 | 11.39 ± 0.39 |
| **Wk 5 weight (g)** | 16.89 ± 0.42 | 17.18 ± 0.31 | 13.76 ± 0.45 | 13.14 ± 0.30 |
| **Wk 6 weight (g)** | 17.19 ± 0.41 | 17.13 ± 0.61 | 13.52 ± 0.48 | 13.27 ± 0.23 |
| **Wk 7 weight (g)** | 18.57 ± 0.37 | 17.98 ± 0.52 | 14.06 ± 0.37 | 14.12 ± 0.31 |
| **Wk 8 weight (g)** | 19.74 ± 0.37 | 19.31 ± 0.55 | 14.71 ± 0.39 | 14.77 ± 0.37 |
| **Wk 9 weight (g)** | 20.72 ± 1.19 | 19.28 ± 1.77 | 15.38 ± 0.36 | 15.38 ± 0.28 |
| **Wk 10 weight (g)** | 23.64 ± 0.51 | 22.74 ± 0.65 | 15.93 ± 0.46 | 16.13 ± 0.30 |
| **Wk 11 weight (g)** | 24.76 ± 0.79 | 24.56 ± 0.81 | 16.76 ± 0.46 | 16.68 ± 0.29 |
| **Wk 12 weight (g)** | 27.57 ± 0.80 | 26.95 ± 1.02 | 17.56 ± 0.37 | 17.10 ± 0.30 |
| **Wk 13 weight (g)** | 29.70 ± 1.02 | 28.53 ± 1.14 | 18.10 ± 0.48 | 17.66 ± 0.33 |
| **Wk 14 weight (g)** | 31.96 ± 1.06 | 29.29 ± 1.47 | 18.75 ± 0.41 | 18.44 ± 0.34 |
| **Wk 15 weight (g)** | 33.00 ± 1.02 | 32.47 ± 1.12 | 18.96 ± 0.38 | 18.90 ± 0.34 |
| **Baseline glucose in GTT** | 225.80 ± 6.76 | 223.08 ± 10.59 | 146.50 ± 9.54 | 150.54 ± 8.28 |
| **IPGTT AUC** | 38608.50 ± 2526.95 | 37821.25 ± 1734.10 | 19473.00 ± 882.46 | 20307.69 ± 976.06 |
| **Wk 16 weight (g)** | 33.59 ± 1.04 | 33.64 ± 0.93 | 19.27 ± 0.43 | 19.25 ± 0.35 |
| **Baseline glucose in IPITT** | 214.60 ± 11.72 | 214.80 ± 11.93 | 142.60 ± 10.88 | 140.50 ± 8.41 |
| **IPITT AUC** | 26622.00 ± 1582.54 | 26028.00 ± 1201.84 | 12792.00 ± 1605.38 | 11831.25 ± 780.32 |
| **Wk 17 weight (g)** | 33.09 ± 0.74 | 34.56 ± 1.17 | 18.92 ± 0.49 | 19.66 ± 0.35 |
| **Liver weight (g)** | 1.83 ± 0.10 | 1.69 ± 0.27 | 0.58 ± 0.06 | 0.66 ± 0.05 |
| **Fat Pad weight (g)** | 1.67 ± 0.10 | 1.84 ± 0.23 | 0.29 ± 0.06 | 0.30 ± 0.04 |
| **Heart weight (g)** | 0.25 ± 0.02 | 0.25 ± 0.02 | 0.16 ± 0.01 | 0.16 ± 0.01 |
| **Left kidney weight (g)** | 0.35 ± 0.02 | 0.36 ± 0.03 | 0.23 ± 0.01 | 0.24 ± 0.01 |
| **Right kidney weight (g)** | 0.37 ± 0.01 | 0.39 ± 0.04 | 0.24 ± 0.01 | 0.26 ± 0.01 |
| **Spleen weight (g)** | 0.13 ± 0.01 | 0.12 ± 0.01 | 0.06 ± 0.01 | 0.06 ± 0.01 |
| **Brown fat weight (g)** | 0.44 ± 0.04 | 0.39 ± 0.06 | 0.13 ± 0.02 | 1.64 ± 1.54 |
| **Leg musl weight (g)** | 0.17 ± 0.03 | 0.17 ± 0.02 | 0.12 ± 0.01 | 1.20 ± 1.08 |
| **Food consumption (g/day)** | 5.13 ± 0.13 | 5.85 ± 0.63 | 3.73 ± 0.10 | 3.66 ± 0.09 |

**Table C. Averages of weekly weights, glucose and insulin tolerance values, and organ weights in daughters.** HF = High-fat diet, LF = Low-fat diet, maternal diet listed before hyphen, offspring diet listed after hyphen.

| **Trait** | HF-LF ♀ | LF-LF ♀ | HF-HF ♀ | LF-HF ♀ |
| --- | --- | --- | --- | --- |
| **Wk 1 weight (g)** | 3.81 ± 0.23 | 3.52 ± 0.15 | 3.74 ± 0.16 | 3.48 ± 0.19 |
| **Wk 2 weight (g)** | 5.56 ± 0.23 | 5.63 ± 0.18 | 5.66 ± 0.22 | 5.78 ± 0.24 |
| **Wk 3 weight (g)** | 7.07 ± 0.27 | 7.25 ± 0.21 | 7.25 ± 0.25 | 7.21 ± 0.24 |
| **Wk 4 weight (g)** | 10.34 ± 0.16 | 9.92 ± 0.23 | 12.34 ± 0.25 | 11.48 ± 0.43 |
| **Wk 5 weight (g)** | 11.34 ± 0.17 | 11.05 ± 0.23 | 13.91 ± 0.18 | 13.43 ± 0.34 |
| **Wk 6 weight (g)** | 11.32 ± 0.26 | 11.19 ± 0.27 | 14.47 ± 0.38 | 13.90 ± 0.39 |
| **Wk 7 weight (g)** | 11.66 ± 0.32 | 11.47 ± 0.28 | 15.46 ± 0.38 | 14.40 ± 0.41 |
| **Wk 8 weight (g)** | 11.21 ± 0.91 | 11.59 ± 0.28 | 16.43 ± 0.35 | 15.20 ± 0.47 |
| **Wk 9 weight (g)** | 12.26 ± 0.32 | 11.06 ± 0.69 | 17.57 ± 0.31 | 16.26 ± 0.51 |
| **Wk 10 weight (g)** | 12.63 ± 0.31 | 12.25 ± 0.33 | 19.35 ± 0.42 | 17.56 ± 0.53 |
| **Wk 11 weight (g)** | 12.97 ± 0.31 | 12.68 ± 0.40 | 21.21 ± 0.77 | 18.65 ± 0.60 |
| **Wk 12 weight (g)** | 12.58 ± 0.75 | 13.09 ± 0.32 | 22.98 ± 0.93 | 20.11 ± 0.78 |
| **Wk 13 weight (g)** | 13.80 ± 0.32 | 13.61 ± 0.36 | 24.73 ± 1.11 | 20.13 ± 1.06 |
| **Wk 14 weight (g)** | 14.23 ± 0.32 | 13.94 ± 0.34 | 25.88 ± 1.18 | 22.42 ± 0.92 |
| **Wk 15 weight (g)** | 14.75 ± 0.40 | 14.55 ± 0.32 | 28.15 ± 1.30 | 23.55 ± 0.95 |
| **Baseline glucose in GTT** | 132.79 ± 5.72 | 124.07 ± 6.10 | 171.00 ± 3.59 | 162.17 ± 5.55 |
| **IPGTT AUC** | 16570.71 ± 500.95 | 17453.00 ± 1150.96 | 22841.00 ± 652.08 | 21173.75 ± 454.54 |
| **Wk 16 weight (g)** | 15.97 ± 1.41 | 14.81 ± 0.30 | 27.82 ± 1.61 | 25.20 ± 0.85 |
| **Baseline glucose in IPITT** | 125.07 ± 4.88 | 136.23 ± 4.20 | 163.40 ± 6.13 | 165.58 ± 3.05 |
| **IPITT AUC** | 10616.79 ± 936.99 | 10508.08 ± 641.58 | 18074.00 ± 898.87 | 18356.25 ± 645.94 |
| **Wk 17 weight (g)** | 14.86 ± 0.36 | 15.44 ± 0.32 | 29.57 ± 1.28 | 25.87 ± 0.93 |
| **Liver weight (g)** | 0.54 ± 0.08 | 0.56 ± 0.02 | 1.36 ± 0.16 | 0.95 ± 0.12 |
| **Fat Pad weight (g)** | 0.19 ± 0.10 | 0.14 ± 0.02 | 1.25 ± 0.13 | 0.91 ± 0.10 |
| **Heart weight (g)** | 0.12 ± 0.01 | 0.13 ± 0.01 | 0.19 ± 0.01 | 0.19 ± 0.01 |
| **Left kidney weight (g)** | 0.14 ± 0.01 | 0.14 ± 0.01 | 0.20 ± 0.01 | 0.18 ± 0.01 |
| **Right kidney weight (g)** | 0.15 ± 0.01 | 0.14 ± 0.01 | 0.20 ± 0.01 | 0.18 ± 0.01 |
| **Spleen weight (g)** | 0.05 ± 0.01 | 0.06 ± 0.01 | 0.12 ± 0.01 | 0.11 ± 0.01 |
| **Brown fat weight (g)** | 0.10 ± 0.02 | 0.10 ± 0.01 | 0.56 ± 0.07 | 0.32 ± 0.03 |
| **Leg musl weight (g)** | 0.11 ± 0.01 | 0.10 ± 0.01 | 0.17 ± 0.01 | 0.13 ± 0.01 |
| **Food consumption (g/day)** | 3.24 ± 0.09 | 3.46 ± 0.10 | 5.94 ± 1.15 | 4.47 ± 0.10 |

**Table D. Description of bone length measurements.** Maternal diet did not affect the long-bone lengths or weights, but offspring diet and sex had a significant effect. All repeatabilities were above 0.92. HF = High-fat diet, LF = Low-fat diet, N = 10 per group.

| **Measurement** | **Description** | **Repeatability** | **Sex** | **Offspring diet** |
| --- | --- | --- | --- | --- |
| Humerus Length 1 | Humeral head to outermost edge of the trochlea | 0.9984 | **2.42E-13** | **4.37E-07** |
| Humerus Length 2 | Humeral head to outermost edge of the capitulum | 0.9995 | **1.32E-13** | **1.16E-07** |
| Humerus Weight | Weight of humerus | 0.9861 | **8.07E-10** | **2.85E-10** |
| Ulna Length | Topmost edge of olecranon to tip of styloid process | 0.9954 | **2.63E-14** | **8.06E-07** |
| Ulna Weight | Weight of ulna | 0.9861 | 0.06 | 0.98 |
| Femur Length 1 | Head of femur to median condyle | 0.9993 | **2.90E-04** | **3.76E-07** |
| Femur Length 2 | Tip of the greater trochanter to tip of lateral condyle | 0.9992 | **7.00E-05** | **1.74E-07** |
| Femur Condyle Width | Tip of medial condyle to tip of lateral condyle | 0.9202 | **6.70E-07** | 0.42 |
| Femur Weight | Weight of femur | 0.9963 | **2.07E-07** | **2.51E-12** |
| Tibia Length | Tip of medial condyle to the lateral edge of the medial malleolus | 0.9988 | **2.42E-07** | **7.30E-09** |
| Tibia and Fibula Weight | Weight of tibia and fibula | 0.9907 | **1.96E-09** | **2.42E-12** |

**Table E. Bone length averages.** High-fat mice had longer, heavier bones than low-fat mice, and males had longer, heavier bones than females. Maternal diet did not affect the bone measurements. HF = High-fat diet, LF = Low-fat diet, averages are reported ± one standard deviation unit, N = 10 per group.

| **Diet** | **Humerus Length 1** | **Humerus Length 2** | **Humerus Weight** | **Ulna Length** | **Ulna Weight** | **Femur Length 1** |
| --- | --- | --- | --- | --- | --- | --- |
| HF ♀ | 10.697 ± 1.04 | 10.778 ± 1.13 | 0.015 ± 0.79 | 12.497 ± 0.98 | 0.011 ± 0.14 | 13.911 ± 1.63 |
| LF ♀ | 10.394 ± 1.04 | 10.463 ± 1.13 | 0.014 ± 0.79 | 12.249 ± 0.98 | 0.010 ± 0.14 | 13.253 ± 1.63 |
| HF ♂ | 11.465 ± 1.74 | 11.521 ± 1.81 | 0.018 ± 2.37 | 13.197 ± 1.71 | 0.013 ± 0.14 | 14.196 ± 1.16 |
| LF ♂ | 10.957 ± 1.74 | 11.013 ± 1.81 | 0.015 ± 2.37 | 12.766 ± 1.71 | 0.014 ± 0.14 | 13.729 ± 1.16 |

| **Diet** | **Femur Length 2** | **Femur Condyle Width** | **Femur Weight** | **Tibia Length** | **Tibia and Fibula Weight** | **Sample Size** |
| --- | --- | --- | --- | --- | --- | --- |
| HF ♀ | 14.260 ± 1.67 | 2.275 ± 0.07 | 0.030 ± 1.58 | 15.657 ± 1.64 | 0.026 ± 1.92 | 16 |
| LF ♀ | 13.536 ± 1.67 | 2.271 ± 0.07 | 0.025 ± 1.58 | 15.045 ± 1.64 | 0.022 ± 1.92 | 19 |
| HF ♂ | 14.615 ± 1.22 | 2.363 ± 0.38 | 0.035 ± 2.21 | 16.187 ± 1.64 | 0.030 ± 2.41 | 14 |
| LF ♂ | 14.086 ± 1.22 | 2.344 ± 0.38 | 0.028 ± 2.21 | 15.576 ± 1.64 | 0.025 ± 2.41 | 18 |

**Table F. Differentially expressed genes due to maternal diet for high-fat-fed daughters (liver).**

| **Gene Name** | **logFC** | **P-value** | **Expression**  **HF-HF** | **Expression**  **LF-HF** | **SE HF-HF** | **SE LF-HF** | **Known Disease Involvement** |
| --- | --- | --- | --- | --- | --- | --- | --- |
| *Lmo1* | 3.065 | 3.24E-04 | -0.34 | -3.38 | 0.86 | 0.77 |  |
| *Mpo* | 2.790 | 2.47E-04 | -0.65 | -3.41 | 0.62 | 1.01 | Obesity, Diabetes, CVD |
| *Sult2a3* | 2.783 | 8.23E-03 | 1.78 | -1.21 | 1.03 | 0.74 |  |
| *Lgr6* | 2.739 | 3.05E-04 | -2.79 | -5.56 | 0.41 | 0.53 |  |
| *Ngp* | 2.686 | 5.02E-03 | 0.19 | -2.42 | 0.88 | 0.94 |  |
| *Sult3a2* | 2.655 | 4.99E-05 | 4.07 | 1.29 | 0.73 | 0.30 |  |
| *Gm5210* | 2.504 | 2.80E-04 | -2.95 | -5.39 | 0.33 | 0.27 |  |
| *Srpk3* | -2.482 | 6.75E-05 | -4.61 | -2.14 | 0.53 | 0.29 |  |
| *A4gnt* | 2.471 | 1.79E-02 | -0.20 | -2.55 | 0.70 | 0.69 |  |
| *Slc9b1* | 2.457 | 3.54E-04 | -3.51 | -6.03 | 0.30 | 0.20 |  |
| *Camp* | 2.455 | 7.65E-03 | -2.57 | -4.89 | 1.05 | 0.83 |  |
| *Gm15540* | -2.355 | 4.87E-04 | -5.19 | -2.93 | 0.22 | 0.21 |  |
| *Gm17022* | -2.344 | 3.16E-04 | -5.00 | -2.69 | 0.40 | 0.28 |  |
| *Aplnr* | 2.329 | 2.17E-04 | -1.81 | -4.07 | 0.17 | 0.66 |  |
| *Ltf* | 2.329 | 2.54E-02 | -1.05 | -3.30 | 0.76 | 0.95 | Obesity, Diabetes, CVD |
| *Gm26937* | 2.312 | 2.15E-03 | -3.42 | -5.71 | 0.78 | 0.17 |  |
| *Adam1a* | -2.292 | 6.94E-05 | -5.00 | -2.71 | 0.40 | 0.32 |  |
| *Kat6b-ps2* | 2.288 | 1.01E-04 | -2.09 | -4.46 | 0.43 | 0.48 |  |
| *Gm17229* | 2.274 | 2.64E-03 | -3.32 | -5.71 | 0.30 | 0.39 |  |
| *Sult3a1* | 2.269 | 2.39E-05 | 6.69 | 4.32 | 0.55 | 0.43 |  |
| *Cd177* | 2.251 | 1.12E-03 | -1.91 | -4.09 | 0.42 | 0.69 |  |
| *Gm16731* | -2.244 | 8.13E-04 | -4.61 | -2.28 | 0.68 | 0.11 |  |
| *Il13ra2* | -2.242 | 2.14E-04 | -5.19 | -3.06 | 0.22 | 0.41 |  |
| *Igkv4-74* | 2.236 | 4.11E-03 | -2.29 | -4.61 | 0.67 | 0.58 |  |
| *2610507I01Rik* | -2.229 | 7.10E-04 | -4.79 | -2.52 | 0.36 | 0.21 |  |
| *Gm23388* | 2.210 | 1.22E-03 | -2.41 | -4.61 | 0.18 | 0.51 |  |
| *Pla2g4f* | 2.196 | 5.01E-03 | -0.94 | -3.12 | 0.93 | 0.78 |  |
| *Hao2* | 2.143 | 1.05E-04 | 3.06 | 0.81 | 0.60 | 0.28 |  |
| *Gm23935* | 2.141 | 3.89E-04 | 9.02 | 6.85 | 0.13 | 0.19 |  |
| *Ltk* | -2.140 | 2.18E-04 | -4.79 | -2.50 | 0.59 | 0.14 |  |
| *Gm15344* | -2.135 | 3.30E-03 | -5.19 | -2.76 | 0.48 | 0.62 |  |
| *Mcmdc2* | 2.110 | 2.42E-03 | -1.98 | -3.99 | 0.14 | 0.72 |  |
| *Pcdh11x* | 2.107 | 1.71E-03 | -1.91 | -4.05 | 0.25 | 0.55 |  |
| *Cap2* | 2.107 | 8.12E-03 | -3.46 | -5.71 | 0.93 | 0.40 |  |
| *5430416N02Rik* | -2.103 | 3.30E-04 | -3.90 | -1.82 | 0.86 | 0.21 |  |
| *Gm4956* | -2.102 | 4.36E-04 | -2.32 | -0.12 | 0.28 | 0.42 |  |
| *Gm4419* | -2.081 | 3.13E-03 | -5.58 | -3.43 | 0.18 | 0.31 |  |
| *Mybl2* | -2.070 | 2.61E-04 | -4.61 | -2.60 | 0.45 | 0.35 |  |
| *mt-Rnr2* | 2.054 | 3.45E-04 | 11.83 | 9.75 | 0.18 | 0.34 |  |
| *Tmem167-ps1* | 2.043 | 1.81E-03 | -1.62 | -3.72 | 0.65 | 0.51 |  |
| *Gm20125* | 2.038 | 2.44E-03 | -2.93 | -4.83 | 0.32 | 0.38 |  |
| *Mir6236* | 2.035 | 1.04E-03 | 10.84 | 8.78 | 0.14 | 0.26 |  |
| *Gm28323* | -2.030 | 1.65E-03 | -5.19 | -3.17 | 0.22 | 0.37 |  |
| *Gm16172* | -2.027 | 4.64E-03 | -4.61 | -2.62 | 0.45 | 0.37 |  |
| *Chrna4* | 2.027 | 2.33E-02 | -0.84 | -2.73 | 0.21 | 0.75 |  |
| *Erbb4* | 2.006 | 6.11E-06 | 0.52 | -1.49 | 0.46 | 0.15 | Obesity, Diabetes, CVD |

**Table G. Differentially expressed genes due to maternal diet for high-fat-fed daughters (heart).**

| **Gene Name** | **logFC** | **P-value** | **Expression HF-HF** | **Expression LF-HF** | **SE HF-HF** | **SE LF-HF** | **Known Disease Involvement** |
| --- | --- | --- | --- | --- | --- | --- | --- |
| *Hmgb1-rs16* | 2.510 | 2.38E-06 | -2.89 | -5.40 | 0.17 | 0.06 |  |
| *Rassf6* | -2.807 | 3.17E-06 | -5.48 | -2.67 | 0.04 | 0.33 |  |
| *Mab21l3* | -2.106 | 2.30E-05 | -5.48 | -3.37 | 0.04 | 0.20 |  |
| *Prok1* | -2.106 | 2.55E-05 | -5.48 | -3.37 | 0.04 | 0.21 | Diabetes, CVD |
| *Paqr5* | 2.436 | 3.70E-05 | -1.54 | -3.98 | 0.18 | 0.41 |  |
| *Gm25363* | 2.292 | 4.23E-05 | -2.79 | -5.08 | 0.14 | 0.31 |  |
| *Gm16206* | 2.190 | 1.05E-04 | -2.89 | -5.08 | 0.19 | 0.32 |  |
| *4930533K18Rik* | 2.029 | 1.52E-04 | -3.37 | -5.40 | 0.35 | 0.06 |  |
| *Gm8145* | 2.371 | 2.49E-04 | -2.25 | -4.62 | 0.18 | 0.52 |  |
| *B430305J03Rik* | 1.974 | 2.96E-04 | -3.11 | -5.08 | 0.22 | 0.31 |  |
| *Mal2* | -2.699 | 3.38E-04 | -4.03 | -1.32 | 0.76 | 0.19 |  |
| *Rec114* | 2.483 | 3.40E-04 | -2.13 | -4.62 | 0.35 | 0.51 |  |
| *4930563E18Rik* | 2.101 | 3.89E-04 | -2.98 | -5.08 | 0.33 | 0.32 |  |
| *Gm8428* | 2.132 | 4.43E-04 | -2.64 | -4.77 | 0.33 | 0.36 |  |
| *Gm20517* | 2.438 | 7.29E-04 | -1.21 | -3.64 | 0.21 | 0.75 |  |
| *Gm28379* | 2.240 | 7.67E-04 | -2.60 | -4.84 | 0.26 | 0.53 |  |
| *Slc17a4* | -2.333 | 8.04E-04 | -4.06 | -1.74 | 0.67 | 0.21 | CVD |
| *Gm16192* | 1.973 | 8.09E-04 | -2.35 | -4.30 | 0.24 | 0.43 |  |
| *Tjp3* | -1.984 | 8.43E-04 | -3.75 | -1.76 | 0.55 | 0.09 |  |
| *Cyct* | 3.146 | 1.16E-03 | -0.75 | -3.89 | 0.26 | 1.15 |  |
| *Gm11737* | 2.112 | 1.25E-03 | -2.18 | -4.30 | 0.34 | 0.50 |  |
| *Plin1* | -2.198 | 1.32E-03 | -4.14 | -1.95 | 0.58 | 0.33 | Obesity, Diabetes |
| *Cyp2c54* | -2.332 | 1.40E-03 | -2.55 | -0.25 | 0.83 | 0.21 |  |
| *Gm12396* | -1.959 | 1.47E-03 | -5.48 | -3.52 | 0.04 | 0.56 |  |
| *Kynu* | -2.334 | 1.47E-03 | -3.26 | -0.94 | 0.67 | 0.36 | CVD |
| *Cyp2c67* | -2.077 | 1.59E-03 | -2.61 | -0.58 | 0.47 | 0.39 |  |
| *Cyp2f2* | -1.983 | 1.62E-03 | -0.25 | 1.66 | 0.57 | 0.36 |  |
| *Fgfr4* | -2.121 | 1.67E-03 | -3.49 | -1.37 | 0.62 | 0.30 | CVD |
| *Gm14212* | 1.971 | 1.93E-03 | -2.48 | -4.45 | 0.44 | 0.38 |  |
| *Cyp2c44* | -2.494 | 2.09E-03 | -1.83 | 0.61 | 0.48 | 0.52 |  |
| *Gm28809* | 1.984 | 2.36E-03 | -2.21 | -4.20 | 0.23 | 0.59 |  |
| *Gm15869* | 1.977 | 2.37E-03 | -2.78 | -4.77 | 0.43 | 0.42 |  |
| *Tulp2* | 1.962 | 2.52E-03 | -2.51 | -4.47 | 0.22 | 0.57 |  |
| *F11* | -2.163 | 3.06E-03 | -3.72 | -1.58 | 0.76 | 0.27 | Diabetes, CVD |
| *Inhbc* | -1.986 | 3.81E-03 | -3.32 | -1.34 | 0.69 | 0.29 | Obesity, CVD |
| *Prss35* | -2.715 | 3.87E-03 | -5.48 | -2.76 | 0.04 | 1.04 |  |
| *Asmt* | 2.037 | 5.86E-03 | -1.48 | -3.50 | 0.21 | 0.83 |  |
| *Gm4952* | -1.961 | 6.47E-03 | -3.02 | -1.06 | 0.77 | 0.30 |  |
| *Prdm6* | -2.170 | 6.66E-03 | -4.79 | -2.61 | 0.71 | 0.45 | CVD |
| *Cd5l* | -1.972 | 1.34E-02 | -2.81 | -0.87 | 0.86 | 0.40 | CVD |
| *Clec3a* | -2.255 | 1.41E-02 | -5.48 | -3.22 | 0.04 | 1.07 |  |
| *Gm16270* | 2.006 | 1.46E-02 | -2.54 | -4.52 | 0.73 | 0.54 |  |
| *Cyp4a12a* | -2.099 | 1.62E-02 | -4.38 | -2.26 | 0.50 | 0.82 |  |
| *Cyp2c50* | -2.063 | 1.65E-02 | -1.62 | 0.36 | 0.97 | 0.44 |  |
| *Sln* | -3.061 | 2.38E-02 | -4.15 | -1.05 | 0.78 | 1.21 | Diabetes, CVD |

**Table H. Differentially expressed genes due to maternal diet for low-fat-fed daughters.**

| **Gene Name** | **logFC** | **P-value** | **Expression HF-LF** | **Expression LF-LF** | **SE HF-LF** | **SE LF-LF** | **Known Disease Involvement** |
| --- | --- | --- | --- | --- | --- | --- | --- |
| *Dmbt1* | 3.529 | 4.21E-02 | 1.04 | -2.09 | 0.97 | 0.96 |  |
| *Gm11454* | -3.475 | 2.98E-07 | -4.41 | -0.96 | 0.44 | 0.53 | Obesity, Diabetes, CVD |
| *Sftpa1* | 3.256 | 8.08E-05 | -1.42 | -4.54 | 0.67 | 0.51 |  |
| *Gucy2e* | 3.108 | 1.10E-05 | -1.83 | -5.15 | 0.21 | 0.58 |  |
| *Hmmr* | -3.081 | 2.36E-04 | -4.04 | -0.82 | 0.63 | 0.54 |  |
| *Cdc20* | -3.054 | 4.90E-05 | -2.94 | 0.14 | 0.52 | 0.23 |  |
| *Pdk4* | 3.023 | 3.63E-05 | 5.37 | 2.40 | 0.53 | 0.18 |  |
| *Rpl3l* | 2.940 | 1.57E-05 | -2.54 | -5.55 | 0.52 | 0.35 |  |
| *Arhgap8* | 2.920 | 2.60E-06 | -2.90 | -5.95 | 0.37 | 0.31 |  |
| *Zfp811* | 2.835 | 4.81E-05 | -3.01 | -5.95 | 0.34 | 0.31 |  |
| *Lockd* | -2.772 | 4.49E-06 | -5.20 | -2.34 | 0.23 | 0.36 |  |
| *Trpv3* | 2.768 | 1.11E-04 | -3.12 | -5.95 | 0.25 | 0.31 |  |
| *Ppp1r3g* | 2.720 | 2.84E-04 | 5.68 | 3.03 | 0.55 | 0.76 |  |
| *Vpreb3* | 2.707 | 2.22E-05 | 1.18 | -1.54 | 0.29 | 0.56 | Obesity, CVD |
| *Cytl1* | 2.706 | 3.66E-04 | -2.33 | -5.15 | 0.25 | 0.54 | Obesity, Diabetes, CVD |
| *Gm28644* | 2.659 | 3.76E-04 | -3.17 | -5.95 | 0.33 | 0.31 |  |
| *Dsg1a* | 2.633 | 6.76E-06 | -2.28 | -4.97 | 0.17 | 0.57 |  |
| *Cdkn3* | -2.617 | 7.59E-06 | -4.73 | -2.08 | 0.47 | 0.39 |  |
| *Gcsam* | 2.614 | 4.63E-05 | -3.24 | -5.95 | 0.22 | 0.31 |  |
| *Ccnb2* | -2.611 | 3.54E-03 | -3.32 | -0.48 | 0.73 | 0.64 |  |
| *Gngt1* | -2.608 | 1.90E-05 | -4.24 | -1.62 | 0.31 | 0.27 |  |
| *Cenpf* | -2.575 | 2.30E-04 | -3.63 | -0.98 | 0.56 | 0.33 |  |
| *Gm10032* | 2.571 | 6.68E-04 | -3.29 | -5.95 | 0.58 | 0.31 | CVD |
| *Kifc1* | -2.559 | 5.06E-05 | -4.88 | -2.28 | 0.41 | 0.39 |  |
| *Rapgef4os2* | 2.465 | 2.52E-04 | -0.77 | -3.02 | 0.12 | 0.67 |  |
| *Gm10787* | 2.414 | 2.94E-04 | -2.53 | -5.15 | 0.51 | 0.58 |  |
| *Fam69b* | 2.362 | 9.33E-05 | -2.67 | -4.97 | 0.27 | 0.31 | Diabetes, CVD |
| *Mmrn1* | 2.361 | 3.67E-04 | -2.48 | -4.97 | 0.47 | 0.64 |  |
| *Gm11832* | -2.309 | 3.48E-06 | -2.66 | -0.38 | 0.39 | 0.42 |  |
| *Egfros* | 2.306 | 2.35E-02 | -1.39 | -3.39 | 0.58 | 1.38 |  |
| *Hapln1* | -2.294 | 2.15E-03 | -1.43 | 0.60 | 0.18 | 0.82 |  |
| *Gm26744* | 2.271 | 9.07E-04 | -2.77 | -5.37 | 0.30 | 0.78 |  |
| *Ppbp* | 2.256 | 1.88E-03 | -1.75 | -4.06 | 0.31 | 0.87 |  |
| *Pnpla5* | -2.246 | 2.55E-06 | -1.56 | 0.71 | 0.48 | 0.34 |  |
| *Ckap2* | -2.244 | 1.12E-03 | -3.57 | -1.13 | 0.66 | 0.55 |  |
| *Sytl3* | 2.239 | 1.31E-03 | -3.63 | -5.95 | 0.16 | 0.31 |  |
| *Spdye4c* | 2.223 | 4.18E-04 | -3.63 | -5.95 | 0.50 | 0.31 |  |
| *Rmi2* | -2.210 | 1.67E-03 | -4.73 | -2.61 | 0.39 | 0.25 |  |
| *Rasd1* | 2.206 | 4.73E-04 | -0.95 | -3.18 | 0.47 | 0.25 |  |
| *Osbpl3* | 2.202 | 3.70E-03 | 1.95 | -0.08 | 0.44 | 0.64 |  |
| *Snora78* | 2.202 | 2.48E-03 | -2.84 | -4.97 | 0.38 | 0.29 |  |
| *A530013C23Rik* | 2.201 | 6.79E-04 | -1.05 | -3.37 | 0.57 | 0.73 |  |
| *Atp2b2* | -2.194 | 4.42E-06 | 0.47 | 2.71 | 0.34 | 0.27 | Obesity, CVD |
| *Gm12186* | 2.194 | 1.19E-03 | -3.68 | -5.95 | 0.30 | 0.31 | Obesity, Diabetes, CVD |
| *Meox1* | -2.193 | 1.76E-04 | -3.80 | -1.57 | 0.47 | 0.11 |  |
| *4930509G22Rik* | 2.185 | 5.85E-04 | -2.91 | -4.97 | 0.19 | 0.29 |  |
| *Dgki* | 2.179 | 2.28E-04 | -3.68 | -5.95 | 0.24 | 0.31 |  |
| *Gm11914* | 2.125 | 1.95E-03 | -3.17 | -5.24 | 0.24 | 0.56 |  |
| *Klhl32* | 2.124 | 1.36E-03 | -3.01 | -5.02 | 0.26 | 0.77 |  |
| *Gm12353* | 2.119 | 3.98E-03 | -3.39 | -5.55 | 0.29 | 0.35 |  |
| *Gm20404* | 2.110 | 4.73E-03 | -3.17 | -5.55 | 0.34 | 0.61 |  |
| *Gm12735* | 2.103 | 1.87E-03 | -2.99 | -4.97 | 0.34 | 0.31 |  |
| *Acot5* | 2.100 | 2.16E-04 | 1.55 | -0.55 | 0.19 | 0.50 |  |
| *Gm17597* | 2.095 | 6.66E-04 | -2.80 | -4.79 | 0.33 | 0.37 |  |
| *Gm15785* | 2.091 | 5.56E-04 | -3.78 | -5.95 | 0.16 | 0.31 |  |
| *Arntl* | -2.076 | 3.40E-04 | -0.31 | 1.82 | 0.36 | 0.49 |  |
| *Pnpla3* | -2.067 | 3.22E-04 | 1.98 | 4.04 | 0.17 | 0.59 |  |
| *Ighv3-1* | 2.066 | 9.25E-04 | -3.85 | -5.95 | 0.57 | 0.31 |  |
| *Gm13855* | -2.062 | 8.22E-06 | -2.02 | -0.03 | 0.33 | 0.24 |  |
| *Apitd1* | -2.058 | 4.20E-04 | -3.56 | -1.42 | 0.46 | 0.21 |  |
| *Gm16291* | 2.043 | 6.41E-04 | -0.45 | -2.31 | 0.12 | 0.69 |  |
| *2310040G07Rik* | 2.031 | 6.28E-03 | -3.49 | -5.55 | 0.25 | 0.32 |  |
| *Arhgef39* | -2.029 | 8.15E-04 | -4.24 | -2.08 | 0.63 | 0.38 |  |
| *Lrrtm3* | 2.028 | 2.18E-03 | -3.49 | -5.55 | 0.21 | 0.35 |  |
| *Sag* | 2.023 | 4.54E-04 | -3.85 | -5.95 | 0.47 | 0.31 |  |
| *Gm11205* | 2.018 | 3.84E-03 | -3.49 | -5.55 | 0.25 | 0.35 |  |
| *Ephx4* | 2.014 | 2.63E-03 | -3.85 | -5.95 | 0.45 | 0.31 |  |
| *Gm6114* | -2.006 | 2.40E-03 | -5.20 | -3.11 | 0.40 | 0.21 |  |
| *Scn3b* | 2.005 | 4.95E-03 | -3.39 | -5.37 | 0.15 | 0.46 |  |
| *Unc79* | 2.005 | 2.39E-04 | 1.97 | -0.06 | 0.30 | 0.65 |  |

**Table I. Differentially expressed genes due to maternal diet for high-fat-fed sons.**

| **Gene Name** | **logFC** | **P-value** | **Expression HF-HF** | **Expression LF-HF** | **SE HF-HF** | **SE LF-HF** | **Known Disease Involvement** |
| --- | --- | --- | --- | --- | --- | --- | --- |
| *Myh6* | 2.679 | 0.0420 | -3.23 | -6.05 | 1.37 | 0.32 | Diabetes, CVD |
| *Unc79* | 2.590 | 0.0166 | -1.38 | -3.79 | 0.41 | 1.04 |  |
| *BC043934* | 2.468 | 0.0004 | -3.00 | -5.34 | 0.28 | 0.47 |  |
| *Scarna13* | 2.450 | 0.0009 | -2.77 | -5.07 | 0.22 | 0.73 |  |
| *Hsd17b1* | 2.445 | 0.0002 | -1.86 | -4.20 | 0.29 | 0.79 | Obesity, Diabetes |
| *Mb* | 2.336 | 0.0200 | -3.63 | -6.05 | 1.13 | 0.32 |  |
| *D830044D21Rik* | 2.327 | 0.0014 | -3.33 | -5.65 | 0.24 | 0.22 |  |
| *E230001N04Rik* | 2.309 | 0.0025 | -1.98 | -4.20 | 0.41 | 0.55 |  |
| *Slc22a29* | -2.225 | 0.0007 | -5.45 | -3.35 | 0.10 | 0.71 |  |
| *Myl2* | 2.191 | 0.0073 | -3.78 | -6.05 | 1.04 | 0.32 | Obesity, Diabetes, CVD |
| *Ryr2* | 2.161 | 0.0048 | -3.49 | -5.65 | 0.67 | 0.22 | Diabetes, CVD |
| *Slc22a27* | -2.153 | 0.0463 | -3.72 | -1.74 | 0.86 | 0.66 |  |
| *Kcne3* | -2.136 | 0.0013 | -4.82 | -2.56 | 0.71 | 0.15 | Diabetes, CVD |
| *Tssk4* | 2.128 | 0.0023 | -2.29 | -4.39 | 0.27 | 0.43 |  |
| *Gm12168* | -2.114 | 0.0002 | -4.35 | -2.25 | 0.45 | 0.19 |  |
| *A430093F15Rik* | 2.098 | 0.0112 | -2.81 | -4.76 | 0.29 | 0.50 |  |
| *4933406C10Rik* | 2.092 | 0.0025 | -3.34 | -5.34 | 0.46 | 0.47 |  |
| *Gprasp2* | -2.092 | 0.0009 | -4.99 | -2.91 | 0.42 | 0.34 |  |
| *Wfdc3* | 2.083 | 0.0021 | -3.08 | -5.07 | 0.24 | 0.28 |  |
| *Pnck* | 2.054 | 0.0081 | -3.11 | -5.07 | 0.27 | 0.28 |  |
| *Rcor2* | 2.046 | 0.0062 | -2.99 | -5.25 | 0.35 | 0.65 |  |
| *Omp* | -2.034 | 0.0001 | -4.99 | -2.91 | 0.44 | 0.32 | Diabetes, CVD |

**Table J. Differentially expressed genes due to maternal diet for low-fat-fed sons.**

| **Gene Name** | **logFC** | **P-value** | **Expression HF-LF** | **Expression LF-LF** | **SE HF-LF** | **SE LF-LF** | **Known Disease Involvement** |
| --- | --- | --- | --- | --- | --- | --- | --- |
| *Mb* | 5.527 | 2.72E-07 | -1.33 | -6.56 | 1.50 | 0.26 |  |
| *Myh6* | 4.375 | 2.53E-03 | -1.30 | -5.19 | 1.95 | 0.38 | Diabetes, CVD |
| *Myl2* | 4.082 | 1.39E-05 | -2.61 | -6.56 | 1.19 | 0.26 | Obesity, Diabetes, CVD |
| *Tnni3* | 3.769 | 3.51E-05 | -2.47 | -6.16 | 0.89 | 0.43 | Obesity, Diabetes, CVD |
| *Xirp2* | 3.560 | 2.10E-06 | -2.95 | -6.56 | 1.02 | 0.26 |  |
| *Ckmt2* | 3.364 | 5.83E-07 | -3.08 | -6.56 | 1.00 | 0.26 |  |
| *Actn2* | 3.297 | 2.53E-06 | -2.85 | -6.16 | 1.05 | 0.43 | Obesity, CVD |
| *Eef1a2* | 3.225 | 1.72E-04 | -2.85 | -5.98 | 1.05 | 0.42 |  |
| *Myh7* | 3.201 | 6.17E-05 | -3.21 | -6.56 | 0.86 | 0.26 | CVD |
| *Gm15473* | 3.097 | 1.08E-07 | -3.42 | -6.56 | 0.52 | 0.26 |  |
| *Mybpc3* | 3.042 | 2.22E-03 | -2.41 | -5.19 | 1.30 | 0.38 | Diabetes, CVD |
| *Gm11991* | 3.007 | 4.05E-06 | -3.21 | -6.16 | 0.31 | 0.26 |  |
| *Tcap* | 2.984 | 4.07E-04 | -2.87 | -5.77 | 1.05 | 0.29 | CVD |
| *Cox8b* | 2.963 | 3.96E-05 | -3.21 | -6.16 | 0.61 | 0.26 |  |
| *Myoz2* | 2.856 | 6.74E-06 | -3.53 | -6.56 | 0.70 | 0.26 | CVD |
| *Actc1* | 2.856 | 4.72E-02 | -1.57 | -3.99 | 1.79 | 0.21 | Diabetes, CVD |
| *Gm6881* | 2.838 | 9.48E-05 | -3.42 | -6.16 | 0.65 | 0.26 |  |
| *Mfap5* | 2.819 | 6.85E-04 | -3.32 | -6.16 | 0.61 | 0.26 | CVD |
| *Ckm* | 2.758 | 1.01E-04 | -3.30 | -6.16 | 0.82 | 0.26 | Diabetes, CVD |
| *Dlk2* | 2.751 | 4.33E-05 | -3.02 | -5.77 | 0.27 | 0.38 |  |
| *Gm24187* | 2.746 | 1.10E-03 | -3.30 | -6.16 | 0.58 | 0.50 |  |
| *Dpysl5* | 2.741 | 3.87E-04 | -3.48 | -6.16 | 0.72 | 0.43 |  |
| *Rad54l* | -2.741 | 1.00E-06 | -4.97 | -2.08 | 0.18 | 0.41 | CVD |
| *Gm25381* | 2.724 | 8.85E-07 | -3.79 | -6.56 | 0.35 | 0.26 |  |
| *Gm7719* | 2.724 | 8.47E-05 | -3.79 | -6.56 | 0.35 | 0.26 |  |
| *Gm5533* | 2.705 | 7.00E-07 | -4.00 | -6.56 | 0.76 | 0.26 |  |
| *Adprhl1* | 2.698 | 1.60E-05 | -3.69 | -6.56 | 0.57 | 0.26 |  |
| *Gm29155* | 2.693 | 5.24E-04 | -3.39 | -6.16 | 0.18 | 0.50 |  |
| *BC100451* | 2.686 | 3.09E-04 | -3.48 | -6.16 | 0.72 | 0.50 |  |
| *Fabp3* | 2.685 | 1.11E-03 | -3.01 | -5.77 | 0.62 | 0.51 | Obesity, Diabetes, CVD |
| *Gm9167* | 2.683 | 1.89E-03 | -2.72 | -5.37 | 0.30 | 0.23 |  |
| *Nppb* | 2.678 | 8.22E-07 | -3.71 | -6.56 | 0.67 | 0.26 | Obesity, Diabetes, CVD |
| *Hsd11b2* | 2.675 | 1.80E-03 | -2.99 | -5.50 | 0.53 | 0.88 | Diabetes, CVD |
| *Ryr2* | 2.669 | 2.09E-03 | -2.89 | -5.58 | 1.05 | 0.63 | Diabetes, CVD |
| *Gm23628* | 2.625 | 1.27E-04 | -3.88 | -6.56 | 0.65 | 0.26 |  |
| *Aox4* | 2.601 | 3.48E-07 | -4.00 | -6.56 | 0.65 | 0.26 |  |
| *Krt222* | 2.595 | 1.16E-03 | -2.90 | -5.40 | 0.39 | 0.48 |  |
| *Gm17059* | 2.585 | 5.81E-05 | -3.81 | -6.56 | 0.49 | 0.26 |  |
| *Gm12717* | 2.582 | 2.63E-04 | -3.39 | -5.86 | 0.18 | 0.54 |  |
| *Gm7803* | 2.569 | 9.97E-06 | -4.00 | -6.56 | 0.63 | 0.26 |  |
| *Cox6a2* | 2.565 | 3.00E-03 | -1.94 | -4.39 | 0.70 | 0.62 |  |
| *Gm6430* | 2.542 | 9.61E-05 | -4.00 | -6.56 | 0.59 | 0.26 |  |
| *Slc9b1* | 2.542 | 9.96E-05 | -4.00 | -6.56 | 0.59 | 0.26 |  |
| *Gm14276* | -2.530 | 1.98E-06 | -4.97 | -2.36 | 0.18 | 0.31 |  |
| *Sgcg* | 2.527 | 2.13E-05 | -3.88 | -6.56 | 0.52 | 0.26 | Diabetes |
| *Platr9* | 2.521 | 1.26E-04 | -4.00 | -6.56 | 0.57 | 0.26 |  |
| *Gm11127* | 2.504 | 1.97E-03 | -3.48 | -5.86 | 0.62 | 0.54 |  |
| *Gm11954* | 2.502 | 2.79E-07 | -4.18 | -6.56 | 0.64 | 0.26 |  |
| *Gm13773* | 2.502 | 2.80E-07 | -4.18 | -6.56 | 0.64 | 0.26 |  |
| *4933402J07Rik* | 2.502 | 2.08E-06 | -4.18 | -6.56 | 0.64 | 0.26 |  |
| *Gm28155* | 2.502 | 1.56E-05 | -4.18 | -6.56 | 0.64 | 0.26 |  |
| *Adcy8* | 2.502 | 2.59E-05 | -4.18 | -6.56 | 0.64 | 0.26 |  |
| *Kcnb2* | 2.502 | 1.43E-04 | -4.18 | -6.56 | 0.64 | 0.26 | CVD |
| *Trim63* | 2.496 | 1.12E-04 | -3.39 | -5.86 | 0.53 | 0.54 | CVD |
| *Gm13094* | 2.483 | 6.88E-04 | -3.60 | -6.16 | 0.41 | 0.43 |  |
| *4930451E10Rik* | 2.483 | 2.96E-04 | -3.60 | -6.16 | 0.41 | 0.43 |  |
| *Sult6b2* | 2.479 | 9.28E-06 | -4.18 | -6.56 | 0.89 | 0.26 |  |
| *Ankrd1* | 2.467 | 2.23E-04 | -1.28 | -3.70 | 0.51 | 0.38 | CVD |
| *H2-Ob* | -2.463 | 3.74E-05 | -4.58 | -2.26 | 0.33 | 0.45 |  |
| *Figf* | -2.456 | 1.30E-04 | -4.27 | -1.96 | 0.62 | 0.45 | Obesity, Diabetes |
| *Gm13472* | 2.423 | 1.23E-04 | -3.81 | -6.16 | 0.71 | 0.26 |  |
| *Ppp1r14c* | 2.417 | 2.11E-05 | -4.00 | -6.56 | 0.42 | 0.26 | CVD |
| *Tlx2* | 2.417 | 7.77E-05 | -4.00 | -6.56 | 0.42 | 0.26 |  |
| *Gm13827* | 2.417 | 1.02E-04 | -4.00 | -6.56 | 0.42 | 0.26 |  |
| *Gm2541* | 2.417 | 5.45E-06 | -4.00 | -6.56 | 0.42 | 0.26 |  |
| *Abra* | 2.417 | 1.03E-04 | -4.00 | -6.56 | 0.42 | 0.26 |  |
| *Gramd2* | 2.417 | 5.66E-04 | -4.00 | -6.56 | 0.42 | 0.26 |  |
| *Fbxo15* | 2.414 | 2.54E-03 | -3.21 | -5.77 | 0.17 | 0.54 |  |
| *Hist1h2ba* | 2.406 | 1.10E-04 | -3.88 | -6.16 | 0.76 | 0.26 |  |
| *Chst4* | 2.401 | 2.26E-03 | -3.60 | -5.86 | 0.56 | 0.54 |  |
| *Ccnb2* | -2.392 | 8.32E-03 | -3.60 | -1.06 | 0.79 | 0.57 |  |
| *Gm15946* | 2.390 | 5.81E-07 | -4.18 | -6.56 | 0.50 | 0.26 |  |
| *Rps29-ps* | 2.390 | 4.26E-06 | -4.18 | -6.56 | 0.50 | 0.26 |  |
| *Csmd2* | 2.390 | 1.97E-05 | -4.18 | -6.56 | 0.50 | 0.26 | Diabetes, CVD |
| *Tmem198* | 2.390 | 7.97E-05 | -4.18 | -6.56 | 0.50 | 0.26 |  |
| *Gm11295* | 2.390 | 2.62E-04 | -4.18 | -6.56 | 0.50 | 0.26 |  |
| *Mcpt8* | 2.389 | 3.47E-06 | -4.18 | -6.56 | 0.50 | 0.26 |  |
| *Gm14323* | 2.389 | 4.31E-06 | -4.18 | -6.56 | 0.50 | 0.26 |  |
| *Sh2d6* | 2.389 | 5.51E-05 | -4.18 | -6.56 | 0.50 | 0.26 |  |
| *1700105P06Rik* | 2.389 | 6.03E-05 | -4.18 | -6.56 | 0.50 | 0.26 |  |
| *4930578C19Rik* | 2.389 | 1.03E-04 | -4.18 | -6.56 | 0.50 | 0.26 |  |
| *Gm7363* | 2.389 | 2.91E-04 | -4.18 | -6.56 | 0.50 | 0.26 |  |
| *Btn1a1* | 2.366 | 1.15E-05 | -4.18 | -6.56 | 0.48 | 0.26 |  |
| *Gm15157* | 2.366 | 2.07E-05 | -4.18 | -6.56 | 0.48 | 0.26 |  |
| *Gm26448* | 2.366 | 3.57E-05 | -4.18 | -6.56 | 0.48 | 0.26 |  |
| *Gm13842* | 2.366 | 9.15E-05 | -4.18 | -6.56 | 0.48 | 0.26 |  |
| *Gm11646* | 2.366 | 1.01E-04 | -4.18 | -6.56 | 0.48 | 0.26 |  |
| *Gm15992* | 2.366 | 1.63E-04 | -4.18 | -6.56 | 0.48 | 0.26 |  |
| *Scarna3b* | 2.366 | 1.67E-04 | -4.18 | -6.56 | 0.48 | 0.26 |  |
| *Gm13000* | 2.366 | 1.72E-06 | -4.18 | -6.56 | 0.48 | 0.26 |  |
| *Gm12321* | 2.366 | 2.53E-06 | -4.18 | -6.56 | 0.48 | 0.26 |  |
| *Muc5b* | 2.366 | 1.80E-05 | -4.18 | -6.56 | 0.48 | 0.26 |  |
| *Gm13809* | 2.366 | 2.10E-05 | -4.18 | -6.56 | 0.48 | 0.26 |  |
| *Cfap52* | 2.366 | 3.39E-05 | -4.18 | -6.56 | 0.48 | 0.26 |  |
| *Gm11760* | 2.358 | 1.19E-04 | -3.79 | -6.16 | 0.52 | 0.50 |  |
| *Hist1h2bb* | 2.358 | 3.38E-04 | -3.79 | -6.16 | 0.52 | 0.50 |  |
| *Gm10284* | 2.349 | 2.91E-03 | -3.08 | -5.46 | 0.42 | 0.73 |  |
| *Pcdhb6* | 2.341 | 1.17E-03 | -3.79 | -5.98 | 0.52 | 0.42 |  |
| *Gm18284* | 2.336 | 4.10E-03 | -3.21 | -5.46 | 0.17 | 0.53 |  |
| *Rpl21-ps12* | 2.310 | 4.80E-03 | -3.21 | -5.77 | 0.17 | 0.69 |  |
| *Gm5871* | 2.299 | 5.61E-05 | -3.79 | -6.16 | 0.31 | 0.43 |  |
| *Cdh19* | 2.294 | 1.43E-02 | -2.74 | -5.19 | 0.59 | 0.82 |  |
| *Trbv13-1* | 2.292 | 4.62E-03 | -3.71 | -5.98 | 0.77 | 0.42 |  |
| *1700034E13Rik* | 2.288 | 2.99E-06 | -4.39 | -6.56 | 0.71 | 0.26 |  |
| *4930570D08Rik* | 2.288 | 2.99E-06 | -4.39 | -6.56 | 0.71 | 0.26 |  |
| *1700018B08Rik* | 2.288 | 7.61E-06 | -4.39 | -6.56 | 0.71 | 0.26 |  |
| *Fscn3* | 2.288 | 3.71E-05 | -4.39 | -6.56 | 0.71 | 0.26 |  |
| *Ighv1-18* | 2.288 | 9.48E-05 | -4.39 | -6.56 | 0.71 | 0.26 |  |
| *1700013G24Rik* | 2.288 | 1.11E-04 | -4.39 | -6.56 | 0.71 | 0.26 |  |
| *Sftpc* | 2.288 | 1.28E-04 | -4.39 | -6.56 | 0.71 | 0.26 |  |
| *Malrd1* | 2.288 | 2.10E-04 | -4.39 | -6.56 | 0.71 | 0.26 |  |
| *Hrc* | 2.285 | 5.98E-03 | -3.02 | -5.58 | 0.95 | 0.78 | CVD |
| *Gm9144* | 2.279 | 1.12E-02 | -3.23 | -5.40 | 0.70 | 0.57 |  |
| *Frmpd4* | 2.277 | 5.45E-05 | -3.81 | -5.98 | 0.71 | 0.42 | Diabetes, CVD |
| *Gm13620* | 2.270 | 1.86E-03 | -4.00 | -6.16 | 0.65 | 0.26 |  |
| *1700048M11Rik* | 2.265 | 3.16E-03 | -3.79 | -6.16 | 0.73 | 0.50 |  |
| *Txlnb* | 2.258 | 2.19E-03 | -2.16 | -4.30 | 0.36 | 0.43 |  |
| *Tnni3k* | 2.253 | 1.89E-07 | -4.18 | -6.56 | 0.28 | 0.26 | Obesity, Diabetes, CVD |
| *Foxd3* | 2.253 | 1.89E-07 | -4.18 | -6.56 | 0.28 | 0.26 | Diabetes, CVD |
| *Gm15812* | 2.253 | 1.89E-07 | -4.18 | -6.56 | 0.28 | 0.26 |  |
| *Nppa* | 2.253 | 2.61E-07 | -4.18 | -6.56 | 0.28 | 0.26 | Obesity, Diabetes, CVD |
| *Gm12400* | 2.253 | 2.34E-06 | -4.18 | -6.56 | 0.28 | 0.26 |  |
| *Gm22980* | 2.253 | 2.44E-06 | -4.18 | -6.56 | 0.28 | 0.26 |  |
| *Olfr520* | 2.253 | 5.02E-06 | -4.18 | -6.56 | 0.28 | 0.26 |  |
| *C230012O17Rik* | 2.253 | 1.35E-05 | -4.18 | -6.56 | 0.28 | 0.26 |  |
| *Gm25732* | 2.253 | 1.55E-05 | -4.18 | -6.56 | 0.28 | 0.26 |  |
| *Hist1h2ad* | 2.253 | 1.86E-05 | -4.18 | -6.56 | 0.28 | 0.26 |  |
| *Gm14051* | 2.253 | 2.13E-05 | -4.18 | -6.56 | 0.28 | 0.26 |  |
| *Gm13937* | 2.253 | 3.58E-05 | -4.18 | -6.56 | 0.28 | 0.26 |  |
| *Psd2* | 2.253 | 3.83E-05 | -4.18 | -6.56 | 0.28 | 0.26 |  |
| *Gm11639* | 2.253 | 4.02E-05 | -4.18 | -6.56 | 0.28 | 0.26 |  |
| *Gm12098* | 2.253 | 4.83E-05 | -4.18 | -6.56 | 0.28 | 0.26 |  |
| *Scube2* | 2.253 | 7.91E-05 | -4.18 | -6.56 | 0.28 | 0.26 | CVD |
| *Gm26517* | 2.253 | 2.12E-04 | -4.18 | -6.56 | 0.28 | 0.26 |  |
| *Gm22455* | 2.253 | 2.60E-04 | -4.18 | -6.56 | 0.28 | 0.26 |  |
| *Ccdc85a* | 2.253 | 3.38E-04 | -4.18 | -6.56 | 0.28 | 0.26 | Obesity |
| *Mir1960* | 2.253 | 3.48E-04 | -4.18 | -6.56 | 0.28 | 0.26 |  |
| *Gm13816* | 2.253 | 3.51E-04 | -4.18 | -6.56 | 0.28 | 0.26 |  |
| *H2-Ke6* | 2.250 | 3.77E-03 | -3.88 | -5.86 | 0.84 | 0.54 |  |
| *Enthd1* | 2.250 | 2.80E-06 | -4.39 | -6.56 | 0.69 | 0.26 |  |
| *1700042G07Rik* | 2.250 | 2.80E-06 | -4.39 | -6.56 | 0.69 | 0.26 |  |
| *C1ql2* | 2.250 | 9.63E-06 | -4.39 | -6.56 | 0.69 | 0.26 |  |
| *Rps19-ps9* | 2.250 | 3.80E-05 | -4.39 | -6.56 | 0.69 | 0.26 |  |
| *Gm22311* | 2.250 | 3.98E-05 | -4.39 | -6.56 | 0.69 | 0.26 |  |
| *Gm9378* | 2.250 | 1.30E-04 | -4.39 | -6.56 | 0.69 | 0.26 |  |
| *Myl3* | 2.249 | 1.51E-02 | -2.45 | -4.48 | 1.29 | 0.23 | Diabetes, CVD |
| *Gm3086* | 2.247 | 3.99E-04 | -3.81 | -5.98 | 0.68 | 0.42 |  |
| *Gm12356* | 2.238 | 3.80E-04 | -4.00 | -6.16 | 0.63 | 0.26 |  |
| *Cd200r3* | 2.238 | 1.22E-03 | -4.00 | -6.16 | 0.63 | 0.26 |  |
| *Lrrc2* | 2.234 | 4.33E-03 | -3.39 | -5.77 | 0.55 | 0.86 |  |
| *Camp* | 2.233 | 2.26E-02 | -3.48 | -5.58 | 0.62 | 0.38 |  |
| *Acod1* | -2.232 | 1.07E-02 | -4.27 | -2.25 | 0.62 | 0.59 |  |
| *Gm16365* | 2.213 | 2.90E-03 | -3.39 | -5.46 | 0.18 | 0.47 |  |
| *En2* | 2.212 | 1.25E-04 | -4.00 | -6.16 | 0.59 | 0.26 |  |
| *Ms4a4c* | -2.211 | 8.98E-04 | -3.60 | -1.63 | 0.64 | 0.37 |  |
| *Serpina3a* | 2.211 | 6.98E-03 | -2.59 | -4.88 | 0.29 | 0.65 |  |
| *E530011L22Rik* | 2.210 | 5.26E-03 | -3.60 | -5.86 | 0.56 | 0.77 |  |
| *Gm3809* | 2.197 | 7.25E-03 | -3.21 | -5.28 | 0.17 | 0.54 |  |
| *Gm13612* | 2.191 | 3.13E-03 | -3.21 | -5.37 | 0.31 | 0.76 |  |
| *Igf2os* | 2.189 | 9.25E-04 | -4.00 | -6.16 | 0.57 | 0.26 |  |
| *Fgf13* | 2.177 | 1.99E-03 | -3.60 | -5.58 | 0.63 | 0.38 | Diabetes, CVD |
| *Gm13511* | 2.175 | 9.99E-05 | -4.27 | -6.56 | 0.62 | 0.26 |  |
| *Gm13009* | 2.175 | 1.44E-04 | -4.27 | -6.56 | 0.62 | 0.26 |  |
| *Gm14425* | 2.175 | 8.54E-04 | -4.27 | -6.56 | 0.62 | 0.26 |  |
| *Fmr1nb* | 2.175 | 1.40E-03 | -4.27 | -6.56 | 0.62 | 0.26 |  |
| *Bmp8b* | 2.174 | 7.19E-03 | -3.48 | -5.58 | 0.57 | 0.44 | Obesity |
| *Gm23130* | 2.164 | 1.50E-03 | -4.00 | -6.16 | 0.63 | 0.43 |  |
| *Wdr72* | 2.164 | 4.44E-03 | -3.60 | -5.77 | 0.46 | 0.38 | Diabetes |
| *Syngr4* | 2.156 | 1.65E-03 | -3.08 | -5.19 | 0.25 | 0.34 |  |
| *Gm8508* | 2.152 | 3.32E-03 | -2.55 | -4.72 | 0.53 | 0.47 |  |
| *Gm13181* | 2.150 | 7.90E-03 | -3.08 | -5.19 | 0.58 | 0.25 |  |
| *A530020G20Rik* | -2.148 | 8.59E-03 | -4.27 | -2.13 | 0.62 | 0.36 |  |
| *Ttn* | 2.147 | 2.85E-02 | -1.55 | -3.39 | 1.28 | 0.44 | Diabetes, CVD |
| *Gm14017* | 2.142 | 4.39E-04 | -4.00 | -6.16 | 0.65 | 0.50 |  |
| *Gm7221* | -2.141 | 4.43E-05 | -4.39 | -2.28 | 0.50 | 0.20 |  |
| *Gm11951* | 2.138 | 5.50E-04 | -4.00 | -6.16 | 0.59 | 0.43 |  |
| *Rps2-ps13* | 2.138 | 6.50E-04 | -4.00 | -6.16 | 0.59 | 0.43 |  |
| *Shcbp1* | -2.137 | 1.17E-03 | -3.32 | -1.20 | 0.59 | 0.26 |  |
| *Mirlet7c-2* | 2.135 | 1.93E-03 | -4.00 | -5.86 | 0.76 | 0.54 |  |
| *Gm8444* | 2.125 | 8.81E-04 | -3.81 | -6.16 | 0.49 | 0.50 |  |
| *Gm12525* | 2.125 | 8.23E-05 | -4.00 | -5.98 | 0.65 | 0.42 |  |
| *Akap3* | 2.122 | 7.43E-03 | -3.60 | -5.46 | 0.98 | 0.53 |  |
| *Olfr726* | 2.119 | 1.65E-03 | -3.79 | -5.77 | 0.52 | 0.29 |  |
| *Fabp5l2* | 2.117 | 9.43E-04 | -3.57 | -5.77 | 0.63 | 0.29 |  |
| *Gm15353* | 2.116 | 4.32E-03 | -4.00 | -6.16 | 0.57 | 0.43 |  |
| *Tmem40* | 2.116 | 9.02E-03 | -2.90 | -5.19 | 0.32 | 0.65 |  |
| *Gm15798* | 2.110 | 2.32E-03 | -4.00 | -6.16 | 0.63 | 0.50 |  |
| *Gm17276* | 2.098 | 8.77E-03 | -2.34 | -4.39 | 0.41 | 0.60 |  |
| *Adam24* | 2.098 | 5.06E-04 | -4.18 | -6.16 | 0.64 | 0.43 |  |
| *Gm9009* | 2.098 | 5.33E-04 | -4.18 | -6.16 | 0.64 | 0.43 |  |
| *Pcdha11* | 2.098 | 8.48E-04 | -4.18 | -6.16 | 0.64 | 0.43 |  |
| *Gm8392* | 2.098 | 9.47E-04 | -4.18 | -6.16 | 0.64 | 0.43 |  |
| *Iqgap3* | -2.092 | 8.80E-03 | -3.71 | -1.68 | 0.77 | 0.29 |  |
| *Gm6793* | 2.091 | 1.01E-02 | -3.60 | -5.58 | 0.63 | 0.53 |  |
| *Traf3ip3* | -2.087 | 3.77E-04 | -4.27 | -2.28 | 0.62 | 0.18 |  |
| *Smpx* | 2.086 | 3.43E-05 | -4.00 | -6.16 | 0.42 | 0.26 |  |
| *Gm11620* | 2.086 | 3.13E-03 | -4.00 | -6.16 | 0.42 | 0.26 |  |
| *Myom2* | 2.086 | 3.72E-04 | -4.00 | -6.16 | 0.42 | 0.26 |  |
| *Cytl1* | 2.084 | 2.20E-02 | -2.54 | -4.70 | 0.78 | 1.00 |  |
| *Dnah5* | -2.083 | 1.42E-03 | -4.27 | -2.34 | 0.62 | 0.47 | CVD |
| *Slc6a19* | 2.083 | 3.00E-03 | -4.00 | -6.16 | 0.59 | 0.50 | CVD |
| *Gm24407* | 2.077 | 3.27E-03 | -3.88 | -5.77 | 0.84 | 0.38 |  |
| *Gm8773* | 2.076 | 2.01E-06 | -4.58 | -6.56 | 0.53 | 0.26 |  |
| *Emx1* | 2.076 | 2.33E-06 | -4.58 | -6.56 | 0.53 | 0.26 |  |
| *Gm16351* | 2.076 | 2.33E-06 | -4.58 | -6.56 | 0.53 | 0.26 |  |
| *Prss21* | 2.076 | 2.35E-06 | -4.58 | -6.56 | 0.53 | 0.26 |  |
| *Padi1* | 2.076 | 2.35E-06 | -4.58 | -6.56 | 0.53 | 0.26 |  |
| *Piwil1* | 2.076 | 2.35E-06 | -4.58 | -6.56 | 0.53 | 0.26 |  |
| *Tgif2-ps2* | 2.076 | 2.35E-06 | -4.58 | -6.56 | 0.53 | 0.26 |  |
| *Spag16* | 2.076 | 2.35E-06 | -4.58 | -6.56 | 0.53 | 0.26 | Obesity, Diabetes |
| *Zdhhc25* | 2.076 | 2.35E-06 | -4.58 | -6.56 | 0.53 | 0.26 |  |
| *Ap3b2* | 2.076 | 2.35E-06 | -4.58 | -6.56 | 0.53 | 0.26 |  |
| *Rnu12* | 2.076 | 2.35E-06 | -4.58 | -6.56 | 0.53 | 0.26 |  |
| *Gm15067* | 2.076 | 2.35E-06 | -4.58 | -6.56 | 0.53 | 0.26 |  |
| *Gm13126* | 2.076 | 2.35E-06 | -4.58 | -6.56 | 0.53 | 0.26 |  |
| *Gm13050* | 2.076 | 2.35E-06 | -4.58 | -6.56 | 0.53 | 0.26 |  |
| *Rps19-ps14* | 2.076 | 2.35E-06 | -4.58 | -6.56 | 0.53 | 0.26 |  |
| *Gm5942* | 2.076 | 2.35E-06 | -4.58 | -6.56 | 0.53 | 0.26 |  |
| *Gm4991* | 2.076 | 2.35E-06 | -4.58 | -6.56 | 0.53 | 0.26 |  |
| *Gm13715* | 2.076 | 2.35E-06 | -4.58 | -6.56 | 0.53 | 0.26 |  |
| *Gm15873* | 2.076 | 2.35E-06 | -4.58 | -6.56 | 0.53 | 0.26 |  |
| *4933406K04Rik* | 2.076 | 2.35E-06 | -4.58 | -6.56 | 0.53 | 0.26 |  |
| *Ccdc42os* | 2.076 | 2.35E-06 | -4.58 | -6.56 | 0.53 | 0.26 |  |
| *Gm22518* | 2.076 | 2.35E-06 | -4.58 | -6.56 | 0.53 | 0.26 |  |
| *Cdk19os* | 2.076 | 3.67E-06 | -4.58 | -6.56 | 0.53 | 0.26 |  |
| *Gm16229* | 2.076 | 4.96E-06 | -4.58 | -6.56 | 0.53 | 0.26 |  |
| *Gm22092* | 2.076 | 6.44E-06 | -4.58 | -6.56 | 0.53 | 0.26 |  |
| *Gm8419* | 2.076 | 6.91E-06 | -4.58 | -6.56 | 0.53 | 0.26 |  |
| *Gm13915* | 2.076 | 7.74E-06 | -4.58 | -6.56 | 0.53 | 0.26 |  |
| *Gm26004* | 2.076 | 8.54E-06 | -4.58 | -6.56 | 0.53 | 0.26 |  |
| *Mir7659* | 2.076 | 8.87E-06 | -4.58 | -6.56 | 0.53 | 0.26 |  |
| *Gm17752* | 2.076 | 9.43E-06 | -4.58 | -6.56 | 0.53 | 0.26 |  |
| *Gdap1l1* | 2.076 | 9.71E-06 | -4.58 | -6.56 | 0.53 | 0.26 |  |
| *Gm17189* | 2.076 | 9.76E-06 | -4.58 | -6.56 | 0.53 | 0.26 |  |
| *2900072N19Rik* | 2.076 | 1.01E-05 | -4.58 | -6.56 | 0.53 | 0.26 |  |
| *Gm16578* | 2.076 | 1.01E-05 | -4.58 | -6.56 | 0.53 | 0.26 |  |
| *Cfap44* | 2.076 | 1.02E-05 | -4.58 | -6.56 | 0.53 | 0.26 |  |
| *Gm3943* | 2.076 | 1.07E-05 | -4.58 | -6.56 | 0.53 | 0.26 |  |
| *Slfn5os* | 2.076 | 1.27E-05 | -4.58 | -6.56 | 0.53 | 0.26 |  |
| *Gm22613* | 2.076 | 1.30E-05 | -4.58 | -6.56 | 0.53 | 0.26 |  |
| *1700030M09Rik* | 2.076 | 1.47E-05 | -4.58 | -6.56 | 0.53 | 0.26 |  |
| *Gm12269* | 2.076 | 1.51E-05 | -4.58 | -6.56 | 0.53 | 0.26 |  |
| *Slc5a5* | 2.076 | 1.60E-05 | -4.58 | -6.56 | 0.53 | 0.26 |  |
| *Gm3617* | 2.076 | 1.68E-05 | -4.58 | -6.56 | 0.53 | 0.26 |  |
| *Gm11479* | 2.076 | 1.80E-05 | -4.58 | -6.56 | 0.53 | 0.26 |  |
| *Gm15432* | 2.076 | 1.80E-05 | -4.58 | -6.56 | 0.53 | 0.26 |  |
| *Gm10689* | 2.076 | 2.17E-05 | -4.58 | -6.56 | 0.53 | 0.26 |  |
| *Gm26465* | 2.076 | 2.71E-05 | -4.58 | -6.56 | 0.53 | 0.26 |  |
| *Gm24693* | 2.076 | 3.04E-05 | -4.58 | -6.56 | 0.53 | 0.26 |  |
| *Gm13703* | 2.076 | 3.08E-05 | -4.58 | -6.56 | 0.53 | 0.26 |  |
| *Il5ra* | 2.076 | 4.17E-05 | -4.58 | -6.56 | 0.53 | 0.26 | Obesity, Diabetes, CVD |
| *Ighv1-59* | 2.076 | 4.31E-05 | -4.58 | -6.56 | 0.53 | 0.26 |  |
| *Zfp92* | 2.076 | 4.87E-05 | -4.58 | -6.56 | 0.53 | 0.26 |  |
| *Gm27514* | 2.076 | 4.98E-05 | -4.58 | -6.56 | 0.53 | 0.26 |  |
| *Clec2l* | 2.076 | 4.99E-05 | -4.58 | -6.56 | 0.53 | 0.26 |  |
| *Gm6181* | 2.076 | 5.21E-05 | -4.58 | -6.56 | 0.53 | 0.26 |  |
| *Gm5321* | 2.076 | 5.80E-05 | -4.58 | -6.56 | 0.53 | 0.26 |  |
| *Gm14094* | 2.076 | 6.74E-05 | -4.58 | -6.56 | 0.53 | 0.26 |  |
| *Ighv1-80* | 2.076 | 6.83E-05 | -4.58 | -6.56 | 0.53 | 0.26 |  |
| *Aire* | 2.076 | 8.50E-05 | -4.58 | -6.56 | 0.53 | 0.26 | Obesity, Diabetes |
| *Gm17199* | 2.076 | 1.49E-04 | -4.58 | -6.56 | 0.53 | 0.26 |  |
| *Sox5os5* | 2.076 | 1.61E-04 | -4.58 | -6.56 | 0.53 | 0.26 |  |
| *Hmgb1-ps6* | 2.076 | 1.92E-04 | -4.58 | -6.56 | 0.53 | 0.26 |  |
| *Gm12407* | 2.076 | 2.01E-04 | -4.58 | -6.56 | 0.53 | 0.26 |  |
| *Ces2f* | 2.076 | 2.06E-04 | -4.58 | -6.56 | 0.53 | 0.26 |  |
| *Vwa5b2* | 2.076 | 2.65E-04 | -4.58 | -6.56 | 0.53 | 0.26 |  |
| *Tspan1* | 2.076 | 3.00E-04 | -4.58 | -6.56 | 0.53 | 0.26 | Diabetes |
| *Mir6921* | 2.076 | 3.59E-04 | -4.58 | -6.56 | 0.53 | 0.26 |  |
| *Gm4943* | 2.076 | 5.01E-04 | -4.58 | -6.56 | 0.53 | 0.26 |  |
| *Stac* | 2.076 | 4.78E-03 | -4.58 | -6.56 | 0.53 | 0.26 |  |
| *Gm23851* | 2.074 | 1.67E-03 | -3.79 | -5.77 | 0.52 | 0.38 |  |
| *Ccdc13* | 2.071 | 1.68E-06 | -4.39 | -6.56 | 0.50 | 0.26 |  |
| *Gm1335* | 2.071 | 1.72E-06 | -4.39 | -6.56 | 0.50 | 0.26 |  |
| *Gm23640* | 2.071 | 1.72E-06 | -4.39 | -6.56 | 0.50 | 0.26 |  |
| *Gm12998* | 2.071 | 4.25E-06 | -4.39 | -6.56 | 0.50 | 0.26 |  |
| *Gal3st3* | 2.071 | 1.64E-05 | -4.39 | -6.56 | 0.50 | 0.26 |  |
| *Gm14226* | 2.071 | 1.64E-05 | -4.39 | -6.56 | 0.50 | 0.26 |  |
| *Gm29200* | 2.071 | 2.13E-05 | -4.39 | -6.56 | 0.50 | 0.26 |  |
| *Gm2976* | 2.071 | 3.45E-05 | -4.39 | -6.56 | 0.50 | 0.26 |  |
| *Gm15763* | 2.071 | 4.53E-05 | -4.39 | -6.56 | 0.50 | 0.26 |  |
| *Tecrl* | 2.071 | 5.21E-05 | -4.39 | -6.56 | 0.50 | 0.26 | Diabetes, CVD |
| *Gm13523* | 2.071 | 6.44E-05 | -4.39 | -6.56 | 0.50 | 0.26 |  |
| *Gm26685* | 2.071 | 7.37E-05 | -4.39 | -6.56 | 0.50 | 0.26 |  |
| *Gm15483* | 2.071 | 1.95E-04 | -4.39 | -6.56 | 0.50 | 0.26 |  |
| *Fscn2* | 2.071 | 2.44E-04 | -4.39 | -6.56 | 0.50 | 0.26 |  |
| *Gm24714* | 2.071 | 2.58E-04 | -4.39 | -6.56 | 0.50 | 0.26 |  |
| *Gm26706* | 2.071 | 4.01E-04 | -4.39 | -6.56 | 0.50 | 0.26 |  |
| *Gm16725* | 2.071 | 4.32E-04 | -4.39 | -6.56 | 0.50 | 0.26 |  |
| *1700061E17Rik* | 2.071 | 4.45E-04 | -4.39 | -6.56 | 0.50 | 0.26 |  |
| *Gm15965* | 2.071 | 7.07E-04 | -4.39 | -6.56 | 0.50 | 0.26 |  |
| *Gm26477* | 2.071 | 1.72E-06 | -4.39 | -6.56 | 0.50 | 0.26 |  |
| *Gm8597* | 2.071 | 1.72E-06 | -4.39 | -6.56 | 0.50 | 0.26 |  |
| *Plppr4* | 2.071 | 9.26E-06 | -4.39 | -6.56 | 0.50 | 0.26 |  |
| *Gja3* | 2.071 | 1.14E-05 | -4.39 | -6.56 | 0.50 | 0.26 |  |
| *Gm25057* | 2.071 | 1.31E-05 | -4.39 | -6.56 | 0.50 | 0.26 |  |
| *Gm24146* | 2.071 | 1.86E-05 | -4.39 | -6.56 | 0.50 | 0.26 |  |
| *Mir6392* | 2.071 | 2.35E-04 | -4.39 | -6.56 | 0.50 | 0.26 |  |
| *Eya1* | 2.071 | 2.54E-04 | -4.39 | -6.56 | 0.50 | 0.26 |  |
| *Igkv5-45* | 2.071 | 2.80E-04 | -4.39 | -6.56 | 0.50 | 0.26 | CVD |
| *Btnl10* | 2.071 | 4.78E-04 | -4.39 | -6.56 | 0.50 | 0.26 |  |
| *Igkv3-7* | 2.067 | 3.61E-03 | -3.88 | -6.16 | 0.52 | 0.50 |  |
| *Gm24105* | 2.066 | 8.59E-03 | -3.39 | -5.40 | 0.65 | 0.57 |  |
| *C4bp-ps1* | 2.065 | 2.40E-02 | -2.62 | -4.79 | 0.47 | 0.70 |  |
| *Arxes2* | 2.062 | 5.74E-03 | -4.00 | -6.16 | 0.65 | 0.63 |  |
| *Gm12470* | 2.059 | 1.85E-05 | -4.18 | -6.16 | 0.50 | 0.26 |  |
| *Ccdc113* | 2.059 | 5.62E-05 | -4.18 | -6.16 | 0.50 | 0.26 |  |
| *Gm11682* | 2.059 | 1.40E-04 | -4.18 | -6.16 | 0.50 | 0.26 |  |
| *Gm6564* | 2.059 | 1.46E-04 | -4.18 | -6.16 | 0.50 | 0.26 |  |
| *Gm17798* | 2.059 | 5.81E-04 | -4.18 | -6.16 | 0.50 | 0.26 |  |
| *Gm5445* | 2.059 | 2.22E-03 | -4.18 | -6.16 | 0.50 | 0.26 |  |
| *Gm5575* | 2.058 | 1.64E-04 | -4.18 | -6.16 | 0.50 | 0.26 |  |
| *Mypn* | 2.058 | 1.53E-02 | -2.90 | -4.88 | 0.39 | 0.36 |  |
| *Atg4a-ps* | 2.058 | 1.81E-03 | -3.79 | -5.98 | 0.31 | 0.66 |  |
| *Gm2735* | 2.048 | 1.57E-06 | -4.58 | -6.56 | 0.51 | 0.26 |  |
| *Gm25881* | 2.048 | 1.58E-06 | -4.58 | -6.56 | 0.51 | 0.26 |  |
| *Gm15714* | 2.048 | 2.16E-06 | -4.58 | -6.56 | 0.51 | 0.26 |  |
| *Dmrtc2* | 2.048 | 2.19E-06 | -4.58 | -6.56 | 0.51 | 0.26 |  |
| *Tmbim7* | 2.048 | 2.19E-06 | -4.58 | -6.56 | 0.51 | 0.26 |  |
| *Vsx2* | 2.048 | 2.19E-06 | -4.58 | -6.56 | 0.51 | 0.26 |  |
| *Odf3l1* | 2.048 | 2.19E-06 | -4.58 | -6.56 | 0.51 | 0.26 |  |
| *Cypt3* | 2.048 | 2.19E-06 | -4.58 | -6.56 | 0.51 | 0.26 |  |
| *Hdhd1a* | 2.048 | 2.19E-06 | -4.58 | -6.56 | 0.51 | 0.26 |  |
| *4930469G21Rik* | 2.048 | 2.19E-06 | -4.58 | -6.56 | 0.51 | 0.26 |  |
| *Grm5* | 2.048 | 2.19E-06 | -4.58 | -6.56 | 0.51 | 0.26 | Diabetes, CVD |
| *Mrgprg* | 2.048 | 2.19E-06 | -4.58 | -6.56 | 0.51 | 0.26 |  |
| *Olfr1029* | 2.048 | 2.19E-06 | -4.58 | -6.56 | 0.51 | 0.26 |  |
| *Nkx6-3* | 2.048 | 2.19E-06 | -4.58 | -6.56 | 0.51 | 0.26 |  |
| *Gm22772* | 2.048 | 2.19E-06 | -4.58 | -6.56 | 0.51 | 0.26 |  |
| *Gm15176* | 2.048 | 2.19E-06 | -4.58 | -6.56 | 0.51 | 0.26 |  |
| *Gm5395* | 2.048 | 2.19E-06 | -4.58 | -6.56 | 0.51 | 0.26 |  |
| *Gm15580* | 2.048 | 2.19E-06 | -4.58 | -6.56 | 0.51 | 0.26 |  |
| *H2af-ps* | 2.048 | 2.19E-06 | -4.58 | -6.56 | 0.51 | 0.26 |  |
| *Gm15168* | 2.048 | 2.19E-06 | -4.58 | -6.56 | 0.51 | 0.26 |  |
| *Gm16330* | 2.048 | 2.19E-06 | -4.58 | -6.56 | 0.51 | 0.26 |  |
| *4930557F10Rik* | 2.048 | 2.19E-06 | -4.58 | -6.56 | 0.51 | 0.26 |  |
| *Gm15721* | 2.048 | 2.19E-06 | -4.58 | -6.56 | 0.51 | 0.26 |  |
| *Gm15668* | 2.048 | 2.19E-06 | -4.58 | -6.56 | 0.51 | 0.26 |  |
| *4930401O12Rik* | 2.048 | 2.19E-06 | -4.58 | -6.56 | 0.51 | 0.26 |  |
| *Gm9719* | 2.048 | 2.19E-06 | -4.58 | -6.56 | 0.51 | 0.26 |  |
| *Gm15849* | 2.048 | 2.19E-06 | -4.58 | -6.56 | 0.51 | 0.26 |  |
| *Tmem207* | 2.048 | 2.19E-06 | -4.58 | -6.56 | 0.51 | 0.26 |  |
| *Vmn2r-ps19* | 2.048 | 2.19E-06 | -4.58 | -6.56 | 0.51 | 0.26 |  |
| *Gm26783* | 2.048 | 2.19E-06 | -4.58 | -6.56 | 0.51 | 0.26 |  |
| *1700034K08Rik* | 2.048 | 2.19E-06 | -4.58 | -6.56 | 0.51 | 0.26 |  |
| *1700025F24Rik* | 2.048 | 2.19E-06 | -4.58 | -6.56 | 0.51 | 0.26 |  |
| *Gm29395* | 2.048 | 2.19E-06 | -4.58 | -6.56 | 0.51 | 0.26 |  |
| *Rps13-ps5* | 2.048 | 2.48E-06 | -4.58 | -6.56 | 0.51 | 0.26 |  |
| *Gm11774* | 2.048 | 2.74E-06 | -4.58 | -6.56 | 0.51 | 0.26 |  |
| *Gm13921* | 2.048 | 3.23E-06 | -4.58 | -6.56 | 0.51 | 0.26 |  |
| *Gm8659* | 2.048 | 3.95E-06 | -4.58 | -6.56 | 0.51 | 0.26 |  |
| *Gm13414* | 2.048 | 3.95E-06 | -4.58 | -6.56 | 0.51 | 0.26 |  |
| *Vmn1r79* | 2.048 | 3.95E-06 | -4.58 | -6.56 | 0.51 | 0.26 |  |
| *Gm14106* | 2.048 | 4.25E-06 | -4.58 | -6.56 | 0.51 | 0.26 |  |
| *Gm13294* | 2.048 | 6.22E-06 | -4.58 | -6.56 | 0.51 | 0.26 |  |
| *Mir6939* | 2.048 | 6.68E-06 | -4.58 | -6.56 | 0.51 | 0.26 |  |
| *Haglr* | 2.048 | 6.80E-06 | -4.58 | -6.56 | 0.51 | 0.26 |  |
| *Gssos1* | 2.048 | 6.95E-06 | -4.58 | -6.56 | 0.51 | 0.26 |  |
| *Gm10087* | 2.048 | 8.28E-06 | -4.58 | -6.56 | 0.51 | 0.26 |  |
| *Hmgb1-rs18* | 2.048 | 9.94E-06 | -4.58 | -6.56 | 0.51 | 0.26 |  |
| *Gm5105* | 2.048 | 1.05E-05 | -4.58 | -6.56 | 0.51 | 0.26 |  |
| *Gm26928* | 2.048 | 1.10E-05 | -4.58 | -6.56 | 0.51 | 0.26 |  |
| *Rsph14* | 2.048 | 1.12E-05 | -4.58 | -6.56 | 0.51 | 0.26 |  |
| *Fbxo39* | 2.048 | 1.12E-05 | -4.58 | -6.56 | 0.51 | 0.26 |  |
| *Slc16a14* | 2.048 | 1.21E-05 | -4.58 | -6.56 | 0.51 | 0.26 |  |
| *Slco6c1* | 2.048 | 1.28E-05 | -4.58 | -6.56 | 0.51 | 0.26 |  |
| *Gm25687* | 2.048 | 1.28E-05 | -4.58 | -6.56 | 0.51 | 0.26 |  |
| *A430108G06Rik* | 2.048 | 1.35E-05 | -4.58 | -6.56 | 0.51 | 0.26 |  |
| *Gm136* | 2.048 | 1.41E-05 | -4.58 | -6.56 | 0.51 | 0.26 |  |
| *Gm26614* | 2.048 | 1.47E-05 | -4.58 | -6.56 | 0.51 | 0.26 |  |
| *Gm14769* | 2.048 | 1.51E-05 | -4.58 | -6.56 | 0.51 | 0.26 |  |
| *Synpr* | 2.048 | 1.59E-05 | -4.58 | -6.56 | 0.51 | 0.26 |  |
| *Slc36a3os* | 2.048 | 1.66E-05 | -4.58 | -6.56 | 0.51 | 0.26 |  |
| *Gm5451* | 2.048 | 1.67E-05 | -4.58 | -6.56 | 0.51 | 0.26 |  |
| *Gm10800* | 2.048 | 1.72E-05 | -4.58 | -6.56 | 0.51 | 0.26 |  |
| *Apobec4* | 2.048 | 2.23E-05 | -4.58 | -6.56 | 0.51 | 0.26 |  |
| *Gm14170* | 2.048 | 2.32E-05 | -4.58 | -6.56 | 0.51 | 0.26 |  |
| *Gm8129* | 2.048 | 3.12E-05 | -4.58 | -6.56 | 0.51 | 0.26 |  |
| *Gm13584* | 2.048 | 3.13E-05 | -4.58 | -6.56 | 0.51 | 0.26 |  |
| *Tnnt3* | 2.048 | 3.31E-05 | -4.58 | -6.56 | 0.51 | 0.26 | Diabetes, CVD |
| *Snord111* | 2.048 | 3.83E-05 | -4.58 | -6.56 | 0.51 | 0.26 |  |
| *BC016579* | 2.048 | 4.92E-05 | -4.58 | -6.56 | 0.51 | 0.26 |  |
| *Gm8338* | 2.048 | 4.97E-05 | -4.58 | -6.56 | 0.51 | 0.26 |  |
| *Gm27193* | 2.048 | 6.33E-05 | -4.58 | -6.56 | 0.51 | 0.26 |  |
| *Gm16265* | 2.048 | 7.46E-05 | -4.58 | -6.56 | 0.51 | 0.26 |  |
| *Gm12808* | 2.048 | 7.67E-05 | -4.58 | -6.56 | 0.51 | 0.26 |  |
| *Gm5396* | 2.048 | 7.97E-05 | -4.58 | -6.56 | 0.51 | 0.26 |  |
| *Olfr755-ps1* | 2.048 | 9.73E-05 | -4.58 | -6.56 | 0.51 | 0.26 |  |
| *Gm25235* | 2.048 | 9.84E-05 | -4.58 | -6.56 | 0.51 | 0.26 |  |
| *Gm22067* | 2.048 | 1.01E-04 | -4.58 | -6.56 | 0.51 | 0.26 |  |
| *Gm11438* | 2.048 | 1.12E-04 | -4.58 | -6.56 | 0.51 | 0.26 |  |
| *Ctsj* | 2.048 | 1.25E-04 | -4.58 | -6.56 | 0.51 | 0.26 |  |
| *Gm17828* | 2.048 | 1.60E-04 | -4.58 | -6.56 | 0.51 | 0.26 |  |
| *Gm28586* | 2.048 | 1.65E-04 | -4.58 | -6.56 | 0.51 | 0.26 |  |
| *Dhx58os* | 2.048 | 1.88E-04 | -4.58 | -6.56 | 0.51 | 0.26 |  |
| *Zfp385c* | 2.048 | 1.92E-04 | -4.58 | -6.56 | 0.51 | 0.26 |  |
| *Ptchd4* | 2.048 | 2.06E-04 | -4.58 | -6.56 | 0.51 | 0.26 |  |
| *Gm12260* | 2.048 | 2.19E-04 | -4.58 | -6.56 | 0.51 | 0.26 |  |
| *Gm5759* | 2.048 | 2.46E-04 | -4.58 | -6.56 | 0.51 | 0.26 |  |
| *Gm2670* | 2.048 | 2.94E-04 | -4.58 | -6.56 | 0.51 | 0.26 |  |
| *Tarm1* | 2.048 | 3.70E-04 | -4.58 | -6.56 | 0.51 | 0.26 |  |
| *Gm5844* | 2.048 | 3.80E-04 | -4.58 | -6.56 | 0.51 | 0.26 |  |
| *Atp5k-ps2* | 2.048 | 4.47E-04 | -4.58 | -6.56 | 0.51 | 0.26 |  |
| *E330017L17Rik* | 2.048 | 4.51E-04 | -4.58 | -6.56 | 0.51 | 0.26 |  |
| *Mir7212* | 2.048 | 8.00E-04 | -4.58 | -6.56 | 0.51 | 0.26 |  |
| *Gm17039* | 2.048 | 8.06E-04 | -4.58 | -6.56 | 0.51 | 0.26 |  |
| *Crybb1* | 2.048 | 8.98E-04 | -4.58 | -6.56 | 0.51 | 0.26 |  |
| *Col28a1* | 2.048 | 9.34E-04 | -4.58 | -6.56 | 0.51 | 0.26 | Diabetes, CVD |
| *Gm12774* | 2.048 | 1.37E-03 | -4.58 | -6.56 | 0.51 | 0.26 |  |
| *4930520O04Rik* | 2.048 | 1.39E-03 | -4.58 | -6.56 | 0.51 | 0.26 |  |
| *Gm29375* | 2.048 | 2.43E-03 | -4.58 | -6.56 | 0.51 | 0.26 |  |
| *Gm8822* | -2.048 | 5.42E-05 | -4.97 | -2.82 | 0.18 | 0.29 |  |
| *Trdn* | 2.046 | 1.23E-03 | -3.81 | -6.16 | 0.49 | 0.63 | Diabetes, CVD |
| *Tg* | 2.044 | 2.41E-03 | -4.00 | -5.98 | 0.57 | 0.42 | Obesity, Diabetes |
| *1700021N21Rik* | 2.044 | 3.58E-03 | -4.00 | -5.98 | 0.57 | 0.42 |  |
| *Snora78* | 2.044 | 1.44E-02 | -2.62 | -4.72 | 0.49 | 0.68 |  |
| *Gm11945* | 2.043 | 1.22E-03 | -4.18 | -6.16 | 0.64 | 0.50 |  |
| *Slc28a3* | 2.035 | 7.05E-04 | -4.18 | -6.16 | 0.48 | 0.26 | CVD |
| *Gm6139* | 2.035 | 2.23E-03 | -4.18 | -6.16 | 0.48 | 0.26 |  |
| *Gm26669* | 2.035 | 4.38E-03 | -4.18 | -6.16 | 0.48 | 0.26 |  |
| *Gm9294* | 2.035 | 7.20E-04 | -4.18 | -6.16 | 0.48 | 0.26 |  |
| *Rsph4a* | 2.035 | 4.07E-03 | -4.18 | -6.16 | 0.48 | 0.26 | CVD |
| *Gm13449* | -2.033 | 7.05E-04 | -4.39 | -2.32 | 0.50 | 0.32 |  |
| *Spata31d1b* | 2.026 | 1.37E-04 | -4.18 | -5.98 | 0.64 | 0.42 |  |
| *4933400F21Rik* | 2.026 | 3.45E-03 | -4.18 | -5.98 | 0.64 | 0.42 |  |
| *Gm8151* | 2.025 | 7.59E-05 | -3.79 | -5.77 | 0.35 | 0.29 |  |
| *Ap3s1-ps2* | 2.025 | 2.51E-02 | -2.79 | -4.76 | 0.82 | 0.49 |  |
| *Gm15372* | 2.015 | 2.99E-02 | -2.74 | -4.64 | 0.41 | 1.17 |  |
| *Gm13784* | 2.012 | 1.44E-03 | -4.00 | -6.16 | 0.42 | 0.43 |  |
| *Gm13680* | 2.011 | 8.69E-03 | -3.39 | -5.40 | 0.18 | 0.70 |  |
| *Gm20695* | 2.008 | 8.58E-03 | -3.60 | -5.58 | 0.56 | 0.61 |  |
| *Gm29488* | 2.005 | 4.86E-03 | -3.79 | -5.77 | 0.31 | 0.29 |  |
| *Gm14848* | 2.003 | 2.13E-03 | -4.00 | -6.16 | 0.59 | 0.63 |  |
| *Acta1* | 2.003 | 5.14E-03 | -3.48 | -5.77 | 0.69 | 0.54 | Diabetes, CVD |

**Table K. Significantly downregulated signaling and metabolism pathways due to maternal diet.** A negative logFC value indicates the pathway was downregulated in mice with high-fat-fed mothers.

| **Diet Comparison** | **KEGG_ID** | **Mean LogFC** | **FDR** |
| --- | --- | --- | --- |
| HF-HF ♀  vs.  LF-HF ♀  liver | mmu03010 Ribosome | -6.12 | 3.85E-07 |
|  | mmu03040 Spliceosome | -5.45 | 8.20E-06 |
|  | mmu00190 Oxidative phosphorylation | -3.90 | 4.36E-03 |
|  | mmu03013 RNA transport | -3.22 | 3.81E-02 |
| HF-HF ♂  vs.  LF-HF ♂ | mmu03010 Ribosome | -6.16 | 3.71E-07 |
|  | mmu03040 Spliceosome | -5.33 | 1.25E-05 |
|  | mmu00190 Oxidative phosphorylation | -5.27 | 1.25E-05 |
|  | mmu04210 Apoptosis | -5.01 | 2.82E-05 |
|  | mmu04145 Phagosome | -4.92 | 2.82E-05 |
|  | mmu04144 Endocytosis | -4.80 | 3.63E-05 |
|  | mmu04141 Protein processing in endoplasmic reticulum | -4.69 | 6.72E-05 |
|  | mmu04612 Antigen processing and presentation | -4.26 | 4.70E-04 |
|  | mmu03050 Proteasome | -4.29 | 7.68E-04 |
|  | mmu03008 Ribosome biogenesis in eukaryotes | -3.88 | 1.76E-03 |
|  | mmu04623 Cytosolic DNA-sensing pathway | -3.74 | 2.96E-03 |
|  | mmu04668 TNF signaling pathway | -3.63 | 2.96E-03 |
|  | mmu00230 Purine metabolism | -3.58 | 2.96E-03 |
|  | mmu00240 Pyrimidine metabolism | -3.60 | 2.96E-03 |
|  | mmu04062 Chemokine signaling pathway | -3.56 | 2.96E-03 |
|  | mmu03013 RNA transport | -3.33 | 5.56E-03 |
|  | mmu04620 Toll-like receptor signaling pathway | -3.34 | 5.56E-03 |
|  | mmu04622 RIG-I-like receptor signaling pathway | -3.36 | 5.56E-03 |
|  | mmu04060 Cytokine-cytokine receptor interaction | -3.29 | 5.56E-03 |
|  | mmu04120 Ubiquitin mediated proteolysis | -3.31 | 5.56E-03 |
|  | mmu04650 Natural killer cell mediated cytotoxicity | -3.31 | 5.56E-03 |
|  | mmu04064 NF-kappa B signaling pathway | -3.30 | 5.67E-03 |
|  | mmu04380 Osteoclast differentiation | -3.21 | 6.79E-03 |
|  | mmu04722 Neurotrophin signaling pathway | -3.00 | 1.29E-02 |
|  | mmu04666 Fc gamma R-mediated phagocytosis | -2.93 | 1.69E-02 |
|  | mmu04540 Gap junction | -2.88 | 1.83E-02 |
|  | mmu04142 Lysosome | -2.86 | 1.83E-02 |
|  | mmu03030 DNA replication | -2.94 | 1.86E-02 |
|  | mmu04662 B cell receptor signaling pathway | -2.85 | 1.91E-02 |
|  | mmu04915 Estrogen signaling pathway | -2.72 | 2.45E-02 |
|  | mmu04110 Cell cycle | -2.71 | 2.45E-02 |
|  | mmu00051 Fructose and mannose metabolism | -2.69 | 3.00E-02 |
|  | mmu04514 Cell adhesion molecules (CAMs) | -2.60 | 3.00E-02 |
|  | mmu04151 PI3K-Akt signaling pathway | -2.58 | 3.00E-02 |
|  | mmu00520 Amino sugar and nucleotide sugar metabolism | -2.64 | 3.00E-02 |
|  | mmu04621 NOD-like receptor signaling pathway | -2.58 | 3.21E-02 |
|  | mmu00010 Glycolysis / Gluconeogenesis | -2.55 | 3.38E-02 |
|  | mmu01200 Carbon metabolism | -2.52 | 3.38E-02 |
|  | mmu04390 Hippo signaling pathway | -2.50 | 3.43E-02 |
|  | mmu04130 SNARE interactions in vesicular transport | -2.53 | 3.73E-02 |
|  | mmu03015 mRNA surveillance pathway | -2.45 | 3.73E-02 |
|  | mmu04360 Axon guidance | -2.44 | 3.73E-02 |
|  | mmu04071 Sphingolipid signaling pathway | -2.44 | 3.73E-02 |
|  | mmu03420 Nucleotide excision repair | -2.47 | 3.95E-02 |
|  | mmu03410 Base excision repair | -2.46 | 4.06E-02 |
|  | mmu03020 RNA polymerase | -2.47 | 4.15E-02 |
|  | mmu04066 HIF-1 signaling pathway | -2.36 | 4.34E-02 |
|  | mmu04510 Focal adhesion | -2.31 | 4.61E-02 |
|  | mmu04910 Insulin signaling pathway | -2.31 | 4.65E-02 |
|  | mmu04015 Rap1 signaling pathway | -2.29 | 4.67E-02 |
|  | mmu04611 Platelet activation | -2.28 | 4.76E-02 |
| HF-LF ♀  vs.  LF-LF ♀ | mmu04110 Cell cycle | -4.55 | 5.58E-04 |
|  | mmu00190 Oxidative phosphorylation | -4.53 | 5.58E-04 |
|  | mmu03040 Spliceosome | -4.32 | 8.71E-04 |
|  | mmu04120 Ubiquitin mediated proteolysis | -4.10 | 1.38E-03 |
|  | mmu03030 DNA replication | -4.26 | 1.38E-03 |
|  | mmu00240 Pyrimidine metabolism | -4.04 | 1.38E-03 |
|  | mmu03050 Proteasome | -4.12 | 1.92E-03 |
|  | mmu04141 Protein processing in endoplasmic reticulum | -3.82 | 2.14E-03 |
|  | mmu01200 Carbon metabolism | -3.62 | 4.16E-03 |
|  | mmu00480 Glutathione metabolism | -3.43 | 8.85E-03 |
|  | mmu03420 Nucleotide excision repair | -3.47 | 8.94E-03 |
|  | mmu04623 Cytosolic DNA-sensing pathway | -3.39 | 8.94E-03 |
|  | mmu04144 Endocytosis | -3.06 | 1.79E-02 |
|  | mmu03010 Ribosome | -3.04 | 1.79E-02 |
|  | mmu00630 Glyoxylate and dicarboxylate metabolism | -3.17 | 1.79E-02 |
|  | mmu00970 Aminoacyl-tRNA biosynthesis | -3.10 | 1.79E-02 |
|  | mmu03460 Fanconi anemia pathway | -2.98 | 2.27E-02 |
|  | mmu00620 Pyruvate metabolism | -2.93 | 2.49E-02 |
|  | mmu03013 RNA transport | -2.85 | 2.49E-02 |
|  | mmu03410 Base excision repair | -2.92 | 2.49E-02 |
|  | mmu00230 Purine metabolism | -2.76 | 2.98E-02 |
|  | mmu03440 Homologous recombination | -2.81 | 3.32E-02 |
|  | mmu04130 SNARE interactions in vesicular transport | -2.76 | 3.85E-02 |
|  | mmu04114 Oocyte meiosis | -2.57 | 4.65E-02 |
|  | mmu04210 Apoptosis | -2.52 | 4.94E-02 |
|  | mmu03060 Protein export | -2.63 | 4.94E-02 |
|  | mmu03008 Ribosome biogenesis in eukaryotes | -2.53 | 4.94E-02 |
|  | mmu00520 Amino sugar and nucleotide sugar metabolism | -2.51 | 5.07E-02 |
| HF-LF ♂  vs.  LF-LF ♂ | mmu04144 Endocytosis | -7.17 | 3.07E-10 |
|  | mmu04151 PI3K-Akt signaling pathway | -7.03 | 3.07E-10 |
|  | mmu04110 Cell cycle | -7.00 | 1.30E-09 |
|  | mmu04120 Ubiquitin mediated proteolysis | -6.27 | 6.91E-08 |
|  | mmu04380 Osteoclast differentiation | -6.01 | 1.48E-07 |
|  | mmu04210 Apoptosis | -5.99 | 1.48E-07 |
|  | mmu04062 Chemokine signaling pathway | -5.86 | 1.66E-07 |
|  | mmu04015 Rap1 signaling pathway | -5.81 | 1.66E-07 |
|  | mmu04662 B cell receptor signaling pathway | -5.79 | 8.33E-07 |
|  | mmu04360 Axon guidance | -5.47 | 9.57E-07 |
|  | mmu03010 Ribosome | -5.36 | 2.44E-06 |
|  | mmu04620 Toll-like receptor signaling pathway | -5.40 | 2.44E-06 |
|  | mmu03460 Fanconi anemia pathway | -5.63 | 2.62E-06 |
|  | mmu04722 Neurotrophin signaling pathway | -5.28 | 2.62E-06 |
|  | mmu04510 Focal adhesion | -5.16 | 2.78E-06 |
|  | mmu03040 Spliceosome | -5.24 | 3.02E-06 |
|  | mmu04010 MAPK signaling pathway | -5.06 | 3.68E-06 |
|  | mmu04141 Protein processing in endoplasmic reticulum | -5.14 | 3.68E-06 |
|  | mmu04666 Fc gamma R-mediated phagocytosis | -5.13 | 6.51E-06 |
|  | mmu00230 Purine metabolism | -4.95 | 6.59E-06 |
|  | mmu04650 Natural killer cell mediated cytotoxicity | -4.93 | 9.13E-06 |
|  | mmu04145 Phagosome | -4.79 | 1.17E-05 |
|  | mmu04064 NF-kappa B signaling pathway | -4.88 | 1.17E-05 |
|  | mmu04150 mTOR signaling pathway | -4.70 | 1.90E-05 |
|  | mmu04060 Cytokine-cytokine receptor interaction | -4.63 | 2.03E-05 |
|  | mmu00240 Pyrimidine metabolism | -4.73 | 2.07E-05 |
|  | mmu04152 AMPK signaling pathway | -4.67 | 2.17E-05 |
|  | mmu04068 FoxO signaling pathway | -4.63 | 2.22E-05 |
|  | mmu03013 RNA transport | -4.56 | 2.81E-05 |
|  | mmu04910 Insulin signaling pathway | -4.57 | 2.81E-05 |
|  | mmu04668 TNF signaling pathway | -4.58 | 2.81E-05 |
|  | mmu04142 Lysosome | -4.53 | 3.49E-05 |
|  | mmu04070 Phosphatidylinositol signaling system | -4.55 | 3.49E-05 |
|  | mmu04014 Ras signaling pathway | -4.44 | 3.51E-05 |
|  | mmu04623 Cytosolic DNA-sensing pathway | -4.76 | 3.51E-05 |
|  | mmu04066 HIF-1 signaling pathway | -4.49 | 3.67E-05 |
|  | mmu03008 Ribosome biogenesis in eukaryotes | -4.56 | 3.67E-05 |
|  | mmu04610 Complement and coagulation cascades | -4.49 | 4.27E-05 |
|  | mmu04390 Hippo signaling pathway | -4.39 | 4.31E-05 |
|  | mmu04114 Oocyte meiosis | -4.40 | 4.40E-05 |
|  | mmu04611 Platelet activation | -4.24 | 8.45E-05 |
|  | mmu04919 Thyroid hormone signaling pathway | -4.20 | 9.90E-05 |
|  | mmu04071 Sphingolipid signaling pathway | -4.19 | 1.02E-04 |
|  | mmu04660 T cell receptor signaling pathway | -4.17 | 1.13E-04 |
|  | mmu00562 Inositol phosphate metabolism | -4.25 | 1.13E-04 |
|  | mmu04810 Regulation of actin cytoskeleton | -4.10 | 1.13E-04 |
|  | mmu03410 Base excision repair | -4.43 | 1.14E-04 |
|  | mmu04115 p53 signaling pathway | -4.14 | 1.34E-04 |
|  | mmu04514 Cell adhesion molecules (CAMs) | -4.04 | 1.45E-04 |
|  | mmu03030 DNA replication | -4.42 | 1.53E-04 |
|  | mmu04310 Wnt signaling pathway | -4.01 | 1.63E-04 |
|  | mmu04012 ErbB signaling pathway | -4.06 | 1.63E-04 |
|  | mmu04914 Progesterone-mediated oocyte maturation | -4.03 | 1.65E-04 |
|  | mmu03420 Nucleotide excision repair | -4.27 | 1.73E-04 |
|  | mmu00310 Lysine degradation | -4.18 | 1.90E-04 |
|  | mmu04146 Peroxisome | -3.98 | 2.14E-04 |
|  | mmu04550 Signaling pathways regulating pluripotency of stem cells | -3.77 | 3.73E-04 |
|  | mmu04640 Hematopoietic cell lineage | -3.78 | 4.02E-04 |
|  | mmu04740 Olfactory transduction | 4.74 | 4.08E-04 |
|  | mmu04915 Estrogen signaling pathway | -3.77 | 4.11E-04 |
|  | mmu03018 RNA degradation | -3.78 | 4.36E-04 |
|  | mmu01200 Carbon metabolism | -3.74 | 4.36E-04 |
|  | mmu03440 Homologous recombination | -3.91 | 4.82E-04 |
|  | mmu04340 Hedgehog signaling pathway | -3.80 | 5.54E-04 |
|  | mmu04922 Glucagon signaling pathway | -3.61 | 6.85E-04 |
|  | mmu00480 Glutathione metabolism | -3.63 | 8.52E-04 |
|  | mmu04350 TGF-beta signaling pathway | -3.49 | 1.01E-03 |
|  | mmu00860 Porphyrin and chlorophyll metabolism | -3.60 | 1.06E-03 |
|  | mmu04540 Gap junction | -3.47 | 1.06E-03 |
|  | mmu00510 N-Glycan biosynthesis | -3.62 | 1.08E-03 |
|  | mmu04520 Adherens junction | -3.44 | 1.16E-03 |
|  | mmu00071 Fatty acid degradation | -3.45 | 1.35E-03 |
|  | mmu04512 ECM-receptor interaction | -3.36 | 1.41E-03 |
|  | mmu04621 NOD-like receptor signaling pathway | -3.38 | 1.42E-03 |
|  | mmu00983 Drug metabolism - other enzymes | -3.43 | 1.42E-03 |
|  | mmu04130 SNARE interactions in vesicular transport | -3.56 | 1.46E-03 |
|  | mmu00190 Oxidative phosphorylation | -3.26 | 1.74E-03 |
|  | mmu03050 Proteasome | -3.35 | 2.17E-03 |
|  | mmu00532 Glycosaminoglycan biosynthesis - chondroitin sulfate / dermatan sulfate | -3.46 | 2.22E-03 |
|  | mmu04330 Notch signaling pathway | -3.21 | 2.50E-03 |
|  | mmu00970 Aminoacyl-tRNA biosynthesis | -3.26 | 2.60E-03 |
|  | mmu00500 Starch and sucrose metabolism | -3.18 | 2.77E-03 |
|  | mmu04670 Leukocyte transendothelial migration | -3.09 | 2.88E-03 |
|  | mmu00980 Metabolism of xenobiotics by cytochrome P450 | -3.12 | 2.88E-03 |
|  | mmu04370 VEGF signaling pathway | -3.10 | 3.14E-03 |
|  | mmu03015 mRNA surveillance pathway | -3.06 | 3.22E-03 |
|  | mmu04622 RIG-I-like receptor signaling pathway | -3.08 | 3.39E-03 |
|  | mmu03430 Mismatch repair | -3.29 | 3.76E-03 |
|  | mmu01212 Fatty acid metabolism | -2.98 | 4.47E-03 |
|  | mmu03320 PPAR signaling pathway | -2.94 | 4.47E-03 |
|  | mmu03020 RNA polymerase | -3.11 | 4.65E-03 |
|  | mmu00563 Glycosylphosphatidylinositol(GPI)-anchor biosynthesis | -3.14 | 4.67E-03 |
|  | mmu04022 cGMP-PKG signaling pathway | -2.82 | 5.74E-03 |
|  | mmu03022 Basal transcription factors | -2.90 | 5.74E-03 |
|  | mmu04270 Vascular smooth muscle contraction | -2.80 | 6.09E-03 |
|  | mmu04728 Dopaminergic synapse | -2.74 | 7.17E-03 |
|  | mmu04630 Jak-STAT signaling pathway | -2.72 | 7.41E-03 |
|  | mmu04912 GnRH signaling pathway | -2.74 | 7.41E-03 |
|  | mmu04916 Melanogenesis | -2.72 | 7.51E-03 |
|  | mmu00010 Glycolysis / Gluconeogenesis | -2.74 | 7.60E-03 |
|  | mmu04974 Protein digestion and absorption | -2.69 | 8.15E-03 |
|  | mmu04612 Antigen processing and presentation | -2.67 | 8.65E-03 |
|  | mmu04024 cAMP signaling pathway | -2.64 | 8.86E-03 |
|  | mmu00620 Pyruvate metabolism | -2.73 | 9.20E-03 |
|  | mmu00564 Glycerophospholipid metabolism | -2.61 | 9.86E-03 |
|  | mmu00040 Pentose and glucuronate interconversions | -2.66 | 1.01E-02 |
|  | mmu01230 Biosynthesis of amino acids | -2.61 | 1.01E-02 |
|  | mmu00640 Propanoate metabolism | -2.67 | 1.13E-02 |
|  | mmu04921 Oxytocin signaling pathway | -2.52 | 1.18E-02 |
|  | mmu00270 Cysteine and methionine metabolism | -2.55 | 1.24E-02 |
|  | mmu04725 Cholinergic synapse | -2.49 | 1.29E-02 |
|  | mmu00280 Valine, leucine and isoleucine degradation | -2.47 | 1.41E-02 |
|  | mmu04020 Calcium signaling pathway | -2.43 | 1.43E-02 |
|  | mmu04710 Circadian rhythm | -2.49 | 1.47E-02 |
|  | mmu04976 Bile secretion | -2.44 | 1.47E-02 |
|  | mmu04920 Adipocytokine signaling pathway | -2.42 | 1.51E-02 |
|  | mmu00520 Amino sugar and nucleotide sugar metabolism | -2.43 | 1.56E-02 |
|  | mmu04962 Vasopressin-regulated water reabsorption | -2.40 | 1.68E-02 |
|  | mmu00053 Ascorbate and aldarate metabolism | -2.44 | 1.69E-02 |
|  | mmu00982 Drug metabolism - cytochrome P450 | -2.35 | 1.77E-02 |
|  | mmu04750 Inflammatory mediator regulation of TRP channels | -2.30 | 1.91E-02 |
|  | mmu00514 Other types of O-glycan biosynthesis | -2.35 | 1.97E-02 |
|  | mmu00600 Sphingolipid metabolism | -2.32 | 1.97E-02 |
|  | mmu04917 Prolactin signaling pathway | -2.28 | 2.03E-02 |
|  | mmu04672 Intestinal immune network for IgA production | -2.27 | 2.15E-02 |
|  | mmu00900 Terpenoid backbone biosynthesis | -2.28 | 2.48E-02 |
|  | mmu03450 Non-homologous end-joining | -2.40 | 2.51E-02 |
|  | mmu04664 Fc epsilon RI signaling pathway | -2.18 | 2.54E-02 |
|  | mmu00051 Fructose and mannose metabolism | -2.19 | 2.61E-02 |
|  | mmu04970 Salivary secretion | -2.16 | 2.61E-02 |
|  | mmu00100 Steroid biosynthesis | -2.24 | 2.76E-02 |
|  | mmu00770 Pantothenate and CoA biosynthesis | -2.27 | 2.80E-02 |
|  | mmu00260 Glycine, serine and threonine metabolism | -2.13 | 2.92E-02 |
|  | mmu04730 Long-term depression | -2.05 | 3.30E-02 |
|  | mmu00561 Glycerolipid metabolism | -2.05 | 3.30E-02 |
|  | mmu00450 Selenocompound metabolism | -2.17 | 3.32E-02 |
|  | mmu00630 Glyoxylate and dicarboxylate metabolism | -2.09 | 3.32E-02 |
|  | mmu02010 ABC transporters | -2.05 | 3.33E-02 |
|  | mmu00830 Retinol metabolism | -2.03 | 3.33E-02 |
|  | mmu04140 Regulation of autophagy | -2.08 | 3.33E-02 |
|  | mmu00020 Citrate cycle (TCA cycle) | -2.06 | 3.46E-02 |
|  | mmu04918 Thyroid hormone synthesis | -1.98 | 3.65E-02 |
|  | mmu01040 Biosynthesis of unsaturated fatty acids | -1.99 | 3.94E-02 |
|  | mmu00670 One carbon pool by folate | -2.03 | 3.94E-02 |
|  | mmu04713 Circadian entrainment | -1.85 | 4.73E-02 |
|  | mmu04961 Endocrine and other factor-regulated calcium reabsorption | -1.86 | 4.80E-02 |
| HF-HF ♀  vs.  LF-HF ♀  heart | mmu04610 Complement and coagulation cascades | -6.80 | 3.61E-08 |
|  | mmu00830 Retinol metabolism | -6.39 | 1.96E-07 |
|  | mmu00140 Steroid hormone biosynthesis | -5.98 | 1.08E-06 |
|  | mmu03320 PPAR signaling pathway | -4.54 | 3.16E-04 |
|  | mmu00983 Drug metabolism - other enzymes | -4.12 | 1.93E-03 |
|  | mmu01230 Biosynthesis of amino acids | -3.80 | 3.67E-03 |
|  | mmu00590 Arachidonic acid metabolism | -3.76 | 3.93E-03 |
|  | mmu00053 Ascorbate and aldarate metabolism | -3.95 | 4.48E-03 |
|  | mmu00591 Linoleic acid metabolism | -3.51 | 9.72E-03 |
|  | mmu00260 Glycine, serine and threonine metabolism | -3.37 | 1.21E-02 |
|  | mmu00980 Metabolism of xenobiotics by cytochrome P450 | -3.31 | 1.21E-02 |
|  | mmu01200 Carbon metabolism | -3.24 | 1.21E-02 |
|  | mmu00010 Glycolysis / Gluconeogenesis | -3.24 | 1.21E-02 |
|  | mmu04151 PI3K-Akt signaling pathway | -3.04 | 1.63E-02 |
|  | mmu00040 Pentose and glucuronate interconversions | -3.17 | 1.63E-02 |
|  | mmu00982 Drug metabolism - cytochrome P450 | -3.07 | 1.68E-02 |
|  | mmu00860 Porphyrin and chlorophyll metabolism | -2.99 | 2.25E-02 |
|  | mmu00360 Phenylalanine metabolism | -2.91 | 3.41E-02 |
|  | mmu04918 Thyroid hormone synthesis | -2.62 | 5.04E-02 |
|  | mmu00120 Primary bile acid biosynthesis | -2.76 | 5.04E-02 |

**Fig A. Non-alcoholic fatty liver disease (NAFLD) pathway diagrams**. Orange indicates upregulation by a high-fat diet and blue indicates downregulation. (A) The NAFLD pathway is significantly downregulated due to an offspring high-fat diet. (B) It is also downregulated by a maternal high-fat diet. HF = High-fat diet, LF = Low-fat diet.

**A**


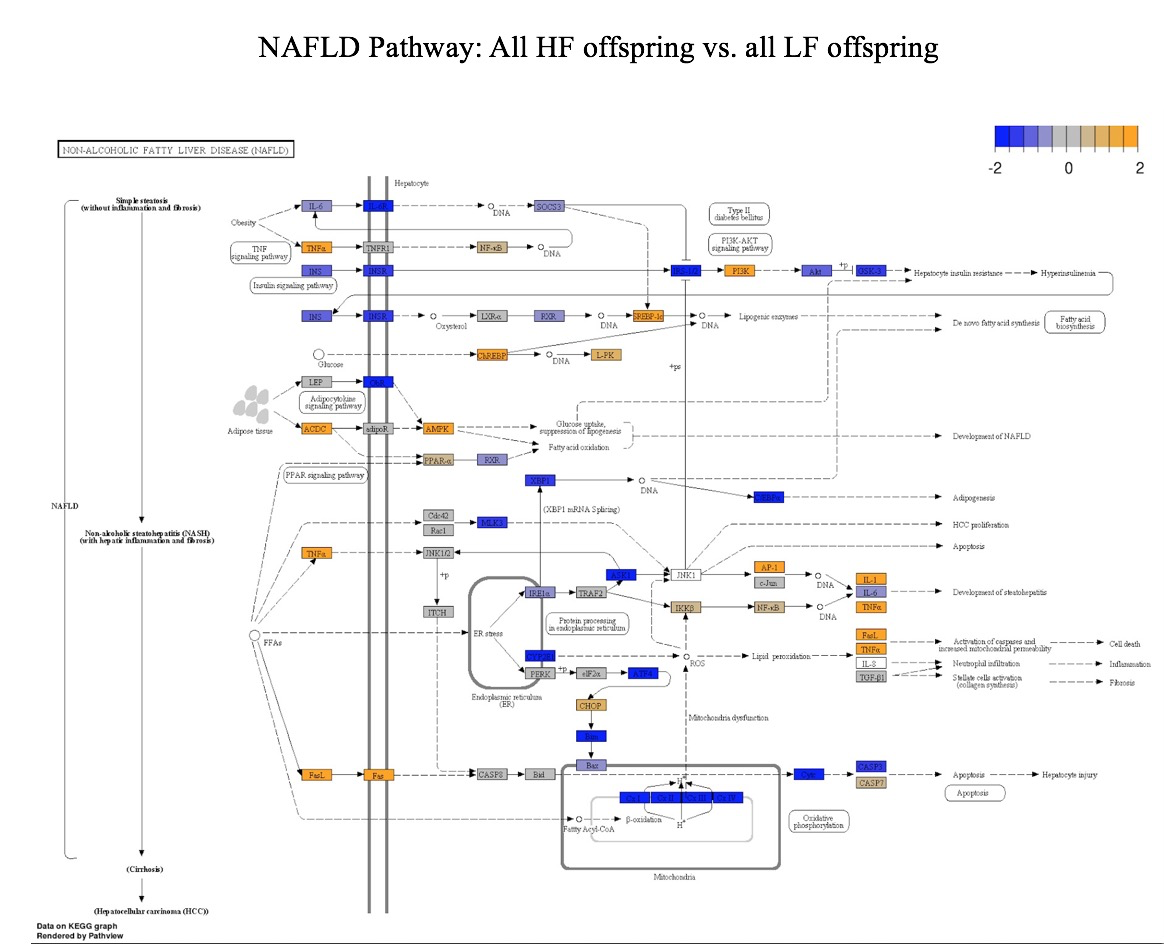


**B**


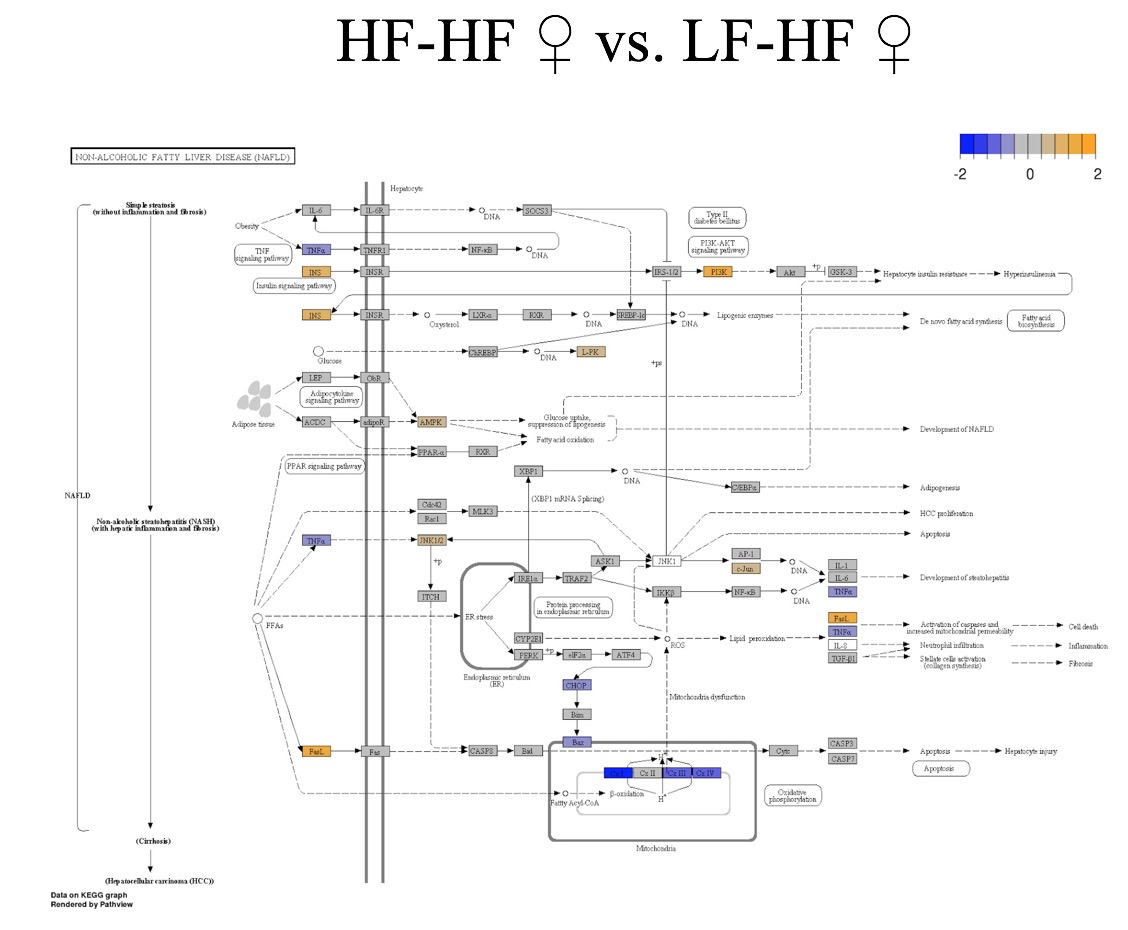

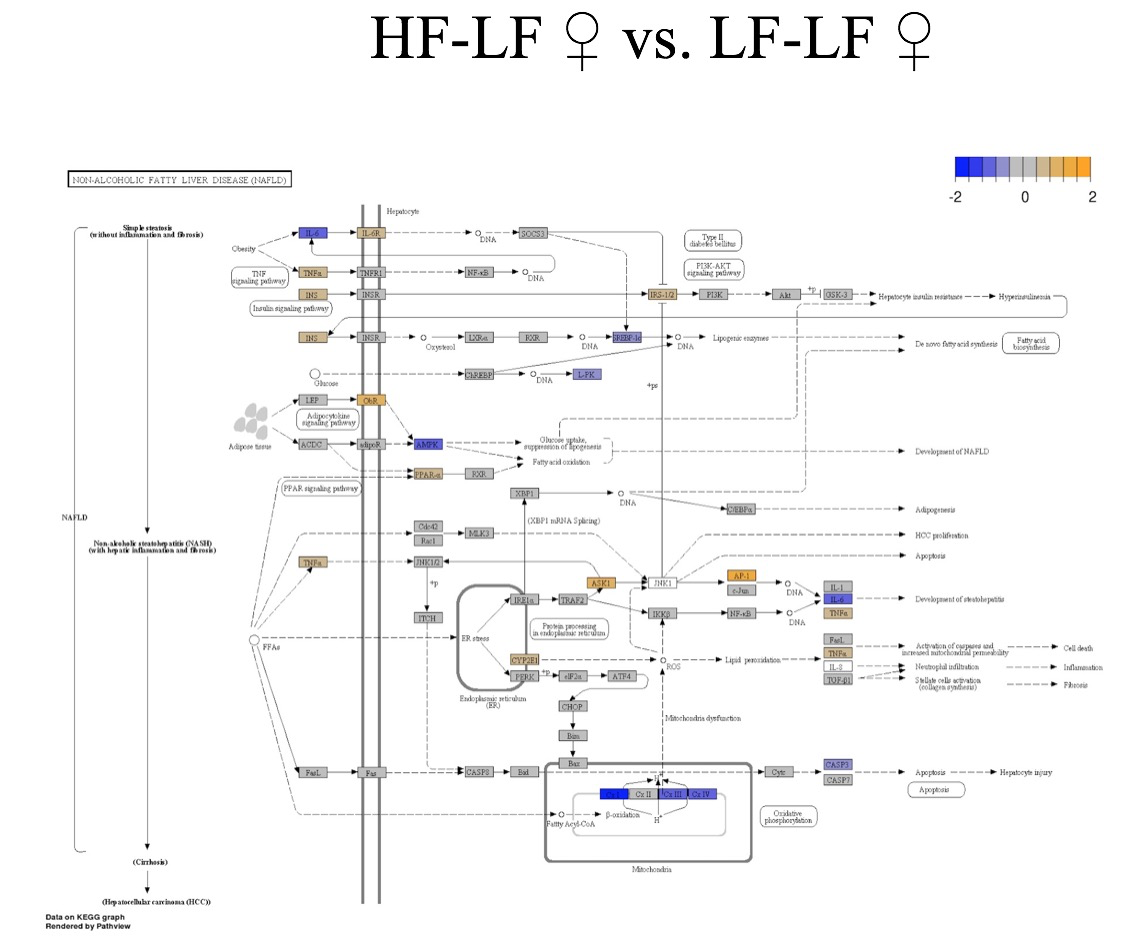


**Fig B. Alzheimer’s disease pathway diagrams.** Orange indicates upregulation by a high-fat diet and blue indicates downregulation. (A) The Alzheimer’s disease pathway is significantly downregulated due to an offspring high-fat diet. (B) It is also downregulated by maternal high-fat diet. HF = High-fat diet, LF = Low-fat diet.

**A**


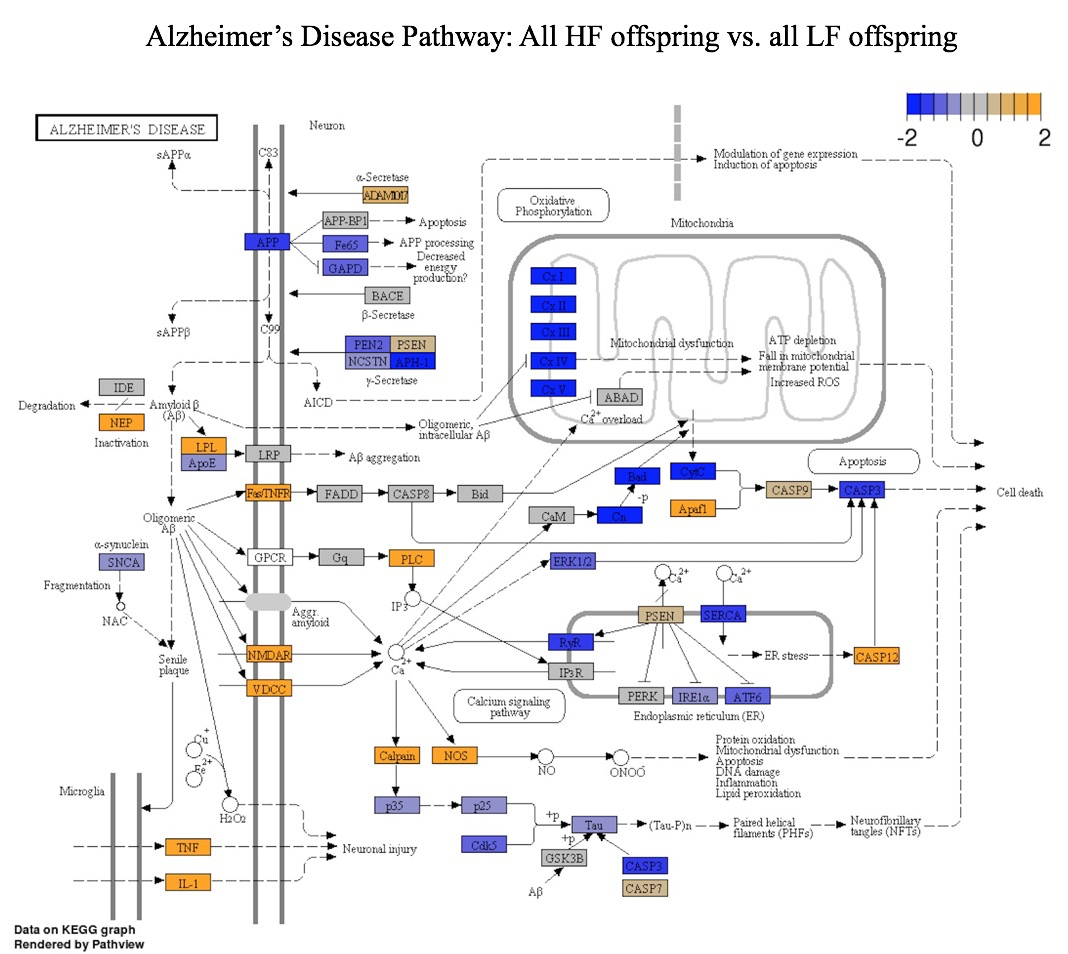


**B**


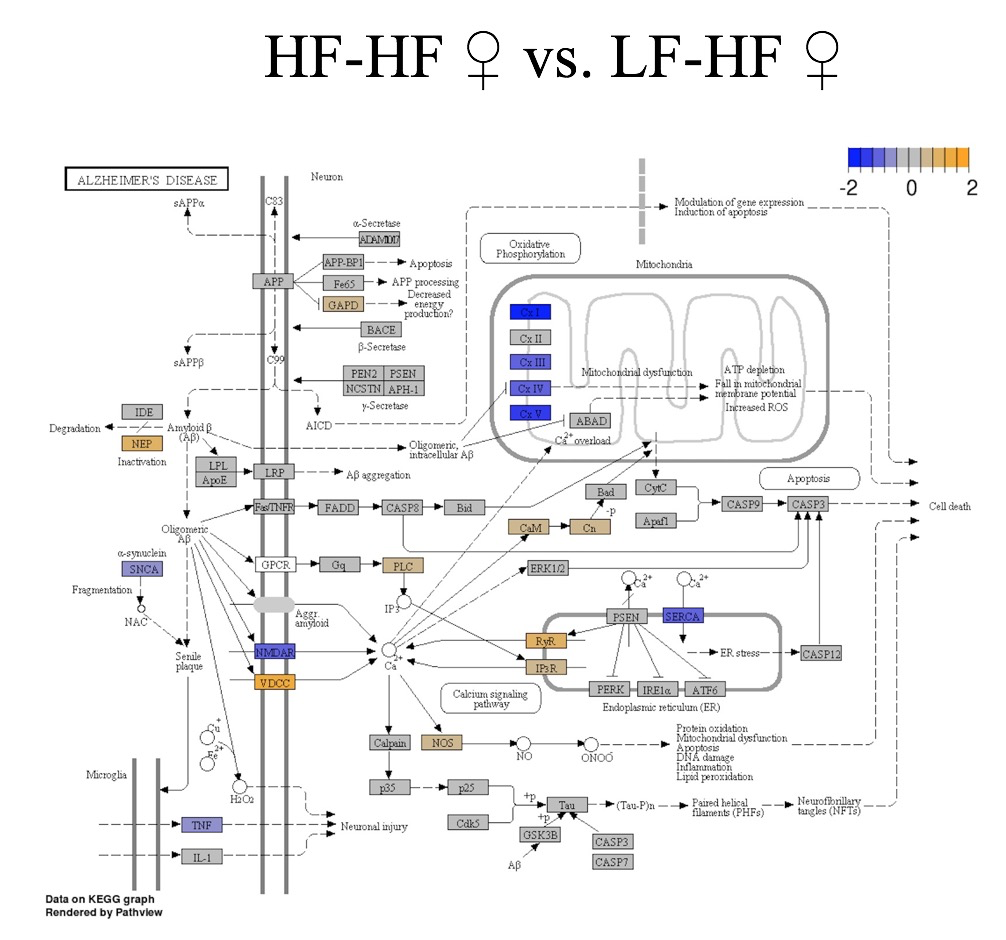

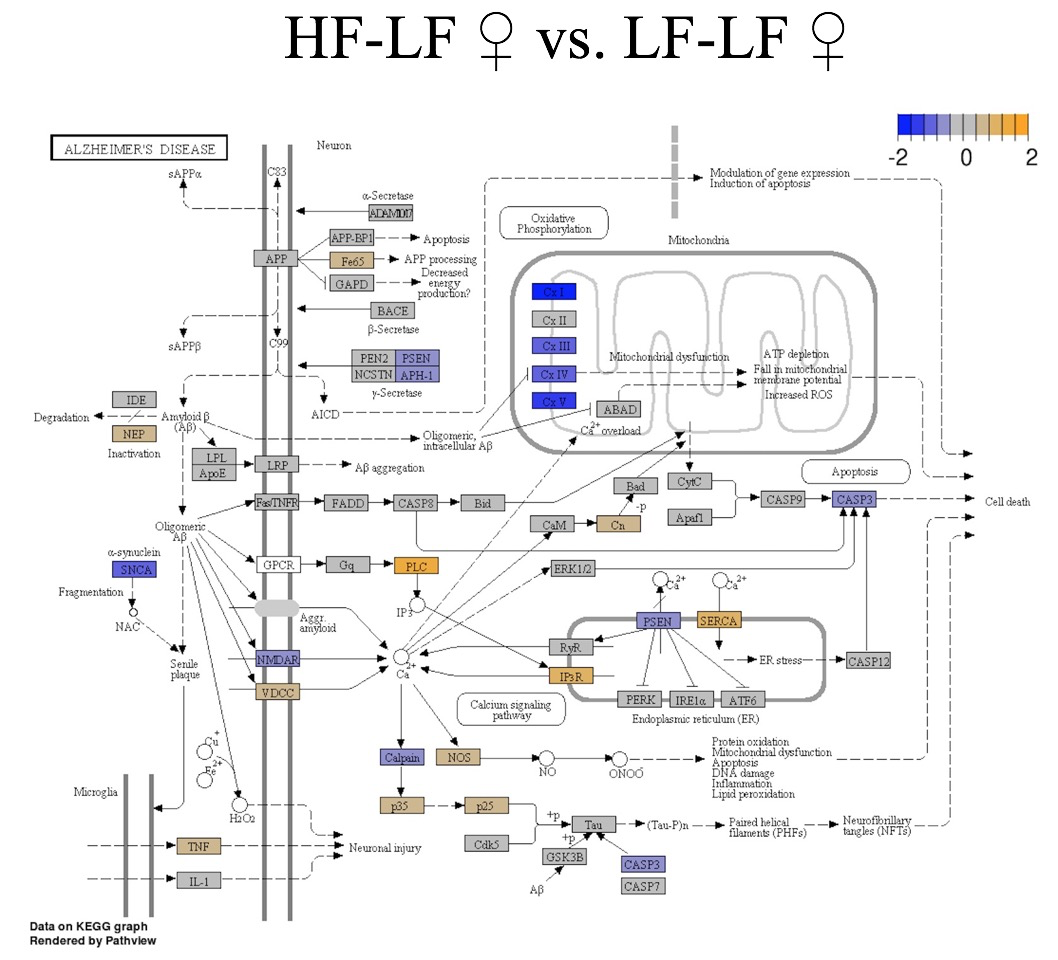


**Table L. Modules from WGCNA.**

| **Module**  **(# genes)** | **Enrichment p-value** | **# Module Genes in GO Term** | **GO ID** | **GO Ont** | **GO Name** |
| --- | --- | --- | --- | --- | --- |
| Black  (637) | 3.29E-19 | 145 | GO:0002376 | BP | immune system process |
|  | 9.95E-17 | 93 | GO:0006955 | BP | immune response |
|  | 2.83E-11 | 88 | GO:0002682 | BP | regulation of immune system process |
|  | 7.04E-09 | 83 | GO:0006952 | BP | defense response |
|  | 4.02E-08 | 59 | GO:0016337 | BP | single organismal cell-cell adhesion |
|  | 4.24E-07 | 53 | GO:0046649 | BP | lymphocyte activation |
|  | 5.17E-07 | 60 | GO:0098609 | BP | cell-cell adhesion |
|  | 7.73E-07 | 34 | GO:0009897 | CC | external side of plasma membrane |
|  | 1.26E-06 | 63 | GO:0001775 | BP | cell activation |
|  | 1.51E-06 | 58 | GO:0009986 | CC | cell surface |
| Magenta  (312) | 1.39E-05 | 16 | GO:0005840 | CC | ribosome |
|  | 1.75E-05 | 13 | GO:0005761 | CC | mitochondrial ribosome |
|  | 3.00E-04 | 12 | GO:0044391 | CC | ribosomal subunit |
|  | 0.003 | 17 | GO:0005759 | CC | mitochondrial matrix |
|  | 0.045 | 26 | GO:0030529 | CC | intracellular ribonucleoprotein complex |
|  | 0.046 | 12 | GO:0003735 | MF | structural constituent of ribosome |
|  | 0.355 | 6 | GO:0005763 | CC | mitochondrial small ribosomal subunit |
|  | 0.691 | 6 | GO:0005762 | CC | mitochondrial large ribosomal subunit |
|  | 0.691 | 6 | GO:0015935 | CC | small ribosomal subunit |
|  | 1 | 20 | GO:0006412 | BP | translation |
| Turquoise  (1,785) | 2.16E-12 | 33 | GO:0070469 | CC | respiratory chain |
|  | 1.02E-11 | 31 | GO:0005746 | CC | mitochondrial respiratory chain |
|  | 3.73E-08 | 41 | GO:0005840 | CC | ribosome |
|  | 1.31E-06 | 20 | GO:0005747 | CC | mitochondrial respiratory chain complex I |
|  | 1.31E-06 | 20 | GO:0045271 | CC | respiratory chain complex I |
|  | 7.96E-06 | 28 | GO:0005761 | CC | mitochondrial ribosome |
|  | 1.10E-04 | 14 | GO:0003954 | MF | NADH dehydrogenase activity |
|  | 1.47E-04 | 213 | GO:0005739 | CC | mitochondrion |
|  | 1.49E-04 | 16 | GO:0005763 | CC | mitochondrial small ribosomal subunit |
|  | 2.25E-04 | 35 | GO:0003735 | MF | structural constituent of ribosome |
| Yellow (932) | 4.41E-04 | 12 | GO:0070330 | MF | aromatase activity |
|  | 8.36E-04 | 16 | GO:0008395 | MF | steroid hydroxylase activity |
|  | 0.001 | 22 | GO:0004497 | MF | monooxygenase activity |
|  | 0.001 | 12 | GO:0016712 | MF | oxidoreductase activity, acting on paired donors, with incorporation or reduction of molecular oxygen, reduced flavin or flavoprotein as one donor, and incorporation of one atom of oxygen |
|  | 0.002 | 12 | GO:0008392 | MF | arachidonic acid epoxygenase activity |
|  | 0.002 | 10 | GO:0019373 | BP | epoxygenase P450 pathway |
|  | 0.005 | 12 | GO:0008391 | MF | arachidonic acid monooxygenase activity |
|  | 0.010 | 13 | GO:0019369 | BP | arachidonic acid metabolic process |
|  | 0.032 | 26 | GO:0016705 | MF | oxidoreductase activity, acting on paired donors, with incorporation or reduction of molecular oxygen |
|  | 0.108 | 20 | GO:0004866 | MF | endopeptidase inhibitor activity |

**Fig C. Plot from WGCNA analysis relating modules to the diabetes-related traits.** The WGCNA analysis revealed 29 modules of co-expressed genes in the offspring. Four of these modules (black, yellow, turquoise, and magenta) were significantly correlated with the diabetes-related traits: week 15 weight, week 16 weight, baseline glucose at week 15, area under the curve for the intraperitoneal glucose tolerance test, baseline glucose at week 16, area under the curve for the intraperitoneal insulin tolerance test, serum insulin and glucose, and average food consumed per day at 14 weeks of age.


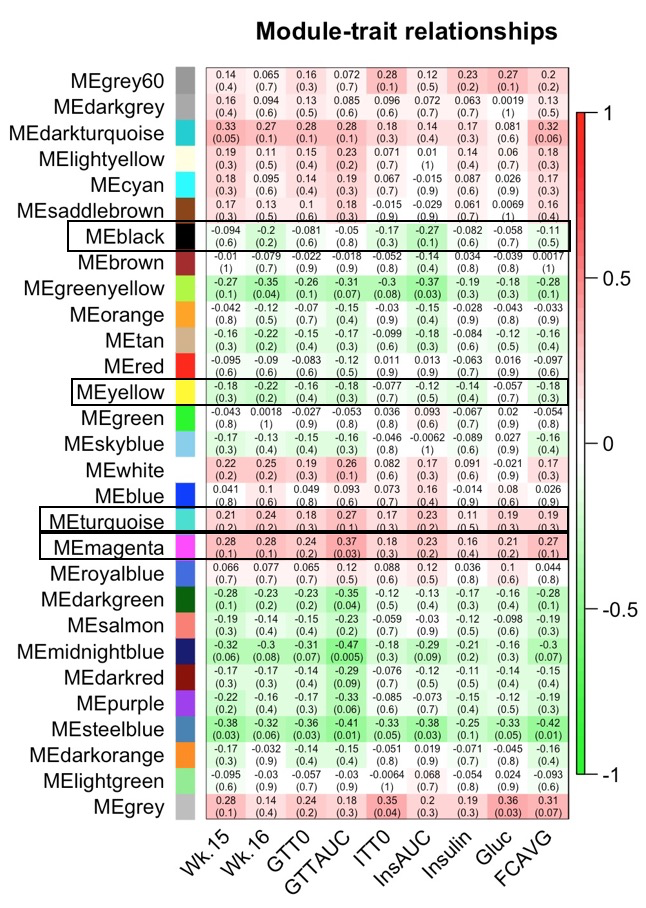


**Fig D. The expression levels of the *Mpo* and *Stat1* genes.** (A) Expression of the *Mpo* gene is higher in high-fat offspring than in low-fat offspring. (B) *Stat1* expression is also higher in high-fat offspring. (C) Compared to high-fat sons of low-fat mothers, high-fat sons of high-fat mothers have reduced methylation of the *Stat1* gene. HF = High-fat diet, LF = Low-fat diet, error bars represent ± standard error, N = 10 per group.

A)

B)

C)


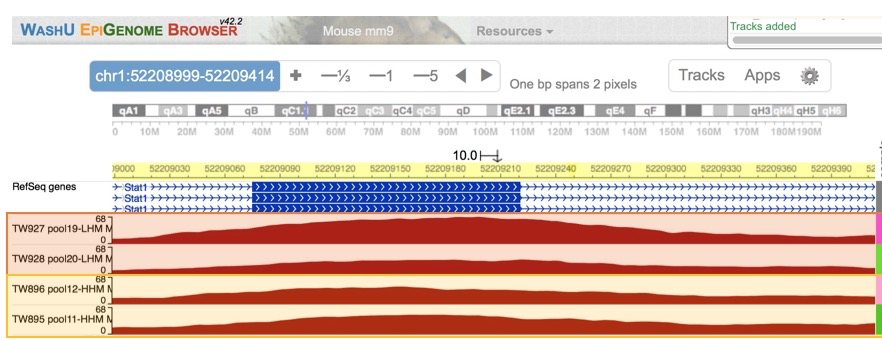


**Table M. Primers used for RT-qPCR.**

| **Gene** | **Forward primer** | **Reverse primer** |
| --- | --- | --- |
| *Adam11* | 5'-TGCTGCTGTTACCGCTTCT-3' | 5'-TCAGAGCCCTCTGGACTCTCT-3' |
| *Lad1* | 5'-ATGTCGGTCAGCAGAAAGGAC-3' | 5'-CTGTGGTTGAACTCAGGTTGC-3' |
| *Galnt10* | 5'-TGACCGATGCCGAGAGAGT-3' | 5'-AGAGAGCGATTCAGGGAGATT-3' |
| *Abcg8* | 5'-GTACGTGGGGTGTCCGGGGGTGAG-3' | 5'-GCGAGGCTGGTGGAGGGAGATGAG-3' |
| *Col1a1* | 5'-GCTCCTCTTAGGGGCCACT-3' | 5'-CCACGTCTCACCATTGGGG-3' |
| *Gapdh* | 5'-ACAATGAATACGGCTACAGCAACAG-3’ | 5’-GGTGGTCCAGGGTTTCTTACTCC-3’ |

**Table N. RT-qPCR validation results.** Values are presented as HF-HF expression relative to LF-HF expression levels. Fold differences were calculated with the ∆∆CT method and reported as a range to include the standard deviation. HF= High Fat, LF= Low Fat.

| Gene | Fold Difference of HF-HF vs. LF-HF daughters |
| --- | --- |
| *Anxa2* | 2.85 ± 1.21 |
| *Chrna4* | 5.84 ± 2.09 |
| *Mpo* | 12.96 ± 4.70 |
